# Solubility-Driven Design of Multicomponent Self-Assembled Hydrogels

**DOI:** 10.1021/acs.langmuir.6c03536

**Published:** 2026-09-17

**Authors:** Misthi Singh, Florian Trummer, Peter Schmiedel, Thomas Sottmann, Beatriu Escuder

**Affiliations:** † Institute of Advanced Materials (INAM), 16748 Universitat Jaume I, 12071, Castelló, Spain; ‡ Institute of Physical Chemistry, University of Stuttgart, Pfaffenwaldring 55, 70569, Stuttgart, Germany; § Research & Open Innovation-Material Science, Henkel AG & Co. KGaA, 40589, Dusseldorf, Germany

## Abstract

Nature has skillfully harnessed molecular social behavior, such as “co-assembly” and “self-sorting”, to achieve functional intricacy from simple building blocks. However, the rational control of such “social diversity” in synthetic supramolecular systems remains an unresolved problem. The entropy–enthalpy balance in hydrogelator self-assembly is a crucial thermodynamic parameter to control whether low molecular weight gelators (LMWGs) interact or act autonomously in multicomponent hydrogels. Here we propose that a simple inspection of the hydrogelator solubility vs temperature profile, as measured by ^1^H NMR, allows us to classify and select single-component hydrogelators in order to combine them at will. Thus, we demonstrate our hypothesis through an investigation of three individual LMWGs, with different solubility profiles, and their pairwise combinations to assess their assembly behavior over molecular, mesoscopic, and macroscopic length scales using a range of techniques. Our data show that one binary mixture exhibits co-assembly to form a unified network hydrogel material, whereas the other combinations display self-sorting, whereby each hydrogelator maintains its own structural integrity and intrinsic material properties. Crucially, temperature is used as an external stimulus to control these systems, providing direct and predictable control over material architecture. Our work highlights how simple NMR solubility measurements manifest the entropy–enthalpy balance as a rational approach to design multicomponent supramolecular materials with multifunctionality from simple and accessible molecular building blocks.

## Graphical abstract

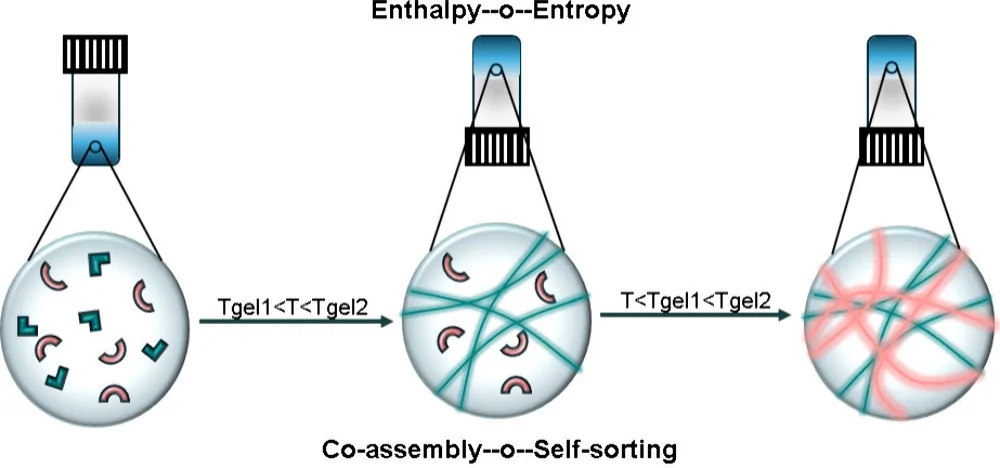

## Introduction

Multicomponent supramolecular gels, involving the self-assembly of several low molecular weight gelators (LMWGs), have emerged in the last years as complex materials with unique properties, including tunable mechanical behavior such as strain-stiffening, that single component gels cannot attain.^1–4^ Thus, multicomponent LMWGs have been reported as biomimetic matrices for cell cultures^5^ and regenerative medicine applications,^6^ as catalytic materials for tandem organic processes^7^ or as responsive drug delivery vehicles^8,9^ among others. A key feature for the design of those systems is the ability to predict and tune the assembly process to obtain gels with targeted structural and functional properties.^3,10^ For that purpose, the so-called social interaction among the individual components within the complex network must be considered. Gel components can self-sort to form (in)­dependent networks, co-assemble into a common gel network or disrupt each other’s assembly to a certain extent.^11,12^ While this variability provides an opportunity for practical applications, it can also appear as a challenge due to the need for the complete understanding of the factors that control their behavior and the collective properties of the new material.^13,14^

In many of the examples reported up to now, selected LMWG molecular scaffolds were used to promote either co-assembly or self-sorting of the two self-assembled networks. Thus, structural similarity at the molecular level would favor co-assembly, whereas structural dissimilarity promotes self-sorted networks.^15,16^ As an example of co-assembled networks, MacLachlan et al. recently reported a multicomponent mixture of amino acid-based LMWGs with tunable optical properties.^17^ As an example of self-sorted networks, Hamachi et al., prepared self-sorted two-component LMWGs by combining two low molecular weight compounds with dissimilar structure and described their application as responsive materials.^18^ Similarly, Brizard et al. demonstrated orthogonal self-assembly of a hydrogelator and a surfactant into independent nanostructured networks.^19^ Moreover, our group has used structural dissimilarity to self-sort LMWGs with potentially incompatible functional groups, thereby enabling their simultaneous catalytic activity in tandem organic reactions.^7^ Conversely, organocatalysis has also been employed to mediate and direct supramolecular self-assembly pathways.^20^ In addition, we have also shown that a hydrogel can be used to *in situ* catalyze the formation of a second self-sorted network.^21^ On the other hand, a different approach to obtain self-sorted two-component networks, introduced by Adams, consists of using two compounds with similar molecular scaffolds, but differing pKa values. This allows the pH-controlled, separate self-assembly of both LMWGs into their respective network.^22,23^ This remarkable example reveals that the temporal control of gelation (slow changes of pH, different kinetics of aggregation) is a valuable tool in a design-to-formulation approach of multicomponent gels.^24^ Recently, the same group has shown that with sample pH annealing it is possible to interconvert co-assembled networks into self-sorted ones, revealing the role of dynamics in supramolecular self-assembled systems.^25^ Moreover, Singh et al. have reported the use of chemical fuels to convert a three-component co-assembled LMWG system into a self-sorted one, based on slight differences in reactivity. They have also highlighted the template effect of the first network to be formed on the subsequent networks, revealing another possibility for spatiotemporal control of the material properties.^7,26^ All these examples have shed light onto the need for a rational control of multicomponent LMWGs self-assembly to prepare “à la carte” supramolecular materials for applications in complex settings.

An equally promising approach to advance these systems is through the combination of gelators with different temperature sensitivities, allowing multicomponent hydrogels to exhibit varied, thermally tunable properties.^27–29^ This can be especially useful in applications that require temperature-controlled responses, for example in drug delivery,^8^ where the system can be triggered to release therapeutics through programmed changes in temperature. Other applications can be found in tissue engineering, or in smart materials capable of adapting to various environmental conditions.^30^ Indeed, the development of temperature-responsive hydrogels would provide highly versatile materials and thus have a profound impact on many fields.^25,31^

Here we aim to provide a thermodynamic rationale for the strategic design of multicomponent networks using thermally controlled pathways. This strategy is based on the determination of thermodynamic parameters – enthalpy and entropy – for single component gel self-assembly, followed by their combination into socially diverse dual networks according to their respective temperature responsiveness – namely, their individual and collective balances of self-assembly enthalpy/entropy. As we reported previously,^32^ the solubility-temperature profile for a given hydrogelator provides valuable information on the thermodynamics of gel self-assembly. Thus, when solubility increases with temperature, the self-assembly process is governed by enthalpy, and temperature-dependent non-covalent interactions are involved. On the other hand, if solubility does not change dramatically with temperature, the self-assembly process is triggered by entropy, and the gel becomes temperature insensitive. Such behavior is the typical situation for highly hydrophobic gelators in aqueous media and is mainly related to the entropic component of the hydrophobic effect.^32^ Therefore, we envisage that thermally controlled multicomponent hydrogels may be prepared by judicious selection of components based on their individual solubility profiles. Although differences in T_gel_ have been intuitively used in the past to prepare two-component materials, the current approach offers a quantitative element for *a priori* design of multicomponent hydrogels.

## Experimental Section

### Gel Formation

Hydrogels were prepared by heat–cool technique. The appropriate amount of gelator was weighed in a standard 4.0 mL vial, and then 1.0 mL of distilled water was added to the vial. This mixture was sealed and heated to dissolve completely the gelator, and the obtained solution was cooled down undisturbed to the ambient temperature (ca. 25 °C) without controlling the cooling rate. All gels were prepared in identical ambient conditions. Gel formation was checked by standard vial inversion technique.

### Minimum Gel Concentration (MGC)

The MGC was calculated by preparing gels of different concentrations of gelator as described above and finding out the lowest concentration where a self-standing gel was formed.

### Sample Preparation for cryo-TEM

For preparing samples of the vitrification type for cryo-TEM, about 3 μL of sample solution was applied on a 300 mesh Quantifoil copper grid (pre-glowed before use) in the environmental chamber of the Vitrobot, and excess liquid was removed. Further, the grid was vitrified by dipping into liquid ethane and placed into the cryo holder at the liquid nitrogen temperature (∼-170 °C).

### Preparation of Samples for NMR

In the case of kinetic measurements, freshly prepared hydrogel was transferred to a co-axial NMR tube without delay. When working with solubility, hydroquinone was added to the inner capillary of the co-axial NMR tube and served as the internal standard; the samples were then allowed to age at room temperature for 10 h before analysis.

### Preparation of Samples for SAXS

Gel precursor solutions heated to 60 °C were poured into pre heated mark glass capillaries with a 1.98 mm optical path length, closed and gelled at room temperature before being measured. All samples were analyzed at 25 °C.

### Preparation of Samples for DSC

Gelator samples (2–3 mg) were weighed directly into Tzero aluminum crucibles and hermetically enclosed.

### Preparation of Samples for Rheology Measurements

Hydrogels were prepared according to the stated method, and the hot precursor mixture was placed directly onto the parallel plates of the rheometer (steel parallel plates with a diameter of 40 mm and a 500 μm gap). The sample was protected with a solvent trap of the device and was left at 25 °C.

## Results and Discussion

### Single-Component Selection

For our proof-of-concept study, three model hydrogelators were selected (see Figure 1). Compound **1** is a pseudopeptide based on l-valine with 4-pyridine carboxamide end groups that has been previously studied by our group and is known to self-assemble into hydrogels driven by the hydrophobic effect.^32^ Compound **2** is a bisurea-based hydrogelator, bearing polar groups at the ends, that has also been reported as an efficient hydrogelator and, in this case, is known to be highly temperature-responsive.^33^ These two compounds are bolaamphiphiles with a large difference in their hydrophobicity (clogP values of 1.9 and 0.5, respectively), and a relative structural dissimilarity. Finally, compound **3** is an l-glutamic acid-based pseudopeptide flanked with benzyloxycarbonyl N-terminal groups. This compound has been designed to share a close structural similarity with compound **1** but includes two carboxylic groups which slightly decrease the hydrophobicity of the molecule (clogP value of 1.6) and introduce the possibility of additional enthalpy-driven, non-covalent interactions (ionic, H-bonding).

**Figure 1:**
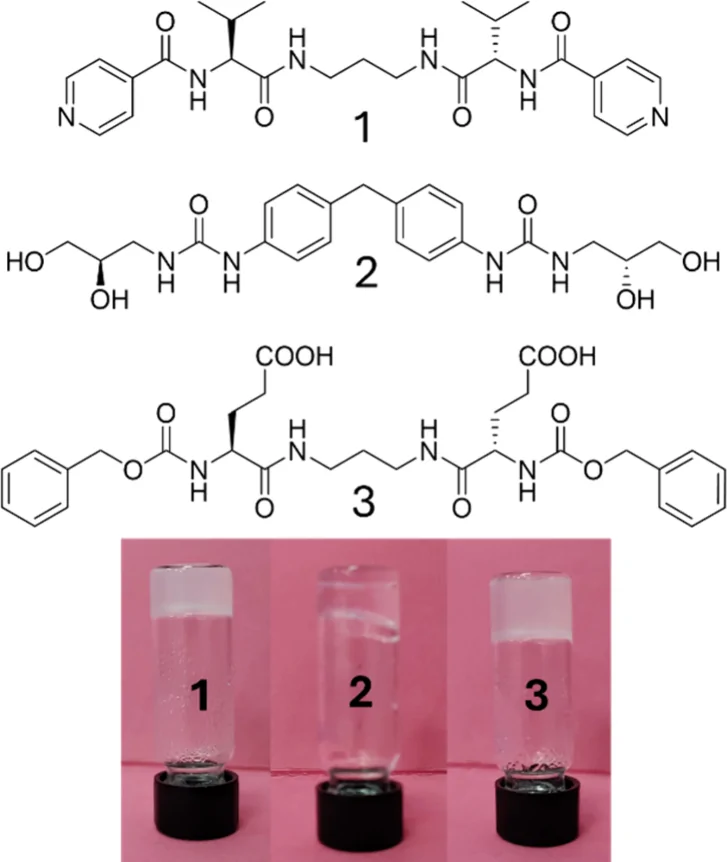
Molecular structure of compounds **1–3** and images of the macroscopic aspect of their hydrogels at minimum gel concentrations (**1**: 14 mM, **2**: 2.5 mM, **3**: 8.3 mM).

As mentioned before, the first step of our approach consists of determining the self-assembly parameters for the single-component systems (see Table 1). In particular, the solubility of each compound has been determined by ^1^H NMR. This technique provides a powerful tool to study sol-gel equilibria and gives interesting information on a gelator’s solubility and gel dynamics.^34,35^ It is well known that for a sol-gel equilibrium involving LMWGs, molecules in an aggregated state present restricted motion and therefore their signals appear broadened or absent in ^1^H NMR, while soluble monomers in the sol phase give a distinct signal.^34,35^ If the sol-gel exchange is fast on the time scale of the chemical shift, especially sharp signals appear that can be easily used for quantification.^36,37^ Therefore, this technique allows to determine the concentration of dissolved gelator monomers in equilibrium with the aggregated phase, which corresponds to the solubility constant. Thus, the solubility of each compound in water has been determined for a temperature range between 30 °C and 80 °C (Figure 2A), and the thermodynamic parameters have been calculated for the gel-to-solution equilibria at 30 °C (see Table 1 and Supporting Information for details).

**Table 1:** Thermodynamic Study for Single-Component Hydrogels^a^

Comp	clogP	MGC mM	*S* ^b^ mM	Δ*H* ^c^, kJ/mol	*T*Δ*S* ^c^, kJ/mol	Δ*G* ^c^, kJ/mol
**1**	1.9	14	9.3	1.5	-10.3	11.8
**2**	0.5	2.5	0.4	37.8	17.2	20.6
**3**	1.6	8.3	1	36.8	19.4	17.4

a *T* = 30 °C.

b Determined by ^1^HNMR (three replicas).

c Calculated for gel-to-sol equilibria.

**Figure 2:**
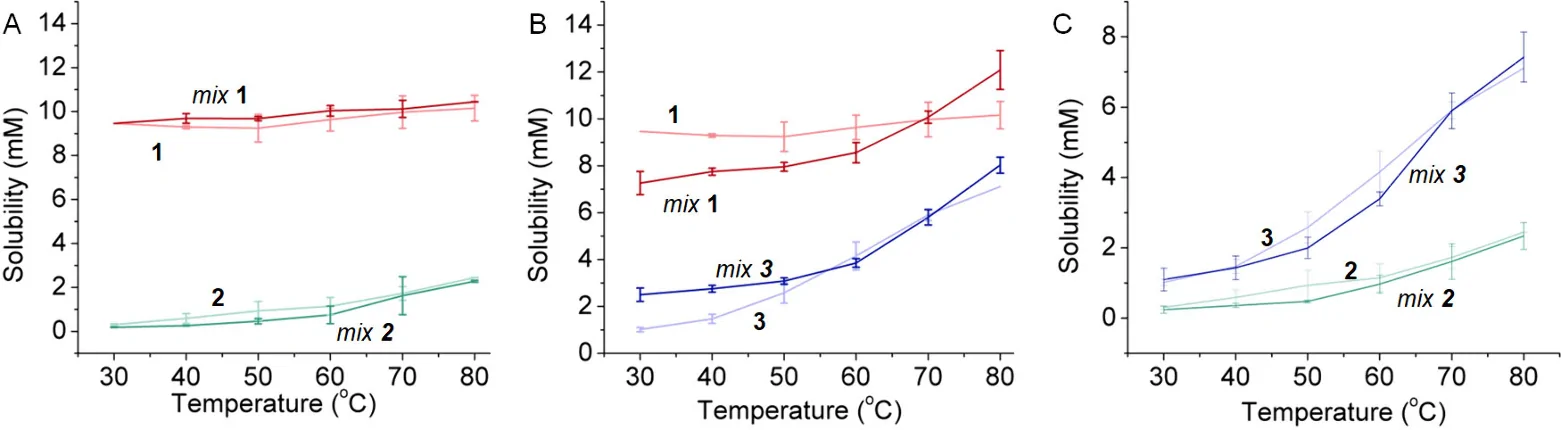
Solubility profiles obtained from temperature-dependent ^1^H-NMR. A) **1**, **2**, mix **1 + 2**. B) **1**, **3**, mix **1 + 3**. C) **2**, **3**, mix **2 + 3** at the respective minimum gelation concentration (MGC) of each gelator in water (D_2_O, 400 MHz).

Values for the enthalpic and entropic contribution to the gel-sol equilibrium at 30 °C are shown in Table 1, leading to positive values of ΔG which means that the reverse process of hydrogel self-assembly is thermodynamically favorable for the three compounds. However, a closer look to the respective thermodynamic contributions of enthalpy and entropy reveals that for compound 1 the entropic term holds the major component for hydrogel formation. This is in line with previous studies that point to a major contribution of the hydrophobic effect in self-assembly and correlates with the highest hydrophobicity (clogP 1.9) compared with the other two gelators.^32^ On the other hand, compounds **2** and **3** reveal a major contribution of enthalpy and an unfavorable entropic term. In the case of gelator **2**, with the lowest clogP value, it appears that the enthalpic term compensates for the unfavorable entropic term in the absence of a strong hydrophobic effect. On the other hand, gelator **3** shows structural similarity and a not so different clogP than gelator **1**. However, the presence of two carboxylate groups, polar and ionizable, depletes the hydrophobic effect and contributes to reinforcing enthalpic interactions, leading to an enthalpy-driven hydrogel.

DOSY NMR studies were performed in order to confirm the fast exchange of gelator monomers with the hydrogel phase and the absence of oligomers in solution, typical of highly cooperative self-assembled gels (Figure S3-8).^38^ Thus, the diffusion coefficients observed for compounds **1-2** are independent of concentration which confirms the absence of detectable species other than monomers in solution (**1**: *D* = 4.4 × 10^–10^ m^2^ /s; **2**: *D* = 3.7 × 10^–10^ m^2^ /s). For compound **3**, below the MGC, the diffusion coefficient is similar (*D* = 4.8 × 10^–10^ m^2^ /s), however at the MGC it slightly increases to 9 × 10^–10^ m^2^ /s, revealing a different behavior that could be related to changes in the solvation of carboxylates in the presence of the fibrillar network.

In addition, the temperature-dependent solubility profiles also reflect the thermodynamics of the hydrogel self-assembly process (Figure 2). Thus, an increase in solubility with temperature reveals an important contribution of enthalpy driven interactions. This is the case of compounds **2** and **3**, which exhibit a low and intermediate hydrophobicity, respectively, and that self-assemble through H-bonding involving urea functional groups for **2** and amide and carboxylic acid groups for **3**. In the case of compound **1**, as previously described, the invariance of solubility with respect to the temperature reveals a remarkable entropic contribution related to the increased hydrophobicity of this compound which enhances the contribution of the hydrophobic effect.^32^

Further insights into the networks formed by the three gelators were obtained through cryogenic-transmission electron microscopy (Cryo-TEM) and small angle X-ray scattering (SAXS). The cryo-TEM image of hydrogel **1** shows the presence of twisted fibrils with a diameter of 10 ± 2 nm (Figure 3A), which is in close agreement with the recorded SAXS data. As can be seen in Figure 4, the scattering intensity decreases with *q* ^–1.6^ at low scattering vectors q and shows Porod scattering (∝ *q* ^–4^) at high *q* indicating a scattering from flexible, self-avoiding fibers. Accordingly, the data could be almost quantitatively described by a polydisperse, flexible cylinder model with a mean diameter of 11±4 nm (and a Kuhn length of ca. 9 nm). Note that the length of the fibers is not resolvable in a SAXS experiment, as the largest length scale *D*_max_ is determined by the lowest accessible *q*min through the Fourier sampling theorem (*D*_max_ = π/*q*_min_), giving *D*_max_ ≈ 80 nm for our setup. In contrast, the scattering data of hydrogel **2** (Figure 4) shows two distinguished form factor oscillations at *q* = 0.056 and 0.124 Å ^–1^ together with a low *q* scattering proportional to *q* ^–2^, consistent with the existence of objects with a core-shell structure.

**Figure 3:**
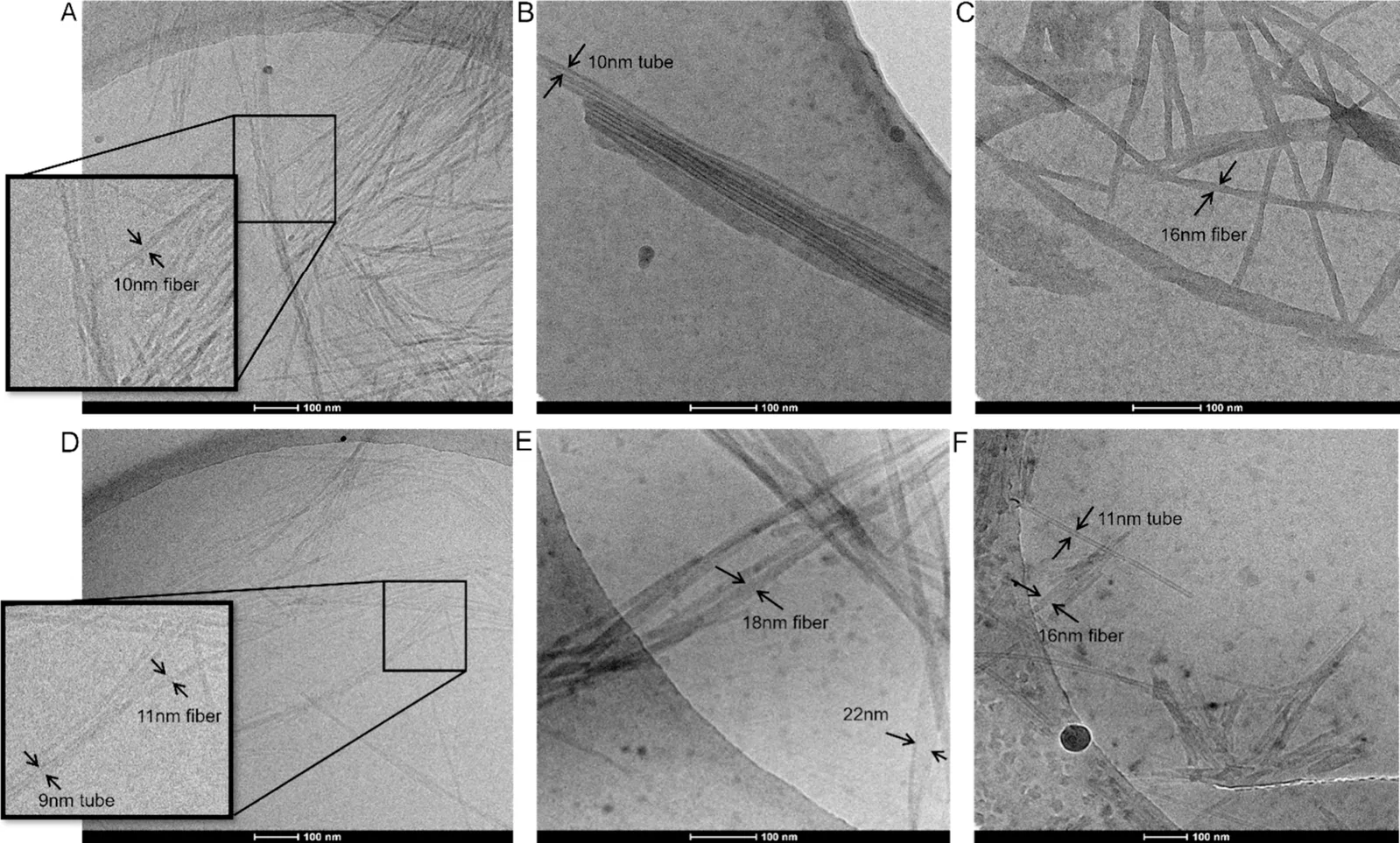
Cryo-TEM images of single-component hydrogels (A: **1**; B: **2**; C: **3**) and two-component mixtures (D: mix **1 + 2**; F: mix **2 + 3**) at the MGC for each gelator in water and (E: mix **1 + 3**) at the MGC of gelator **3** (8.3 mM) for each component. All the images are at a scale bar of 100 nm.

**Figure 4:**
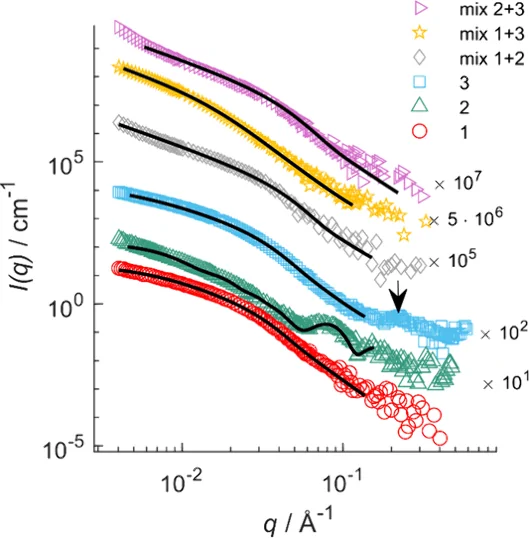
SAXS data of the single- and multicomponent hydrogels. The black lines represent the model curve for each dataset. The modelling approaches are described in the main text. Scattering intensities have been multiplied by arbitrary factors, displayed next to the curves, for better visualization. The arrow indicates a Bragg peak at q* = 0.223 Å^–1^.

A comprehensive analysis of the data via Indirect Fourier Transformation (IFT) and subsequent deconvolution into a cross-sectional electron density profile^39,40^ (discussed in the SI) revealed the existence of flat, hollow ribbons with a cross-sectional width of ca. 60 nm, a mean thickness of 10 nm and a wall thickness around 2 nm. The SAXS data and their analysis are strongly supported by the cryo-TEM image of hydrogel **2** (Figure 3B), which clearly shows bundled hollow tubular fibers with a thickness of approximately 11 nm with a wall thickness of ∼2 nm. Similarly, the cryo-TEM image and the SAXS data of hydrogel **3** are in agreement. Gelator **3**, like gelator **1**, forms compact fibers with some degree of twisting. The cryo-TEM image reveals fibers with an average thickness of 16 ± 2 nm (Figure 3C) and the SAXS data (Figure 4) of hydrogel **3** again show the typical scattering behavior of flexible cylinders (with *I* ∝ *q* ^–1.6^ at low *q*) yielding a mean fiber diameter of 10±4 nm (and a Kuhn length of 4 nm). In addition, a Bragg peak could be observed at q* = 0.223 Å ^–1^ that corresponds to a layer distance *d* = 2πq*/ ≈ 28.2 Å and could be shown to occur also in a powder diffraction measurement of the raw gelator **3**, as shown in the SI. Additional insights were gained from rheological measurements to examine the viscoelastic characteristics of hydrogel samples,^41^ especially their temperature-dependent behavior and fluid-to-solid transitions. Amplitude sweeps demonstrated that all gels exhibit typical viscoelastic behavior (loss factor tan­(δ) < 1), though with differing resistance to strain. Hydrogel **2** showed the highest yield strain (0.8%), consistent with its rigid morphology, while hydrogel **3** and **1** (0.53% and 0.5% respectively) displayed more elastic behavior (Figure S10). Oscillatory frequency sweep rheology (strain amplitude in linear viscoelastic region, 25 °C) for single-component hydrogels (5A-C). Closed symbols: storage modulus *G*′, open symbols: loss modulus *G*″. The *G*′ > *G*″ relationship for all frequencies is consistent with viscoelastic solid behavior for all three materials. Gelator **1** (Figure 5A) and Gelator **3** (Figure 5C) have strong frequency-independent elastic materials (*G*′ ∼ 10^4^ Pa), while Gelator **2** (Figure 5B) has a much weaker material (*G*′ ∼ 10^1^–10^2^ Pa) with noisy and non-plateau-like behavior suggestive of poor network formation at the gel boundary. Temperature ramps revealed that hydrogel **2** is the least thermally stable one, losing its network structure entirely at 43 °C, while hydrogels **1** and **3** remained mechanically stable at temperatures above 60 °C (Figure S11A–C). Furthermore, gelator **1** displays entropy-driven behavior, as demonstrated by the progressive increase in *G*′ as the temperature increases (Figure S11A).^42^

**Figure 5:**
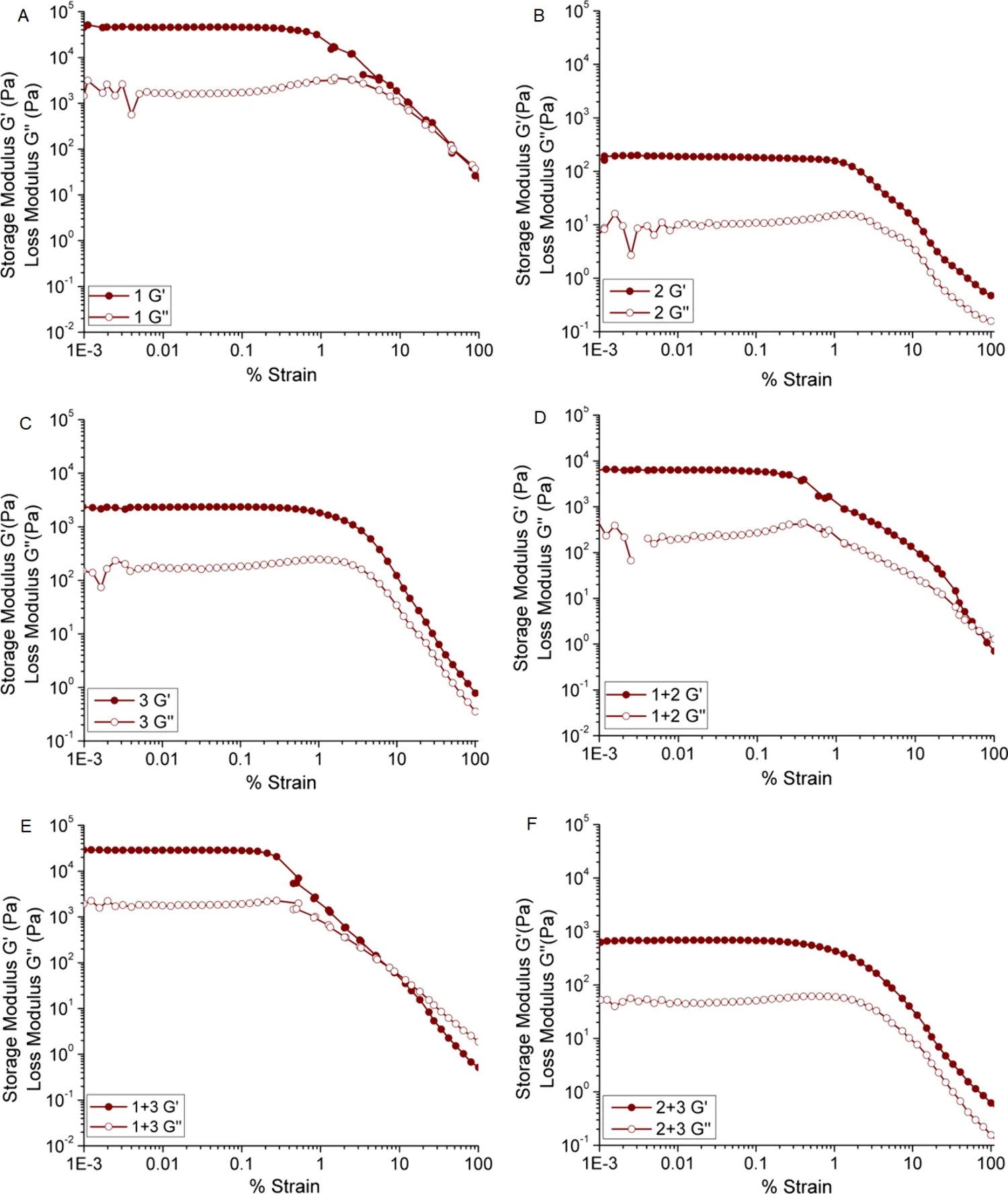
Frequency sweep of single-component hydrogel and mixture of gelators in hydrogel at their respective minimum gelation concentration, 25 °C, measured after 1 h of gel formation. A) **1** at 0.025% strain. B) **2** at 0.003% strain. C) **3** at 0.016% strain. D) mix **1 + 2** at 0.04% strain E) mix **1 + 3** at 0.045% strain F) mix **2 + 3** at 0.016% strain. The decrease in G’ for the first 2–3 points is the machine limit at 0.001 Hz frequency. For gelator **2** G’ is very low but constant throughout the frequency, indicating a very soft gel.

### Tailored Formulation of Multicomponent Hydrogels

Based on the thermodynamic studies of the three single component hydrogels, we have developed three multicomponent systems. The first one involves gelator **1** and **2** (*mix* **1 + 2**) and aims to obtain a self-sorted network in which fibers of hydrogel **1**, as an entropic gel, are maintained even at high temperatures (80 °C) and a co-existing network of hydrogel **2**, as an enthalpy driven hydrogel, will be assembled after cooling to 30 °C. The respective concentrations of both components have been chosen in a way to start at high temperatures, above the solubility of compound **1** and below the solubility of compound **2**. In addition, after the cooling to 30 °C, the concentration of compound **2** should lie above its solubility, thus leading to aggregation, and self-assembly into a self-sorted network. In this way, by heating 14 mM of **1** and 2.5 mM of **2**, both at their respective MGC, and spontaneous cooling to 30 °C a hydrogel (*mix* **1 + 2**) is obtained. As can be seen in Figure 2A, the solubility profiles of both components in the mixture have not been significantly altered, which supports an extensive self-sorting process. In cryo-TEM (Figure 3D) long, flexible cylindrical fibers from gelator **1** are predominantly seen, as the major component, with a diameter of 11 ± 2 nm, together with few tubular fibers of similar thickness from gelator **2**, the minor component.

SAXS was used again to understand actual network architecture. However, it has been shown that even if the structure of single-component gels is known from SAXS, as is the case here, analyzing data from multi-component gels is difficult and ambiguous, particularly when one component is present in a significantly higher concentration.^43,44^ Thus, the SAXS data of hydrogel mixes are discussed rather qualitatively in the following. When examining the scattering data of *mix* **1 + 2** (Figure 4), the Porod scattering at high *q* can be interpreted as being dominated by the scattering contribution of flexible fibers formed by compound **1** which, due to its higher concentration, effectively masks the form factor oscillations of compound **2**. However, the low *q* scattering, which is proportional to *q* ^–2^ is likely to arise from the scattering by the ribbons formed by compound **2**. Most importantly, the upturn of the scattering intensity at even lower q indicates the segregation of the two types of fibers into larger structure or droplets. To obtain a characteristic length scale, the SAXS data were analyzed using a simple model of polydisperse aggregates, yielding an average thickness of 7 nm, a value somewhat smaller than the diameter of the fibers formed by compound **1**.

Indeed, as can be seen in Figure 5, The storage modulus *G*′ of the two-component hydrogel formed by the *mix* **1 + 2** is approximately 10 kPa, while *G*″ is one order of magnitude lower in each case, and all exhibit a largely frequency-independent plateau in the low to intermediate frequency regime, similar to the single-component Gelator **1** rather than any intermediate behavior expected for the mixture. However, the addition of Gelator **2** does result in a slight reduction in stiffness and brittleness compared to the single-component Gelator **1** network, indicated by the reduced *G*′ value of 5.9 kPa and yield strain of 0.5% determined in the amplitude sweep measurements (Figure S10D). The amplitude sweep also indicates two shoulders in the nonlinear region, suggesting the presence of two structurally different networks within the final composite material, which is likely to be the result of the independent assembly of each gelator component. Furthermore, the temperature-ramp experiment shows that hydrogel **2** melts precisely at its characteristic transition temperature (37 °C), whereas hydrogel **1** exhibits its entropy driven behavior,^42^ marked by an increase in *G*′ with rising temperature followed by melting at its *T*_gel_ (Figure S13). Thus, it could be observed that, over the whole temperature range, the mechanical properties of *mix* **1 + 2** hydrogel were dominated by the fibrillar network formed by compound **1**. Moreover, DSC confirmed that the two components aggregated independently and conserved their respective thermal transitions (Figure S14F).^27^

The second multicomponent hydrogel involves compounds **1** and **3** (*mix* **1 + 3**). In this system, we aim to evaluate whether the entropically driven hydrogel **1** can be independently self-assembled in the presence of gelator **3**, with a similar molecular scaffold but, as with compound **2**, exhibits an enthalpy-driven self-assembly profile. Moreover, it must be noted that compounds **1** and **3** bear complementary acid–base functional groups which may introduce additional charge screening interactions. The combined hydrogel is prepared like the previous one, using each component’s MGC (14 mM and 8.3 mM, respectively) and following a similar temperature gradient. However, in this case the solubility profile of compound **1** is clearly affected by the presence of compound **3**, in terms of concentration range as well as for the temperature response tendency which is now parallel to that of compound **3** (Figure 2B). Furthermore, DSC analysis demonstrates clear deviations from the thermal profiles of the single-component systems, including a broad endothermic melting event at ∼99 °C and a decomposition process above 190 °C (Figure S14D). Such features point to the emergence of a new phase with modified thermal characteristics. This interpretation is corroborated by temperature-ramp rheology, wherein the new system maintains superior mechanical robustness up to 95 °C, also reflected in its higher *G*′ and *G*″ values relative to the individual components (Figure S 11E).

Notably, the SAXS data (Figure 4) of this hydrogel *mix* **1+3**, which is the only two-component hydrogel that does not show an upturn at low *q*, also point towards a mutual co-assembly of the two gelators leading to the formation of a new network. Thus, the scattering behavior at low *q* scales exclusively with *q* ^–2^, indicating the presence of flat structures, a feature that is clearly different from the flexible, cylindrical fibers formed by the individual compounds **1** and **3**. A fit to a polydisperse model for flat structures revealed a mean lamellae thickness of 16 nm, while the persistence of the *q* ^–2^ scaling over the whole measured *q*-range suggests an aggregate width of at least 80 nm.

These results point to the co-assembly of the two gelators into a single type of fiber, together with a residual amount of compound **1** that self-sorts into few one-component fibers. To confirm the effectiveness of the co-assembly, an equimolar mixture of compounds **1** and **3**, both at 8.3 mM (MGC of **3**) was studied and their solubility profiles appeared almost superimposable (Figure S1) which suggests that they are probably forming dimers due to complementary intermolecular interactions, that exchange between solution and the gel fibers. To test this possibility, an^1^ H NMR titration of compound **1** with compound **3** at a total concentration of 4 mM was performed, and a 1:1 co-assembly stoichiometry was confirmed by a Job Plot analysis (Figure S2) following the chemical shift variation of aromatic protons in ortho to pyridine N of compound 1 and methylene protons in α to the carboxylate group of compound 3 caused by the electrostatic interactions between these two functionalities (see SI for details).The most direct evidence for the co-assembly of compounds 1 and 3 comes from the characterization data, particularly the diffusion coefficients observed by DOSY NMR (Figures S3, 5-6). The coincident diffusion behaviour shows that both components are moving as a single hydrodynamic entity, which is consistent with co-incorporation within shared fibrils rather than the coexistence of independent networks. It is noteworthy to mention that a remarkable reduction in solubility of compound **1** was observed, compared to its single-component system, supporting the formation of a co-assembled species with different solubility characteristics that is the building block for the formation of an alternate co-assembled network (Figure S1). Cryo-TEM reveals single kind of twisted cylindrical fibers with a width of 18 ± 2 nm (Figure 3E), almost double the thickness than observed for the individual gelators. Although the stiffness (*G*′) at this concentration has decreased comparatively to 11.6 kPa from 28.6 kPa (when both the components were at their respective MGC), the elasticity is sustained (∼0.07 tan­(δ)) (Figure S12).

The third dual-component hydrogel to be studied is formed by compounds **2** and **3** (*mix* **2 + 3**). In this case, both components show an enthalpy-driven self-assembly and a clearly different molecular scaffold. *Mix* **2+3** hydrogel at each component’s MGC (2:2.5 mM, 3:8.3 mM respectively) was formed, following the heating-cooling protocol and their solubility recorded at different temperatures by ^1^H NMR (Figure 2C). As can be seen, both components retain their solubility profiles when compared to the respective single-component hydrogels, pointing towards a self-sorted dual network. Two different morphologies are clearly visible through cryo-TEM: tubes from gelator **2** with ∼10 nm width and fibers from gelator **3** with the thickness of ∼14 nm similar to their individual morphology (Figure 3F). There are regions where we could only see flexible fibers from gelator **3** and domains with only tubes from gelator **2**, and very few regions with presence of both depicting microdomains formed in hydrogel (Figure S9). The formation of such microdomains is further supported by SAXS (Figure 4). Similar as *mix* **1 + 2**, at high q the scattering of hydrogel *mix* **2 + 3** is dominated by the contribution of flexible fibers formed by gelator **3** which, due to its higher concentration, effectively masks the form factor oscillations of the tubular fibers from gelator **2**, while the low *q* *q* ^–2^ -scattering likely originates from the flat ribbons of compound **2**. However, the upturn of the scattering intensity at lower *q* values is even more pronounced than in *mix* **1 + 2**, strongly indicating the formation of microdomains composed of self-sorted networks of the gelators **2** and **3**. Again, to obtain a characteristic length scale, the SAXS data were analyzed using the model of polydisperse flat aggregates resulting in a mean aggregate thickness of 7.3 nm, which is consistent with that determined for *mix* **1 + 2**. The two-component hydrogel *mix* **2 + 3** has a storage modulus G′, which is higher than that of single-component Gelator **2** but very close to the mechanical properties of single component gelator **3**, while still maintaining a well-defined gap between *G*′ and *G*″ over the entire range of frequencies (Figure 5F).

In contrast to the enhancement in mechanical properties observed in the *mix* **1 + 3**, no enhancement in mechanical properties is observed in the *mix* **2 + 3** combination; rather, the rheological properties are dominated by the stronger fibrillar network formed by gelator **3**, with minimal contribution to the overall elastic properties or the formation of a parallel network by gelator **2** (Figure S10F). This supposition is supported by temperature ramp measurements (Figure S11E), in which the *mix* **2+3** composite exhibits a thermally driven transition profile similar to that of gelator **3**, also seen by DSC with a glass transition at approximately 28 °C (Figure S14E), while the more typical melting transition for gelator **2** is only weakly resolved, as expected for a system of entangled yet largely independent fibers that coexist in the composite as a result of non-cooperative assembly. Collectively, these results indicate that *mix* **2 + 3** form a predominantly self-sorted, rather than co-assembled, material in which the mechanical properties are dominated by the gelator **3** network over the entire temperature range investigated.

## Conclusions

This study thus proves that solubility determination by NMR is a strategic tool for controlling molecular social behavior in multicomponent supramolecular hydrogels. In a broad and detailed study of the system, we show by a wide range of techniques, NMR, DSC, SAXS, TEM, and rheology, that both molecular structure and intermolecular interactions control not just the assembly of the system, but also the mechanical and thermal robustness of the final networks formed. For this purpose, we have designed challenging two-component mixtures to probe the feasibility of our analysis. Thus, of all the mixtures studied, *mix* **1 + 3** is found to be a true co-assembled network hydrogel, in which gelators 1 and 3 co-assemble within the same fibrils via a salt bridge between the pyridine unit of 1 and the carboxylic acid of 3, with synergistic enthalpic and entropic effects leading to a thermally robust system with enhanced properties, which are unattainable for each gelator individually. In contrast, *mix* **1 + 2** and **2 + 3** are found to be self-sorted systems, each forming their own individual networks, with a dramatic and unequivocal demonstration that even small differences in molecular geometry and thermodynamic preferences can strongly influence self-assembly behavior. Taken together (three gelators and three pairwise combinations), these results imply that multicomponent LMWG systems with complementary hydrogen bonding or ionizable functional groups can be considered as rationally designable materials rather than unpredictable mixtures, although validation for a more diverse range of gelator structures is required in order to establish this as a general design principle. By judiciously selecting molecular partners that possess distinctive solubility signatures, one can purposefully program co-assembly or self-sorting behavior— and hence desired mechanical, thermal, and functional properties. Looking to the future, this design strategy heralds promising prospects for the rational design of future generations of hydrogels for drug delivery, tissue engineering, and stimulus-responsive soft matter materials—a domain in which complexity, functionality, and precision must converge.

## Supplementary Material



## Data Availability

All data are available in the main text, in Supporting Information (SI) or upon request to the corresponding authors

## Notes

Materials description, experimental, synthesis and characterization of gelators, NMR spectra, rheology data, DSC data, SAXS data and fitting, microscopy images (PDF)

## Abbreviations

LMWG: low molecular weight gelator
NMR: nuclear magnetic resonance
SAXS: small-angle X-ray scattering
DSC: differential scanning calorimetry
cryo-TEM: cryo-transmission electron microscopy
