# Supplementary material for: Solubility-Driven Design of Multicomponent Self-Assembled Hydrogels
Source: Langmuir. 2026 Sep 17;42(38):28201–9. doi: 10.1021/acs.langmuir.6c03536 (PMC13629671; doi:10.1021/acs.langmuir.6c03536)
Supplement: Supplementary file 1 [file la6c03536_si_001.pdf]

Supporting information

Solubility-Driven Design of Multicomponent Self-Assembled Hydrogels

Misthi Singh[a], Florian Trummer[b], Peter Schmiedel*[c], Thomas Sottmann*[b], Beatriu Escuder [a]*

[a]Institute of Advanced Materials (INAM), Universitat Jaume I, 12071 Castelló, Spain.

[b]Institute of Physical Chemistry, University of Stuttgart, Pfaffenwaldring 55, 70569 Stuttgart, Germany.

[c]Research & Open Innovation-Material Science, Henkel AG & Co. KGaA, 40589, Dusseldorf, Germany

*Corresponding author: Email: Beatriu Escuder: escuder@uji.es, Thomas Sottmann: thomas.sottmann@ipc.uni-stuttgart.de, Peter Schmiedel: pseter.schmiedel@henkel.com

Table of contents

1. General Experimental Methods S4
2. Synthesis procedure of gelators S6
3. Characterization of Gelators (^{1}H-NMR, ^{13}C-NMR, Mass, FTIR) S8
4. Hydrogel Characterization S16
5. Solubility Studies S17
6. Hydrogel formation of dual component S23
7. Solubility Studies of dual component S24
8. NMR Titration of Gelator 1 and 3 S27
9. Diffusion-Ordered Spectroscopy S30
10. Microscopy S33
11. Rheology S40
12. Differential Scanning Calorimetry S43
13. Small-Angle X-ray Scattering S46

Index of Supporting Figures cited in the Main Text

Figure S1: Solubility profile of mix 1+3 at MGC of 3 (8.3mM each) S25

Figure S 2: Jobs plot analysed from 1HNMR Titration of 1 and 3 S29

Figure S 3: DOSY spectra of compound 1 S30

Figure S 4: DOSY spectra of compound 2 S30

Figure S 5: DOSY spectra of compound 3 S31

Figure S 6: DOSY spectra of compound mix (1+3) S31

Figure S 7: DOSY spectra of compound mix (2+3) S31

Figure S 8: DOSY spectra of compound mix (1+2) S32

Figure S 9: Cryo-TEM image of mix 2+3 at minimum gelation concentration S39

Figure S 10: Amplitude sweeps for one-component and two-component hydrogels S40

Figure S 11: Temperature ramp for single component hydrogel and dual mixture component hydrogel S41

Figure S 12: mix 1 + 3 at minimum gelation concentration of Gelator-3 (8.3mM each) S41

Figure S 13: The comparative temperature ramp of gelator 1, 2 and mix 1+2. S42

Figure S 14: DSC thermograms for one-component and two-component hydrogels S44

1) General Experimental Methods.

Nuclear magnetic resonance spectroscopy studies

NMR spectra were recorded at 400 MHz ^{1}H NMR and ^{13}C NMR in a Bruker Ascend™ 400 NMR spectrometer in different deuterated solvents at 30°C with the solvent signals as an internal reference. DOSY NMR was recorded at 500 MHz Varian INOVA NMR. The instrument is configured with the Nalorac NTR probe, optimized for ^{1}H sensitivity. For all the samples, the lowest gradient value is 800, the highest gradient is 25000, and the diffusion gradient length is 6ms.
For kinetics, freshly prepared hydrogel was directly transferred to co-axial NMR tube for further experiment. For solubility studies, NMR tubes were made with Hydroquinone inside the co-axial tube for internal standard and than kept for 10 hours at room temperature to age before further experiment.

Mass spectrometry

The mass spectra were measured with a triple-quadrupole mass spectrometer with an electrospray source (water). A capillary voltage of 3.5 kV was used in positive scan mode. The observed isotopic pattern of each compound perfectly matched the theoretical isotope pattern calculated from their elemental composition using the MassLynx 4.0 program.

Fourier Transform Infrared Spectroscopy (FT-IR).

Spectra were recorded for solid samples using a JASCO FTIR/6200 spectrometer equipped with an ATR (MIRacle single-reflection ATR diamond/ZnSe) accessory at 4 cm^{-1} (4000–600 cm^{-1} spectral angle).

Transmission electron microscopy

Samples were applied directly onto Carbon support film on 200 mesh copper grids. Excess amount of sample was drained gently and washed with 5 μL of water once and the residue of water removed. Transmission electron microscopy (TEM) images were recorded on a JEM 2100plus Transmission Electron Microscope.

Cryo Transmission Electron Microscopy

(Cryo-TEM) in a TECNAI T20 SuperTwin (FEI-Thermofisher), operating at an accelerating voltage of 200 KV in a bright-field image mode. Samples preparation of the cryo-TEM involved a vitrification procedure on a FEI Vitrobot Mark IV (Eindhoven, The Netherlands). One drop of the sample solution (~3 μL) was deposited in a copper grid (300 mesh Quantifolis, hydrophilized by glow-discharged treatment just prior to use) within the environmental chamber of the Vitrobot and the excess liquid was blotted away. The sample was shot into melting (liquid) ethane and transferred through 655 Turbo Pumping Station (Gatan, France) to a 626 DH Single Tilt Cryo-Holder (Gatan, France), where it was maintained bellow - 170 °C (liquid nitrogen temperature). The sample was examined in the TECNAI G2 20 TWIN (FEI) mentioned above, operating at an accelerating voltage of 200 kV. TEM images were acquired with an Eagle 2K x 2K CCD camera.

Rheology

Rheological measurements were conducted on a Discovery HR-30 rheometer from TA instruments using a steel parallel plate-to-plate geometry (40 mm diameter). The gap distance was fixed at 500 μm. The hydrogels were prepared under the desired conditions and the hot solution of samples transferred to the rheometer plate, well-covered with the instrumental metal cover to avoid evaporation and stabilised for 5 minutes before analysing. Viscoelastic characteristics were studied under oscillatory experiments. Frequency and amplitude sweeps were examined at 25°C. The experimental conditions were arranged by running an amplitude sweep (0.001-100% strain at 1 Hz) and a frequency sweep step (0.001–100 Hz).

DSC analysis

DSC analyses were carried out with a heating rate of 10 K/min with a mass of 2-3 mg of gelators and flushing gas N_2 (3 L/h) in measuring crucible Tzero-crucible (Al) 6, encapsulated with DSC X3, TA Instrument.

Small-Angle X-ray Scattering (SAXS) – Experimental

Small-angle X-ray scattering (SAXS) measurements were conducted on the Xeuss 3.0 UHR beamline (Xenocs SAS, Grenoble, France) at the University of Stuttgart. The heated gel precursor solutions were loaded into mark tubes with a path length of 1.98 mm (Hilgenberg GmbH, Malsfeld, Germany), sealed and left to gel at room temperature. The samples were illuminated with a rectangular beam (500x500 µm^2) with a wavelength λ = 1.5406 Å (Cu-Kα). The scattered radiation was detected by a Dectris EIGER2® Si 1M detector (Dectris AG, Baden-Daettwil, Switzerland) at four sample-detector distances (150, 500, 1500 & 4500 mm), resulting in an accessible q-range of 0.004 – 3.5 Å^{-1}, where $q = \frac{4\pi}{\lambda}\sin\left(\frac{\theta}{2}\right)$ denotes the absolute value of the scattering vector that depends on the wavelength of the primary beam and the total scattering angle θ. The calibration to absolute intensities was performed by direct beam transmission measurement and the data reduction was performed with the software XSACT™ (v2.10). All samples were measured at a temperature of 25°C.

Small-Angle X-ray Scattering (SAXS) – Theory

In a SAXS measurement, the q-dependence of the scattered intensity gives access to the nanostructure of the investigated sample on a length scale of ca. 1-500 Å. The scattered intensity $I(q)$ in a dilute sample can be expressed as $I(q) \propto P(q) = \int_{-\infty}^{\infty} \rho(\boldsymbol{r}) \exp(i\boldsymbol{qr}) dV$, with the electron density distribution $\rho(\boldsymbol{r})$ of the scattering objects at each position $\boldsymbol{r}$. The form factor $P(q)$ thus contains rich information about the morphology and structure of the sample. The analysis of the scattering curve may be, e.g., performed by fitting appropriate model expressions to the scattering data or by the application of indirect Fourier transformation methods that allow to retrieve the real-space structure directly from the scattering curve without any constricting model assumptions.

2) Synthesis and Characterization of compounds

All solvents were used as HPLC quality. THF was dried with metallic sodium and distilled. All chemicals were commercially available products with purity higher than 98%. All compounds synthesized were dried in vacuum oven at 60º to constant weight. Thin layer chromatography analyses were carried out on Alugram® Silica 9/UV254, and the compounds were visualized with ninhydrin or UV Light. Most of the reactions were carried out under N_2 atmosphere. ^{1}H NMR and ^{13}C NMR spectra were recorded on a Varian Mercury Vx 400 MHz and Varian Unity INOVA 300 MHz

.

Synthesis of N,N'-((2S,2'S)-(propane-1,3-diylbis(azanediyl))bis(3-methyl-1-oxobutane-1,2 diyl))diisonicotinamide {Gelator 1}

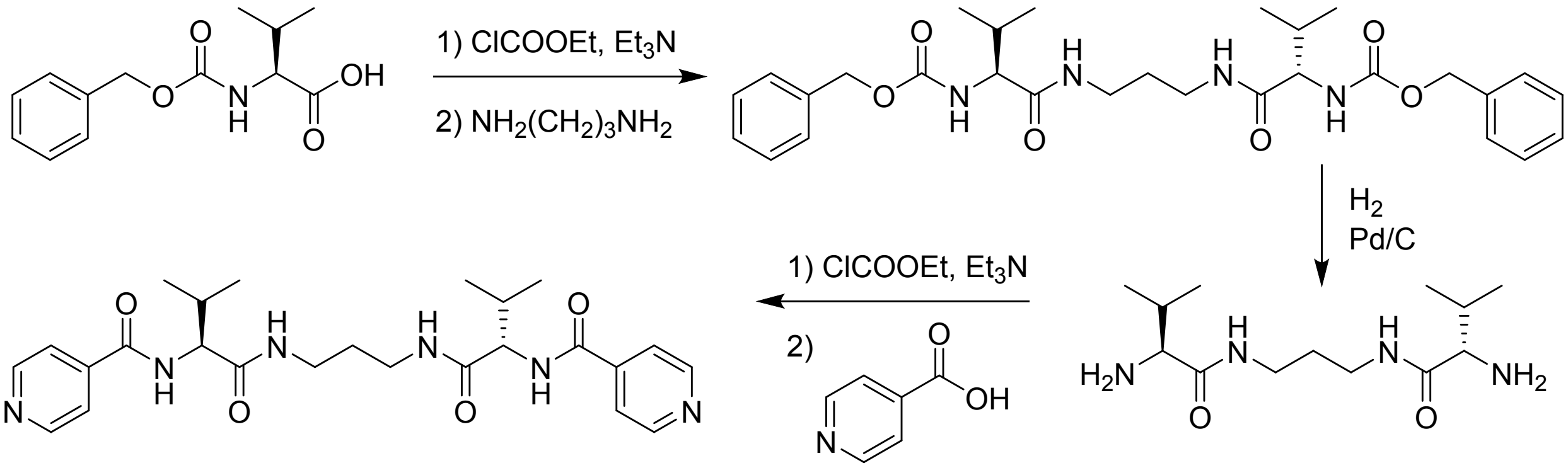

To a solution of the corresponding N-Cbz protected L-amino acid (20 mmol) and triethylamine (20 mmol) in THF (150 mL), ice cooled ethyl chloroformate (20 mmol) was added drop wise with vigorous stirring. Then the pasty reaction mixture was stirred for 30 min in an ice bath (temperature 5–10°C). To this mixture a solution of the corresponding diamine (9 mmol) in THF was added dropwise for 15 min, the system was stirred at 0°C for 1 h and allowed to warm up to room temperature with vigorous stirring overnight. The resulting white solid was filtered off and washed sequentially with a cold solution of 1M NaOH, water and cold methanol. Finally, the solid was dried in vacuum oven at 60ºC for 24h to obtain the Cbz-protected product (precursor of compound **1**; 4.30 g, 7.76 mmol, 86%). Cleavage of Cbz-protecting group was achieved via hydrogenolysis. The corresponding N, N´-Bis-(N-Cbz-L-aminoacyl) diamine (precursor of compound 1; 7.76 mmol) and a catalytic amount of Pd on activated carbon (510% w/w.) were placed in a two necked round bottom flask and suspended in MeOH (120 mL). The system was purged twice and connected to H_2 atmosphere. The pasty grey suspension was stirred for several hours until the reaction was complete according to TLC ($MeOH:CH_2Cl_2$ 1:1; ninhydrin). The black suspension was filtered over celite, the solvent was evaporated under reduced pressure and the corresponding N, N´-Bis(L-aminoacyl) diamines, obtained as oils, were dried further in vacuum pump for 24h. (precursor of compound **1**; 2.01 g, 7.41 mmol, 95 %). In the final step, to an ice cooled solution of isonicotinic acid (20 mmol) and triethylamine (40 mmol) in THF (120 mL), ethyl chloroformate (20 mmol) was added dropwise with vigorous stirring. Then the pasty reaction mixture was stirred for 30 min in ice bath (temperature 5–10°C). To this mixture a solution/suspension of the corresponding N, N´-Bis-(L-aminoacyl) diamines (6 mmol) in THF was added dropwise for 15 min and the mixture was stirred at 0°C for 1h and allowed to warm up to room temperature with vigorous stirring overnight. The resulting white solid was filtered

and washed with NaOH 1M. Then the remaining triethylamine was evaporated in vacuum pump for 24h. Afterwards, the product was further washed sequentially with cold water and cold acetonitrile and finally dried in vacuum oven at 60ºC for 24h. (precursor of compound 1; 2.29 g, 4.74 mmol, 79%).

Synthesis of 1,1'-(methylenebis(4,1-phenylene))bis(3-((R)-2,3-dihydroxypropyl)urea) {Gelator 2}

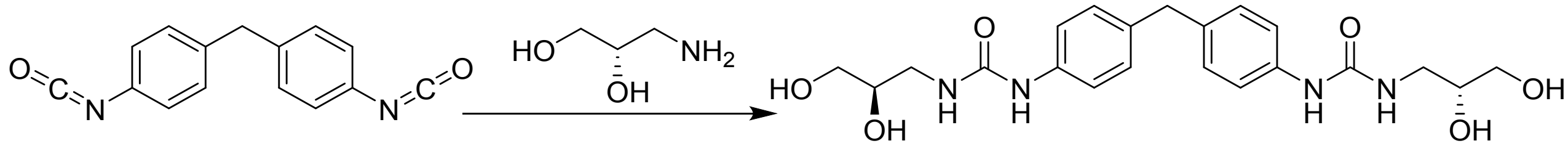

1-Isocyanato-4-(4-isocyanatobenzyl) benzene (4,4'-MDI) (1 g, 3.9 mmol) was dissolved in 40 mL of dry acetonitrile and was added dropwise to a solution of R-3-amino-1,2-propanediol (8.6 mmol) in 40 mL of dry acetonitrile at 50 ºC under argon atmosphere. The mixture was stirred overnight and afterwards the solution was concentrated in vacuo. The crude reaction mixture was suspended in water, refluxed for 2 h and filtered hot. After water removal and exhaustive drying a white solid was obtained with 93%yield.

Synthesis of (5S,13S)-13-(((benzyloxy)carbonyl)amino)-5-(2-carboxyethyl)-3,6,12-trioxo-1-phenyl-2-oxa-4,7,11-triazahexadecan-16-oic acid {Gelator 3}

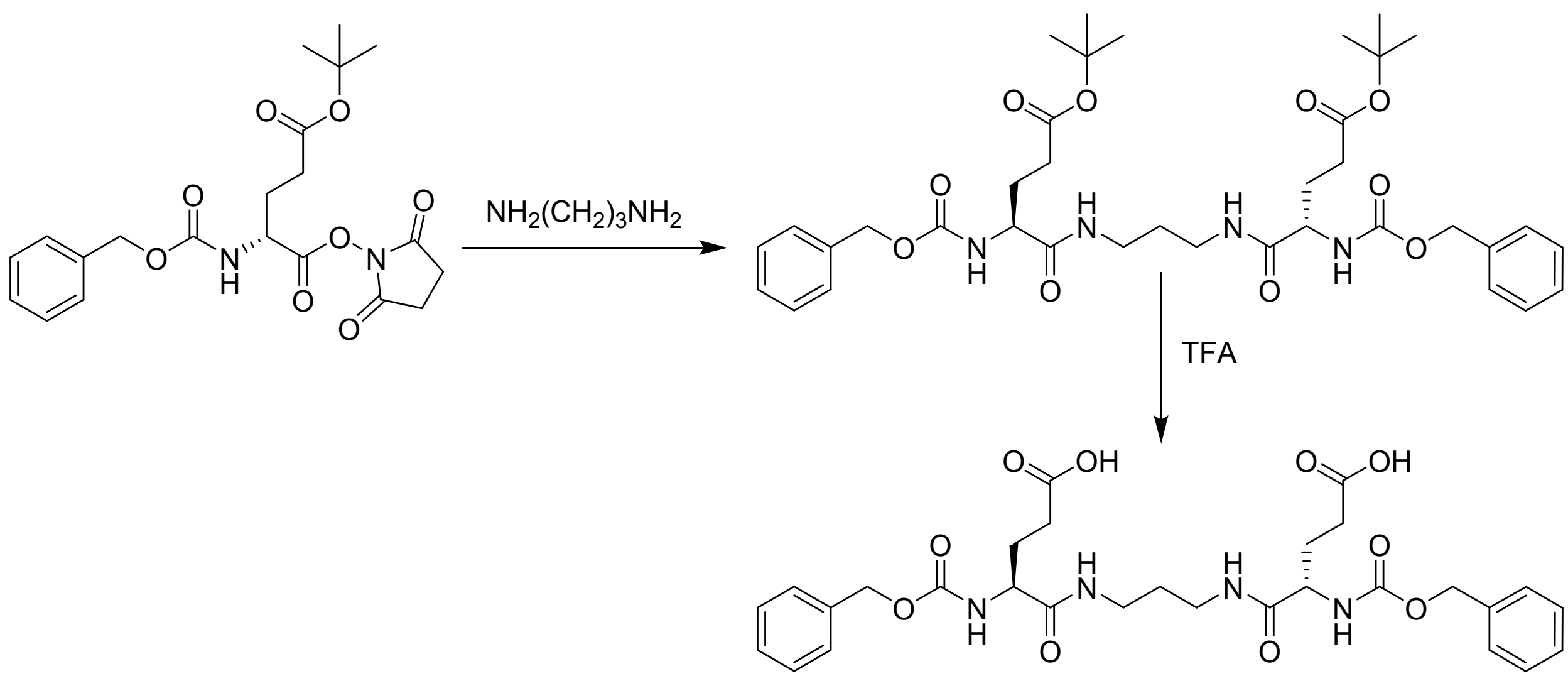

Activated 2.02g (4.6mmol, 2eq) of Z-Glu(OtBu)-Osu in DME was added dropwise to 1,3-diaminopropane (2.3mol, 1eq) under inert atmosphere. After 24hrs the product is dried under reduced pressure. Obtained solid was washed with 0.1M HCl, 0.1M and distilled water. Z-(Boc)Glu-3 was dissolved again in DCM and 5ml of TFA was added dropwise for tert-butyl deprotection. The mixture is dried under reduced pressure and obtained an orange oil which was suspended in water and NaOH pellet were added little by little to increase the pH which resulted in precipitation. Solid is filtered out on frite plate and washed with distilled water until the pH is neutral. Product is taken and left to dry with 97%yield.

3) Characterization of synthesized compounds

Gelator 1:

^{1}H NMR (400 MHz, DMSO-d6) δ = 8.704 (d, J = 8Hz, 4H); 8.628 (d, J=11.6Hz, 2H); 8.102 (t, J = 7.2, 2H); 7.784 (d, J= 8Hz, 4H); 4.224 (t, J=10.8Hz, 2H); 3.184-3.096 (m, 2H); 3.096-3.002 (m, 2H); 2.139-2.044 (m, 2H); 1.606-1.513 (m, 2H); 0.912 (d, J=9.2Hz, 6H); 0.897 (d, J=8.8Hz, 6H). 13C NMR (101 MHz, DMSO-d6) δ (ppm): δ 170.58, 165.03, 150.05, 141.22, 121.59, 59.30, 36.38, 29.92, 29.05, 19.27, 18.87. HRMS (ESITOF): [M+H]+=483.2720; [M+H]+=483.2723

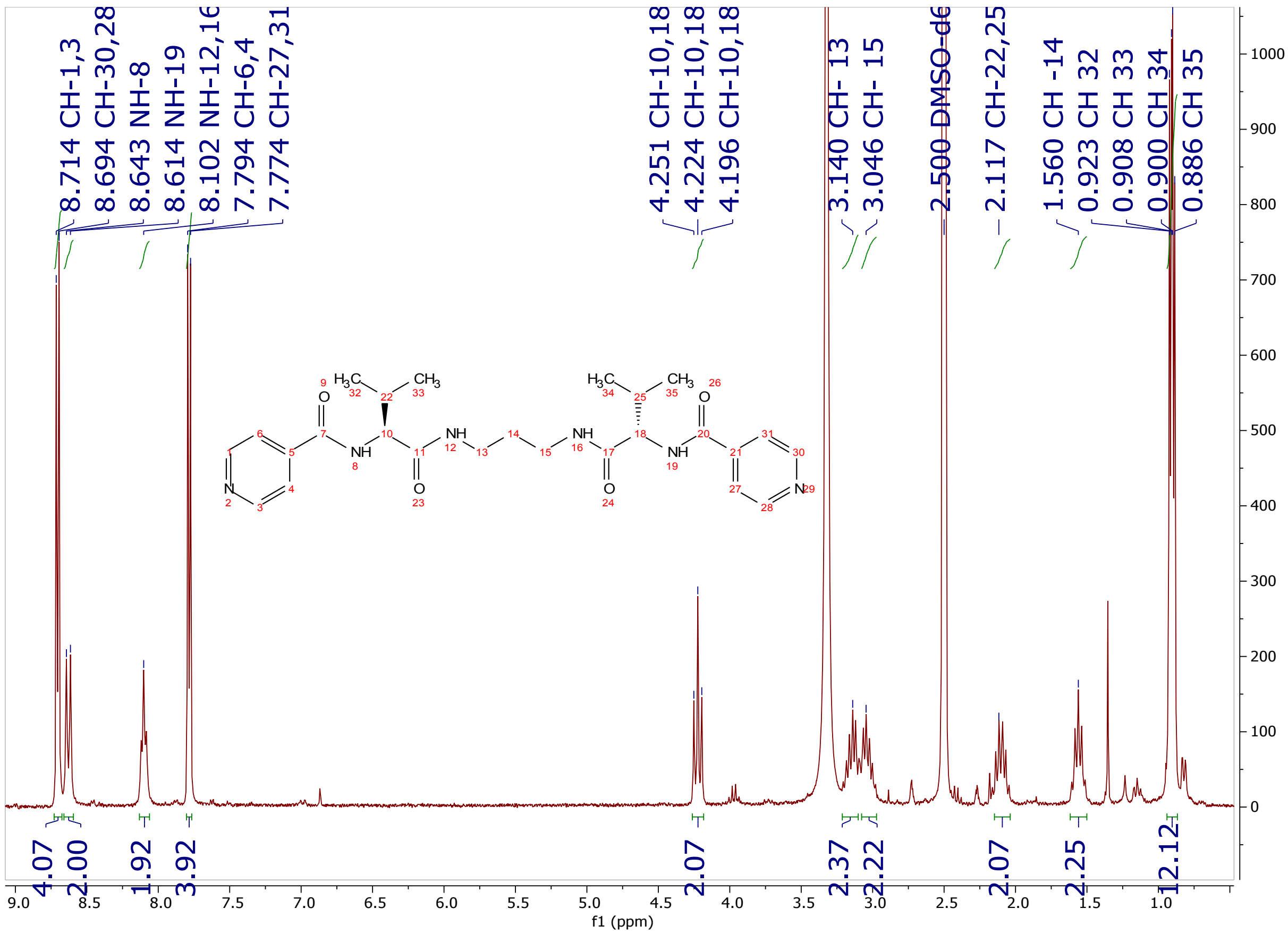

^{1}H-NMR 400MHz spectrum of Gelator **1**, reference solvent DMSO-d6 (2.5ppm).

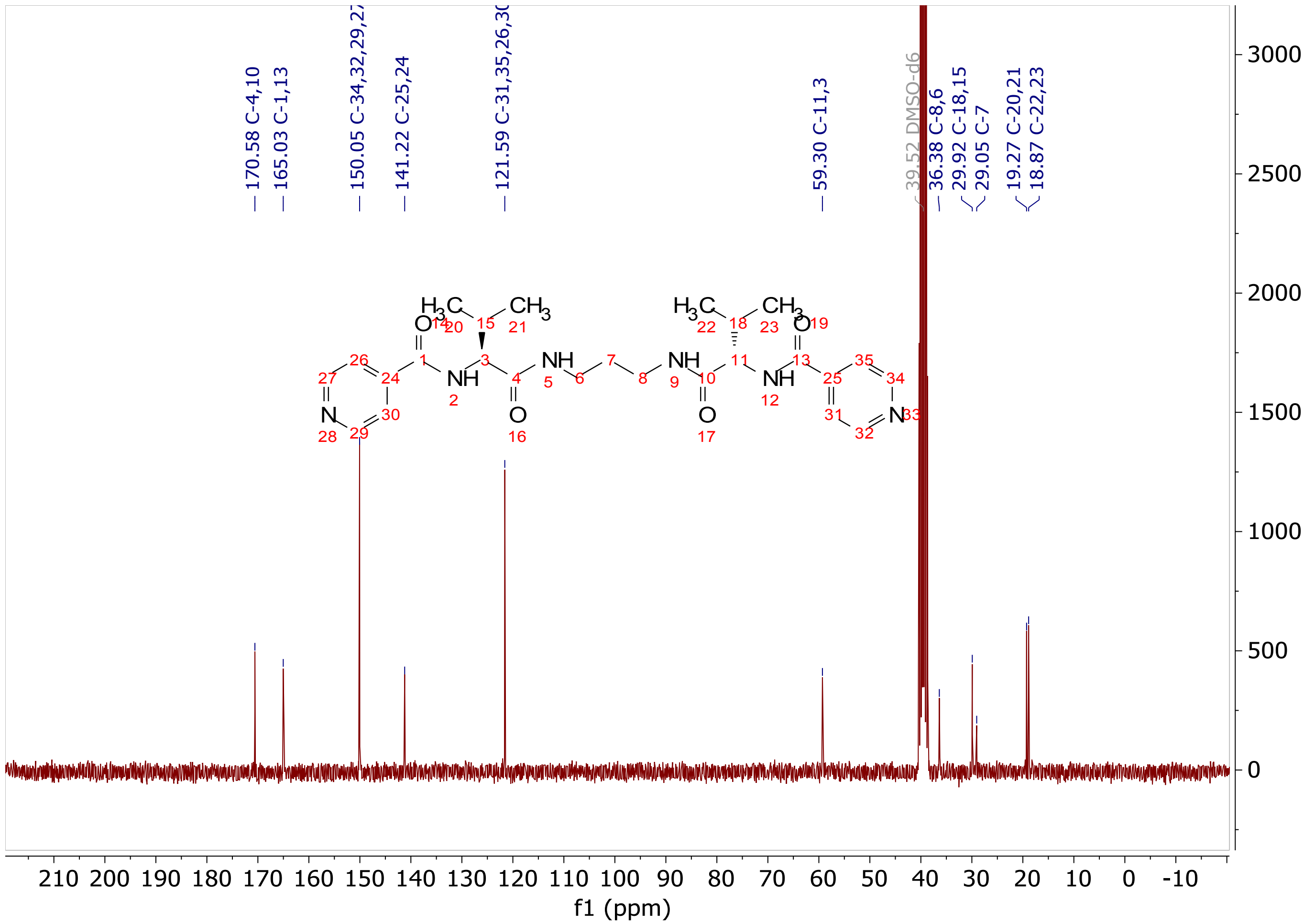

^{13}-C NMR spectrum of Gelator **1**, reference solvent: DMSO d6 (2.5ppm).

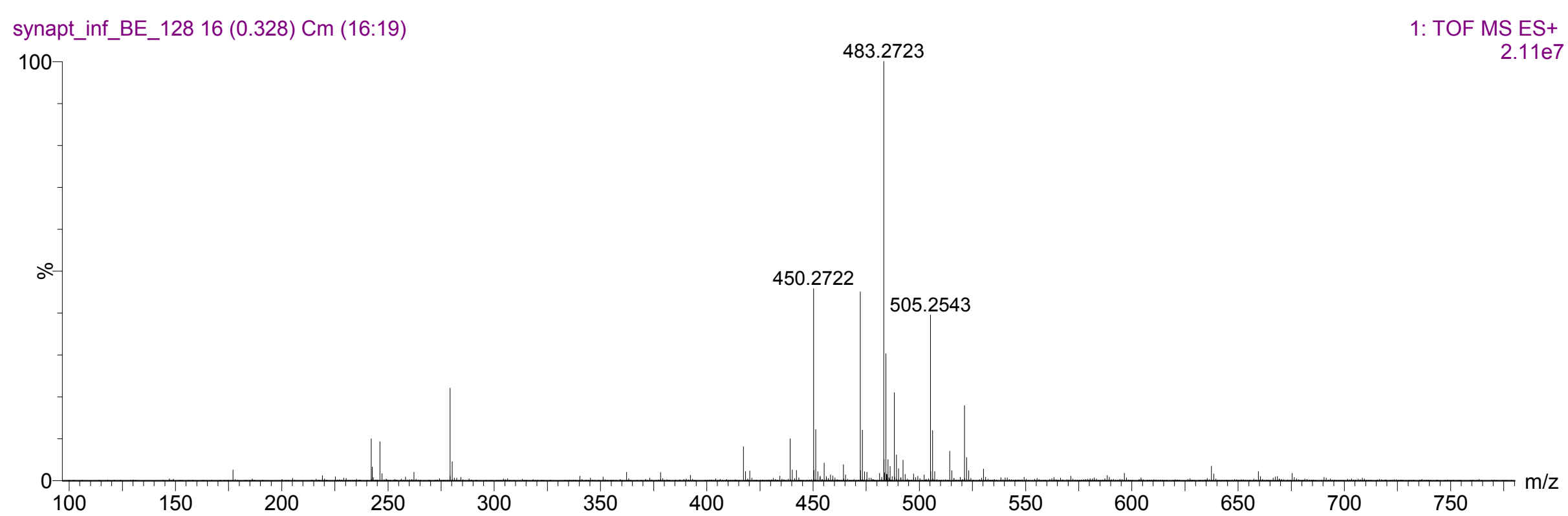

Mass spectra of Gelator **1**.

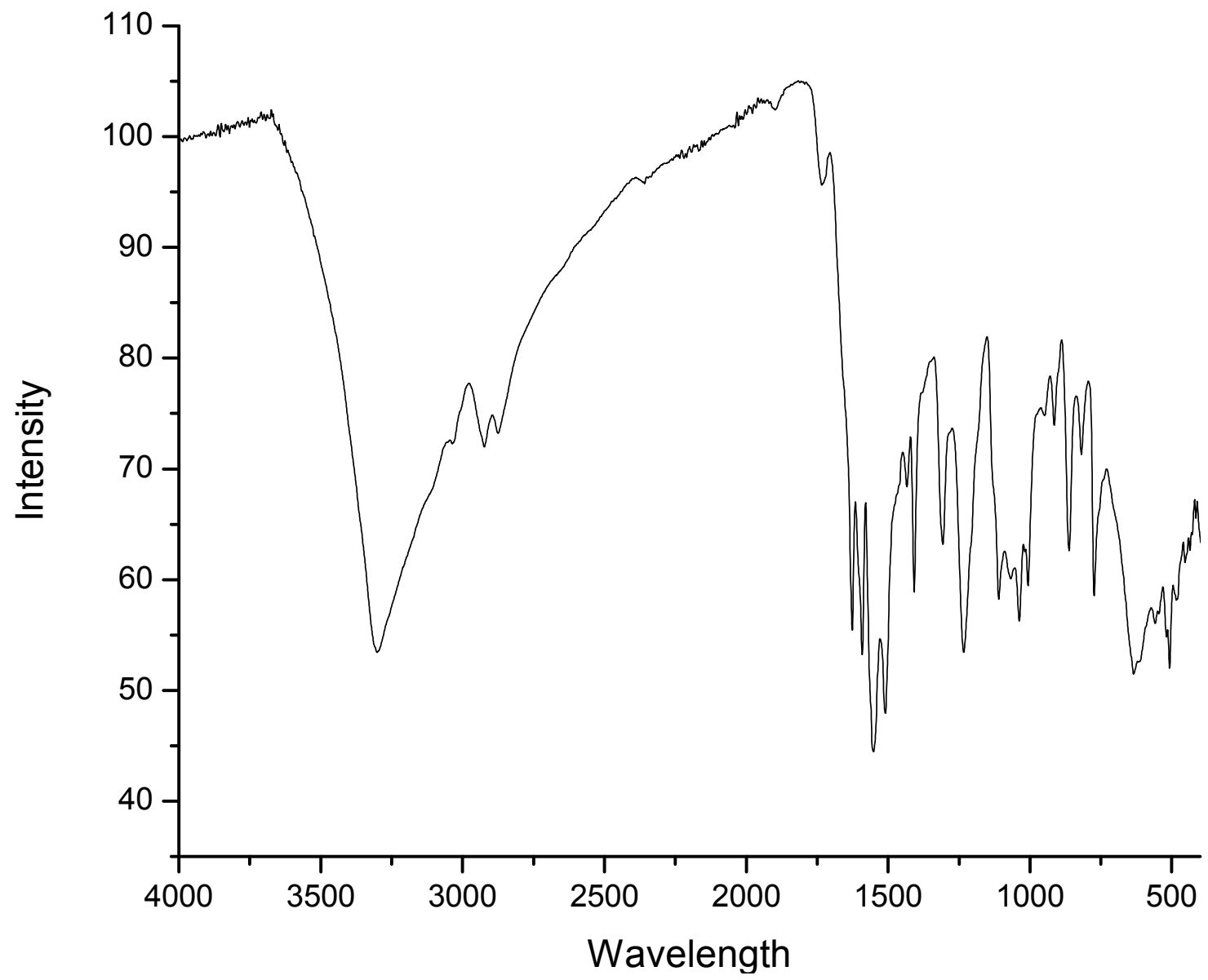

FTIR analysis of Gelator **1**.

Functional group	Peak	Notes
**N–H stretch (amide)**	3282	Slightly sharper than compound a (less O–H interference)
**C=O stretch (amide I)**	1634	Strong
**N–H bend + C–N stretch (amide II)**	1544–1596	Medium
**Pyridine ring C=C stretch**	1510	Medium
**Pyridine ring out-of-plane bend**	772	Sharp, diagnostic
**C–H stretch (aliphatic CH_2)**	2872–2960	Weak
**pyridine C–N stretch**	1236	Sharp & strong
**C–N stretch + N–H bend**	1311	Medium
**CH_2 scissoring**	1409	Sharp & medium

Gelator 2:

^{1}H NMR (400 MHz, DMSO-d6) δ = 8.419 (s,2H),7.257 (d, J = 11.2Hz, 4H), 7.0265 (d, J = 11.6 Hz, 4H), 6.075 (t, J = 6.4Hz, 2H); 4.819 (d, J = 6.4Hz, 2H); 4.566(t, J=7.6Hz , 2H); 3.742(s, 2H); 3.527-3.435 (m,2H); 3.373-3.337(m, 2H); 3.287-3.230 (m, 4H); 2.994-2.910 (m, 2H). ^{13}C NMR (101 MHz, DMSO-d6): δ 155.49, 138.39, 134.15, 128.73, 117.67, 70.67, 63.69, 42.45, 40.33. HRMS (ESITOF): [M+Na]+=455.1907; [M+Na]+=455.1914

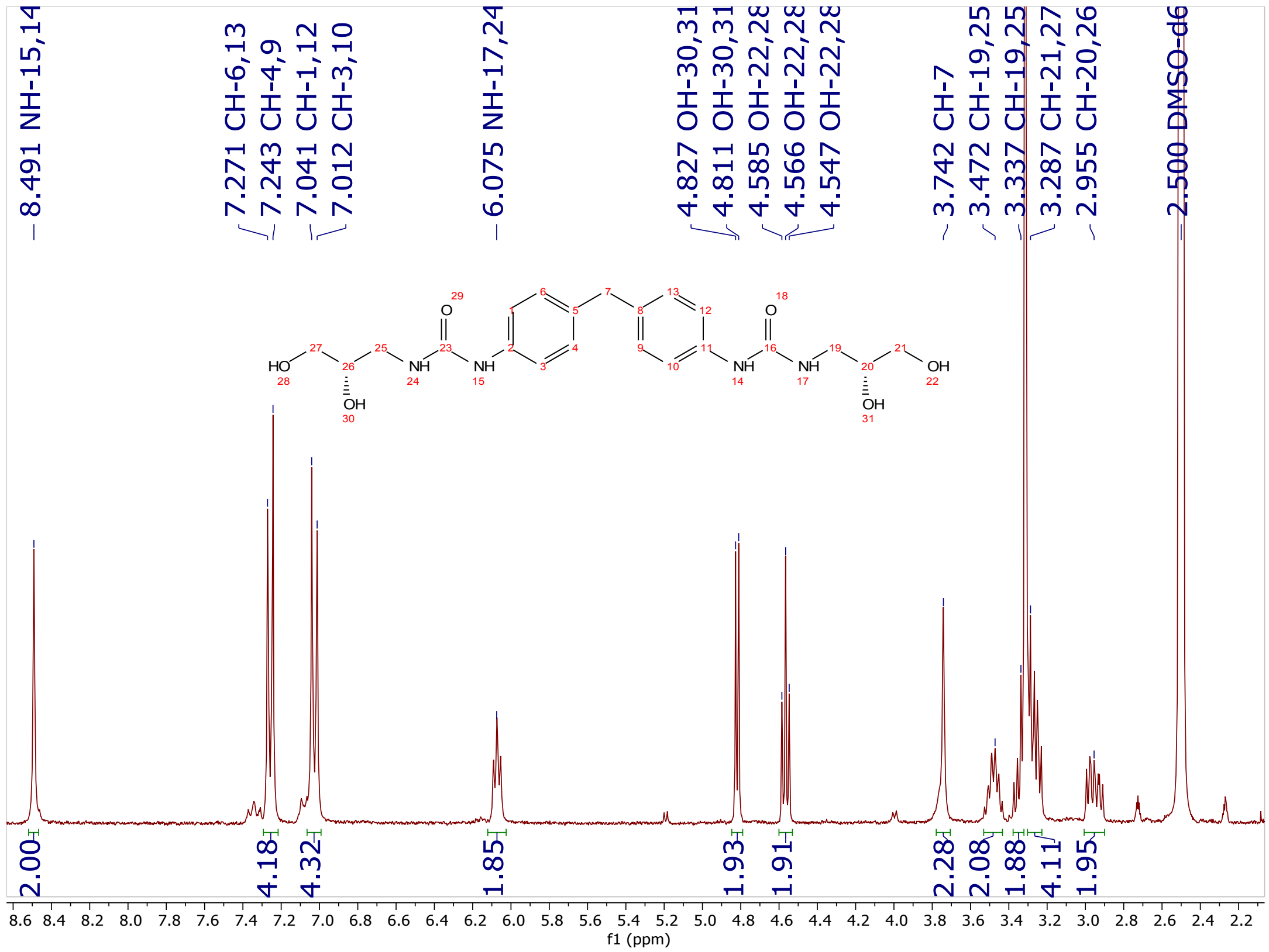

^{1}H NMR spectra of Gelator **2**, reference solvent: DMSO d6 (2.5ppm).

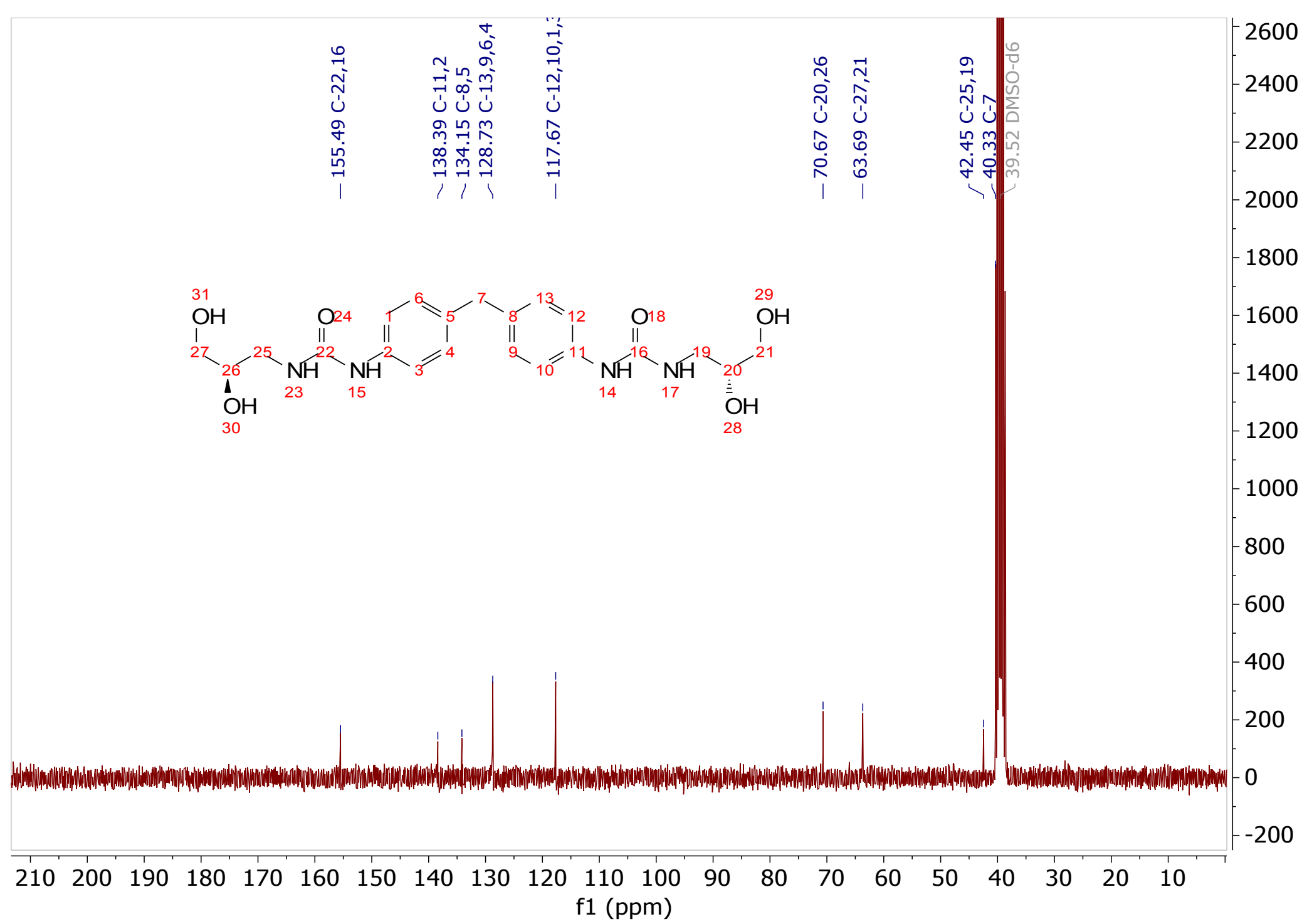

^{13}C NMR spectra of Gelator **2**, reference solvent: DMSO d6 (2.5ppm).

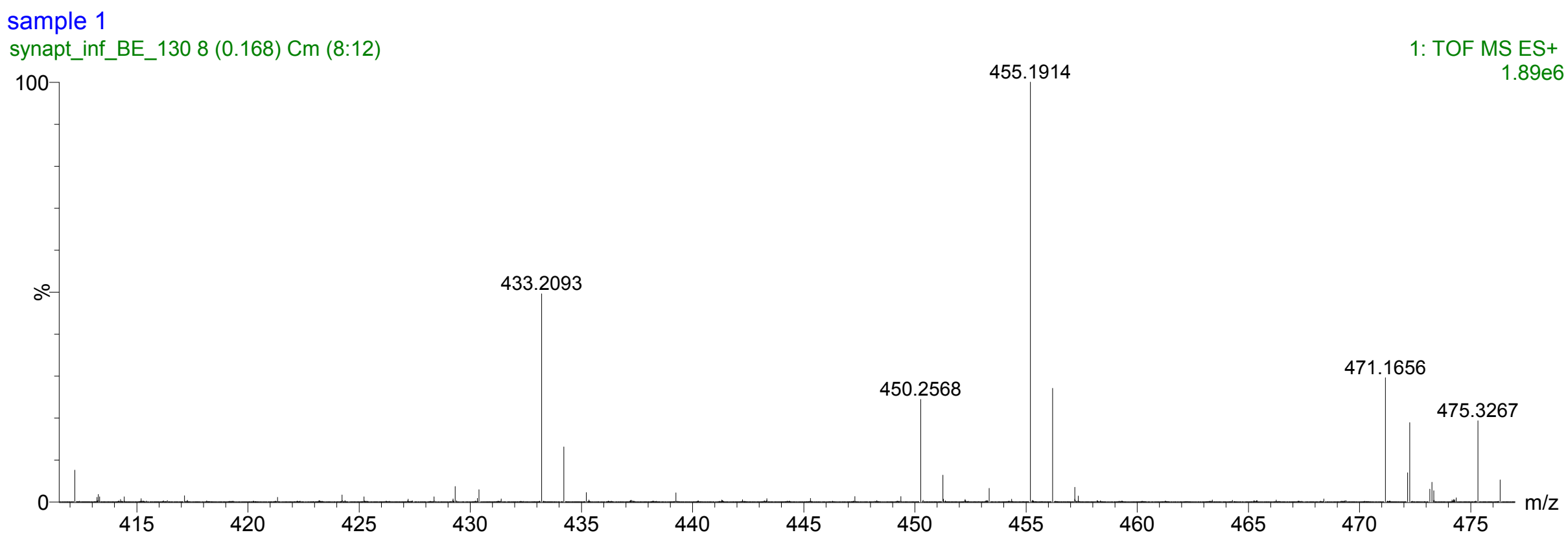

Mass Spectra of Gelator **2.**

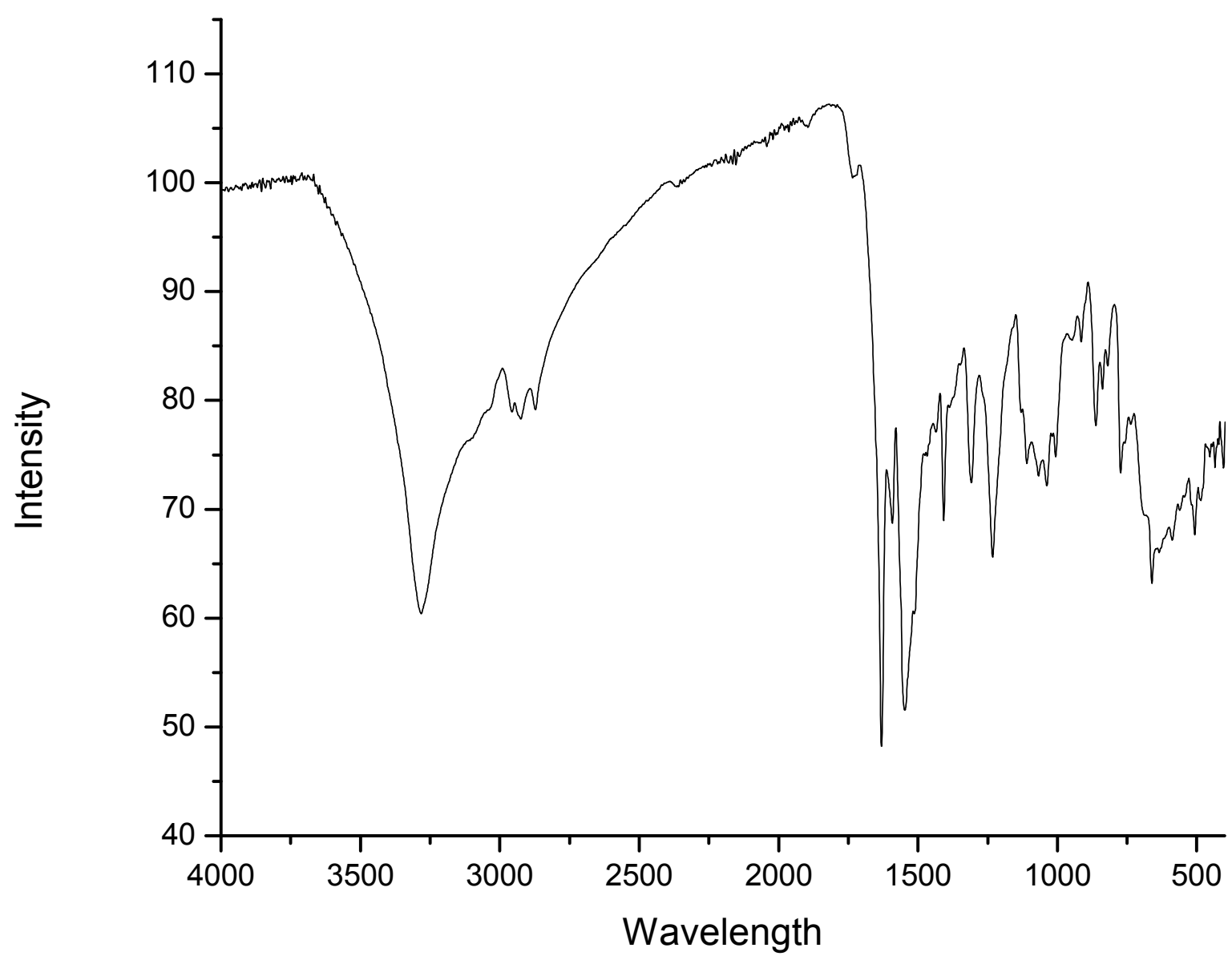

FTIR analysis of Gelator **2**.

Functional group	Peaks	Notes
**O–H stretch (alcohol)**	3302(broad)	Strong, broad
**C=O stretch**	1734	Sharp, strong
**C–O stretch (C–O–C)**	1310	Medium, split peaks possible
**C–H stretch**	2919	Weak-medium
**Aromatic C=C stretch**	1625-1509	Medium
**C–H stretch (aliphatic CH_2)**	2868	Weak
**Aromatic C–H scissoring (benzyl groups)**	1433	Medium
**H-bonded O–H in-plane bending**	1409	Medium

Gelator 3:

^{1}H NMR (400 MHz, DMSO-d6) δ = 7.887 (t, J = 7.2 Hz, 2H), 7.418 (d, J= 10.8Hz, 2H), 7.364-7.297 (m, 10H), 5.024 (s, 2H), 5.013 (s, 2H), 3.973-3.901 (m, 2H), 3.05 (m, 2H), 2.237(t, J = 10.8Hz, 4H), 1.921 –1.852 (m, 2H), 1.783-1.682 (m, 2H), 1.534-1.476 (m, 2H). ^{13}C NMR (101 MHz, DMSO-d6): δ 173.84, 171.36, 155.95, 136.98, 128.31, 127.77, 127.70, 65.45, 54.17, 36.10, 30.24, 29.13, 27.18. HRMS (ESI TOF): [M+H]+= 601.2512; [M+Na]+=623.2331

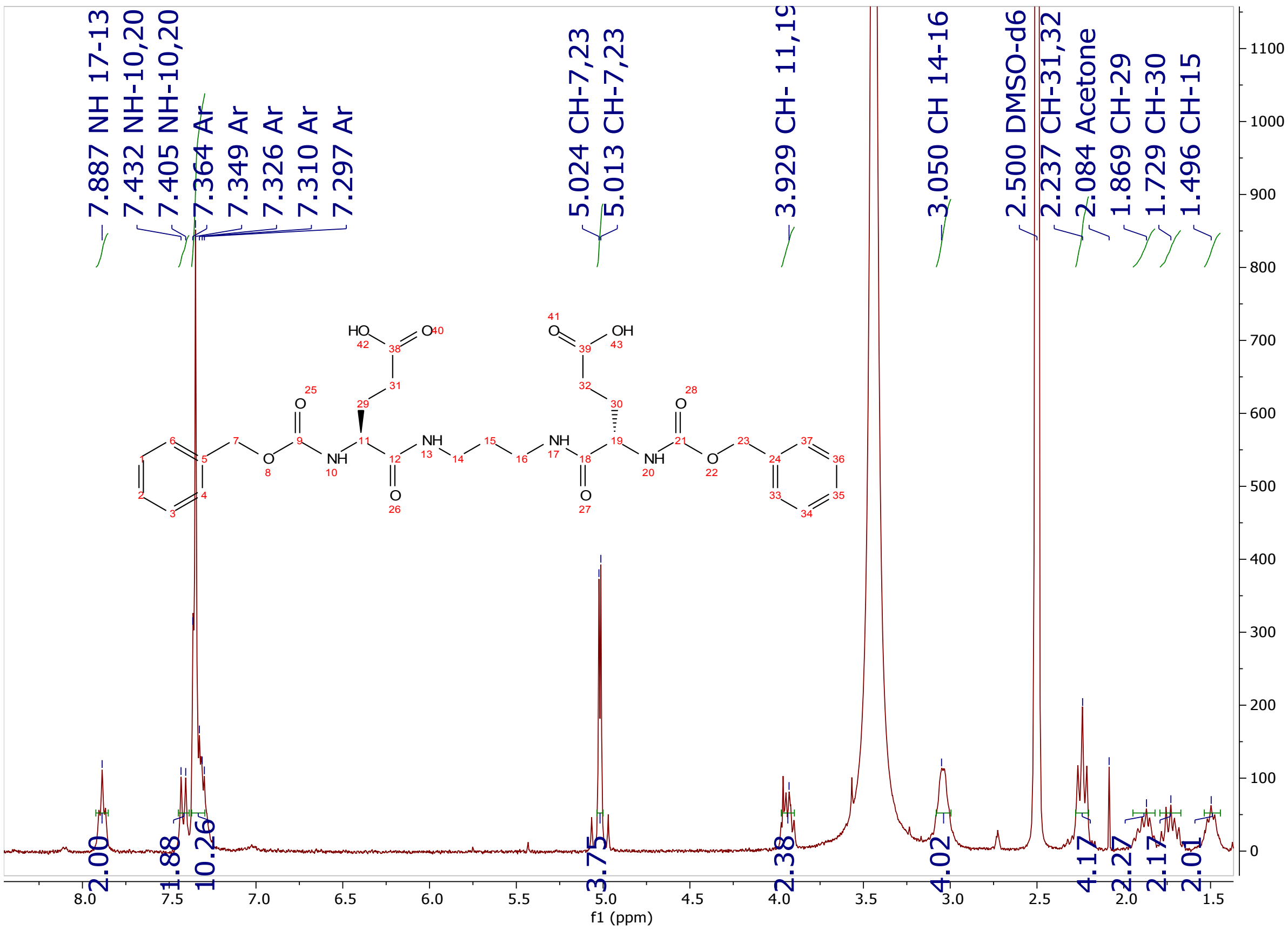

^{1}H NMR 400MHz spectra of Gelator **3**, reference solvent: DMSO d6 (2.5ppm).

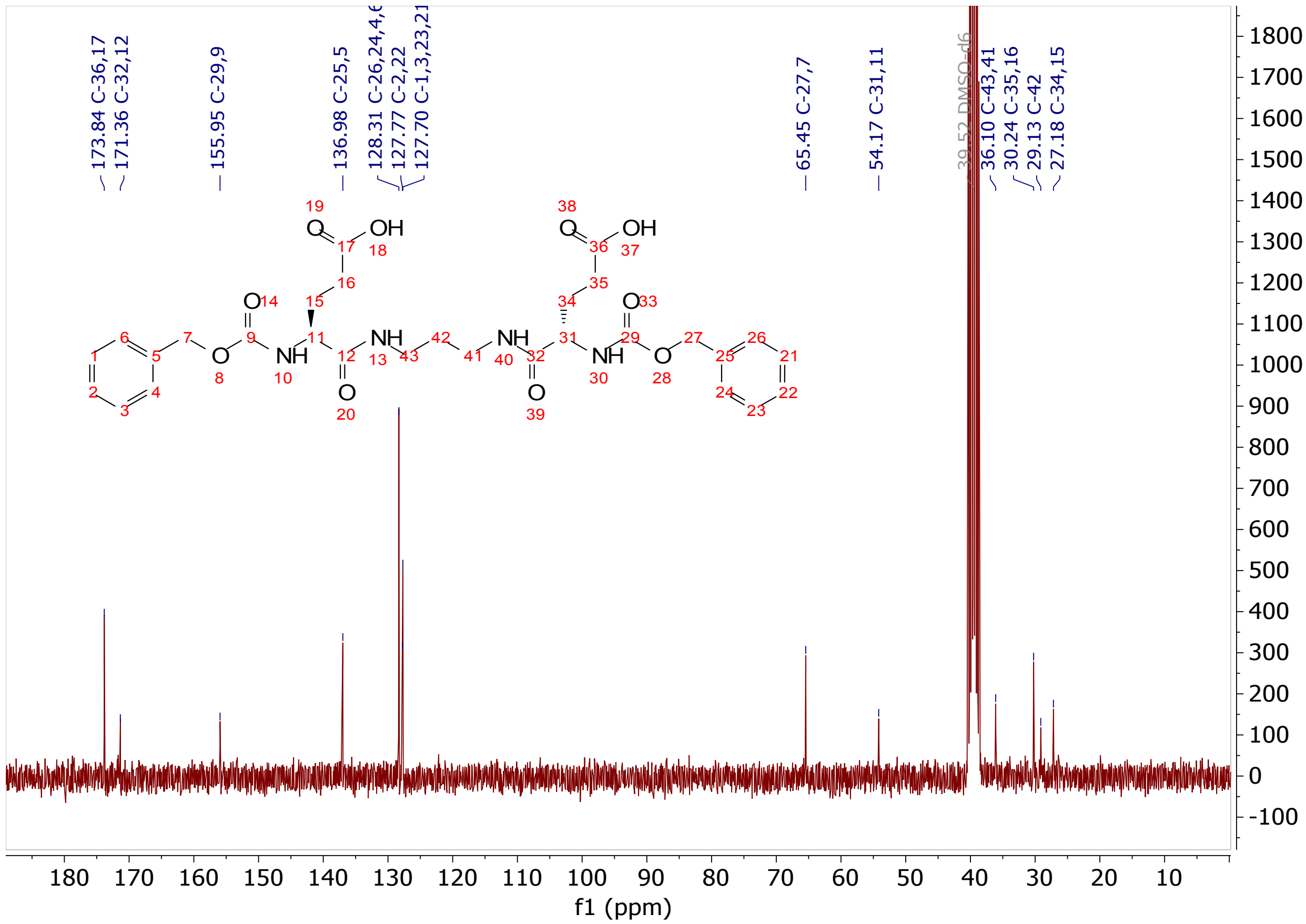

^{13}C NMR Spectra of Gelator **3**, reference solvent: DMSO d6 (2.5ppm).

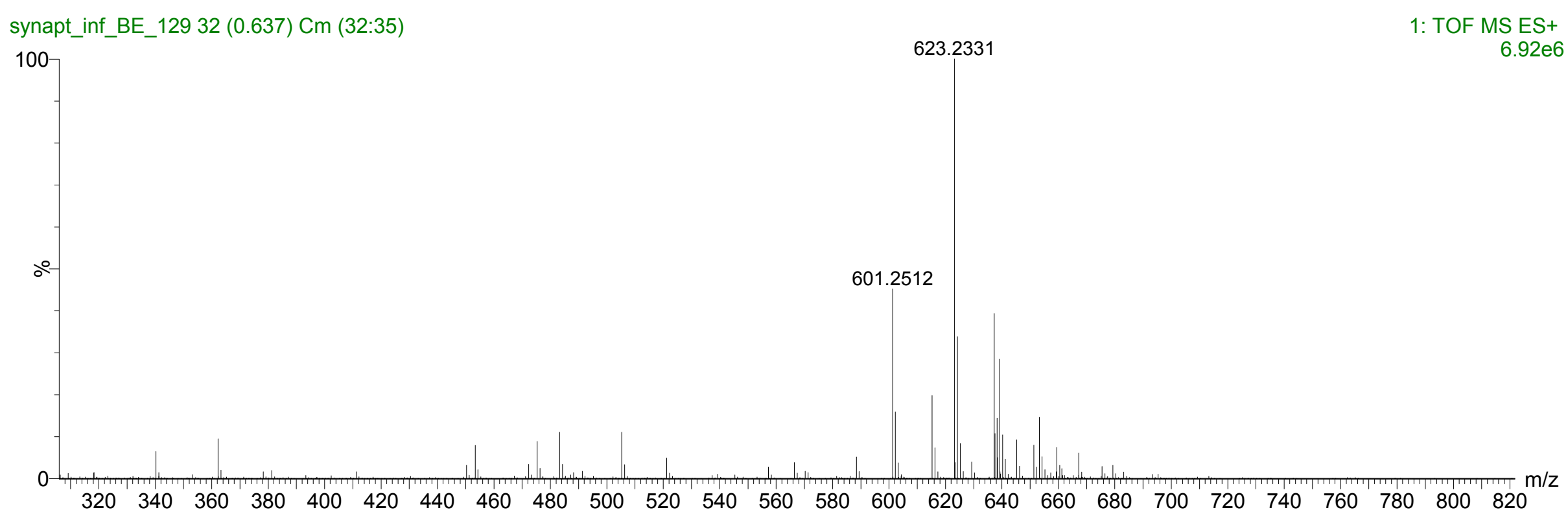

Mass Spectra of Gelator **3**.

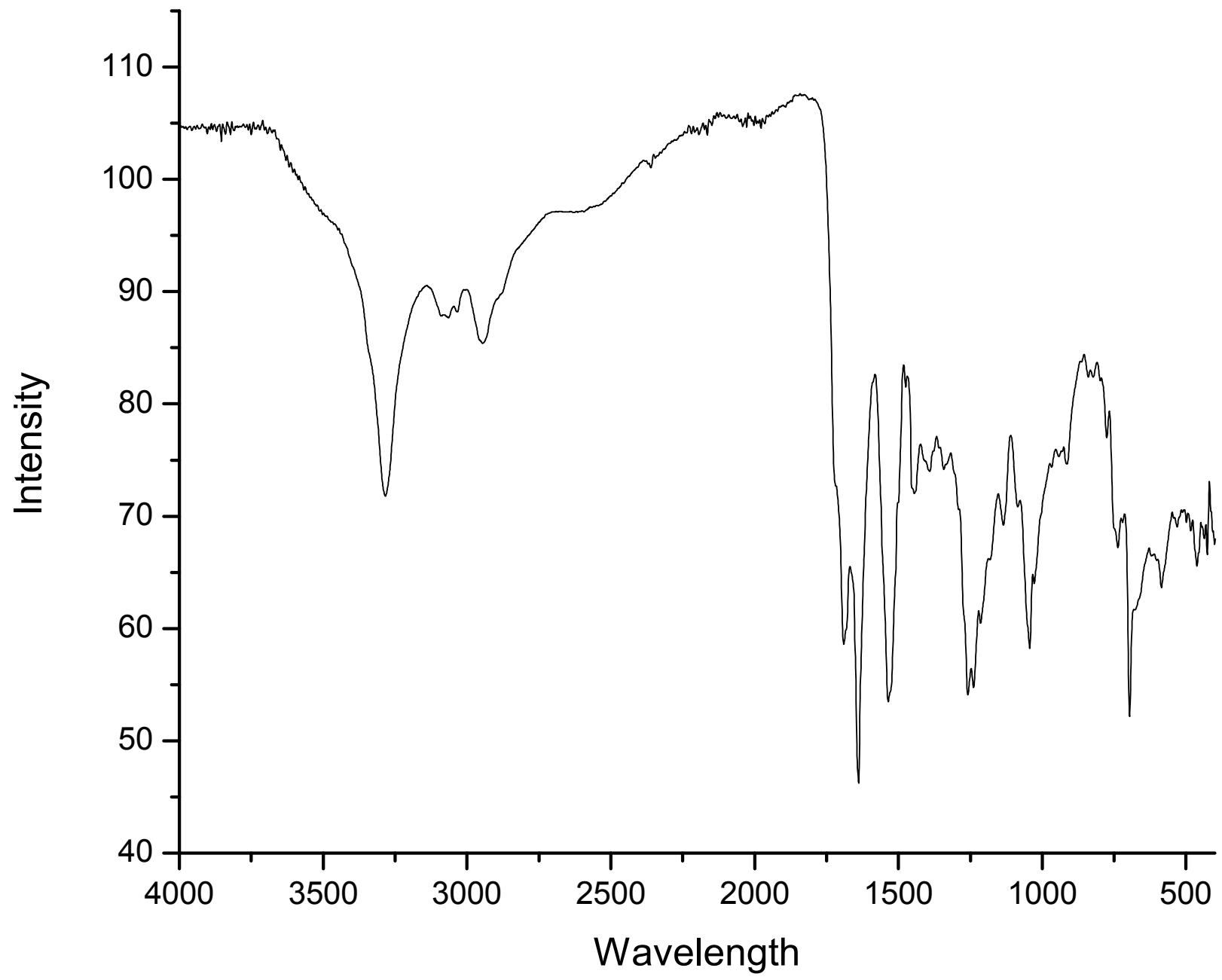

FTIR analysis of Gelator **3**.

Functional group	Peaks	Notes
**O–H stretch (COOH + OH)**	3528 (broad)	Strong, broad, overlaps N–H stretch
**N–H stretch (amide)**	3284	Merged with O–H stretch
**C=O stretch (amide I)**	1688	Strong, may overlap water ~1640
**N–H bend + C–N stretch (amide II)**	1532	Strong
**C–H stretch (aromatic)**	3034	Weak
**C–H stretch (aliphatic CH_2)**	2934	Weak
**Aromatic C=C stretch**	1636	Strong
**C–O stretch (COOH / alcohol)**	1258	Strong
**C-O-C Stretch (Benzyl Ether Linkage)**	1040	Strong

4) Hydrogel Characterization

Gel Formation: Hydrogel is formed using **Heat and Cool** method. The required amount of the gelator was taken in a standard 4.0 mL vial, and 1.0 mL of distilled water was added. The vial was closed, and the mixture was heated to complete dissolution, then allowing the solution to cool undisturbed to room temperature (~25 °C) without an actively controlled cooling rate. All samples were prepared under nominally identical ambient conditions to ensure consistency across comparisons. The solution was left undisturbed for gelation, and gel formation was confirmed by the inversion test.

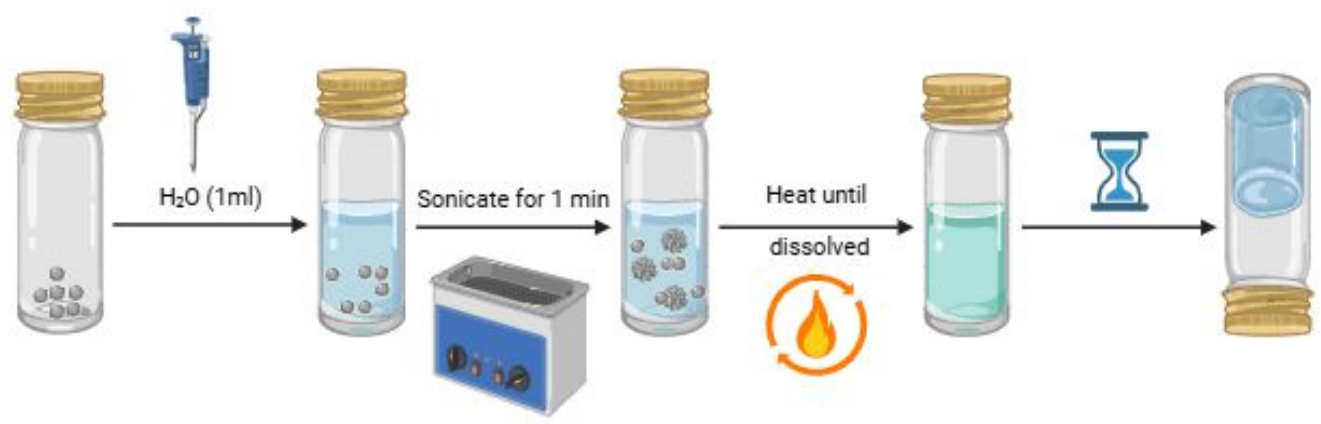

Schematic view of gel formation using heat and cool method.

Minimum Gel Concentration: Various amounts of gelator were weighed, and gelation experiment was carried out as described above. The minimum amount of gelator required to form gel after 1h was noted as minimum gel concentration (MGC) using vial inversion method.

Gelation temperature (Tgel) helps in evaluating the thermal stability and robustness of hydrogels. The thermal stability of the gels was studied by using the "vial inversion" method. The gelator at different concentration were taken in a standard 4.0 mL vial, and 1.0 mL of appropriate solvent was added. The mixture was heated to form a clear solution and kept undisturbed. After 24 h, vials were immersed in a water bath for 30 mins at a particular temperature and check gelation using vial inversion. Temperature of the water bath gradually increased by ~5 °C per 30 minutes. The temperature at which the vial doesn't pass the inversion test was recorded as Tgel.

pH and pKa Determination:

**3**	**8.3mM**	**3.6 pH**
**1**	**14mM**	**9.23 pH**
**2**	**2.5mM**	**6.56 pH**
**1+3**	**14+8.3 mM**	**4.72 pH**
**2+3**	**2.5+8.3mM**	**3.65 pH**
**1+2**	**14+2.5mM**	**9.3 pH**

pKa of compound 1 is 7.2 and for compound 3 is 4.15 calclated using potentiometric titration.

5) Solubility Studies

^{1}H NMR for solubility studies: All the gels were prepared in deuterated H_2O in vials at the required conditions and transferred to an NMR coaxial tube. The inlet tube was prepared with the required amount of Hydroquinone in Deuterated H_2O in order to compare the concentrations later. The gel is stabilized for at least 10 hours before doing the measurements in order to avoid the ageing effect. To understand this, we performed kinetics using ^{1}H-NMR over time to see the changes over time. For Gelator **1** both concentrations and temperature dependent NMR experiments have been performed to screen solubility.

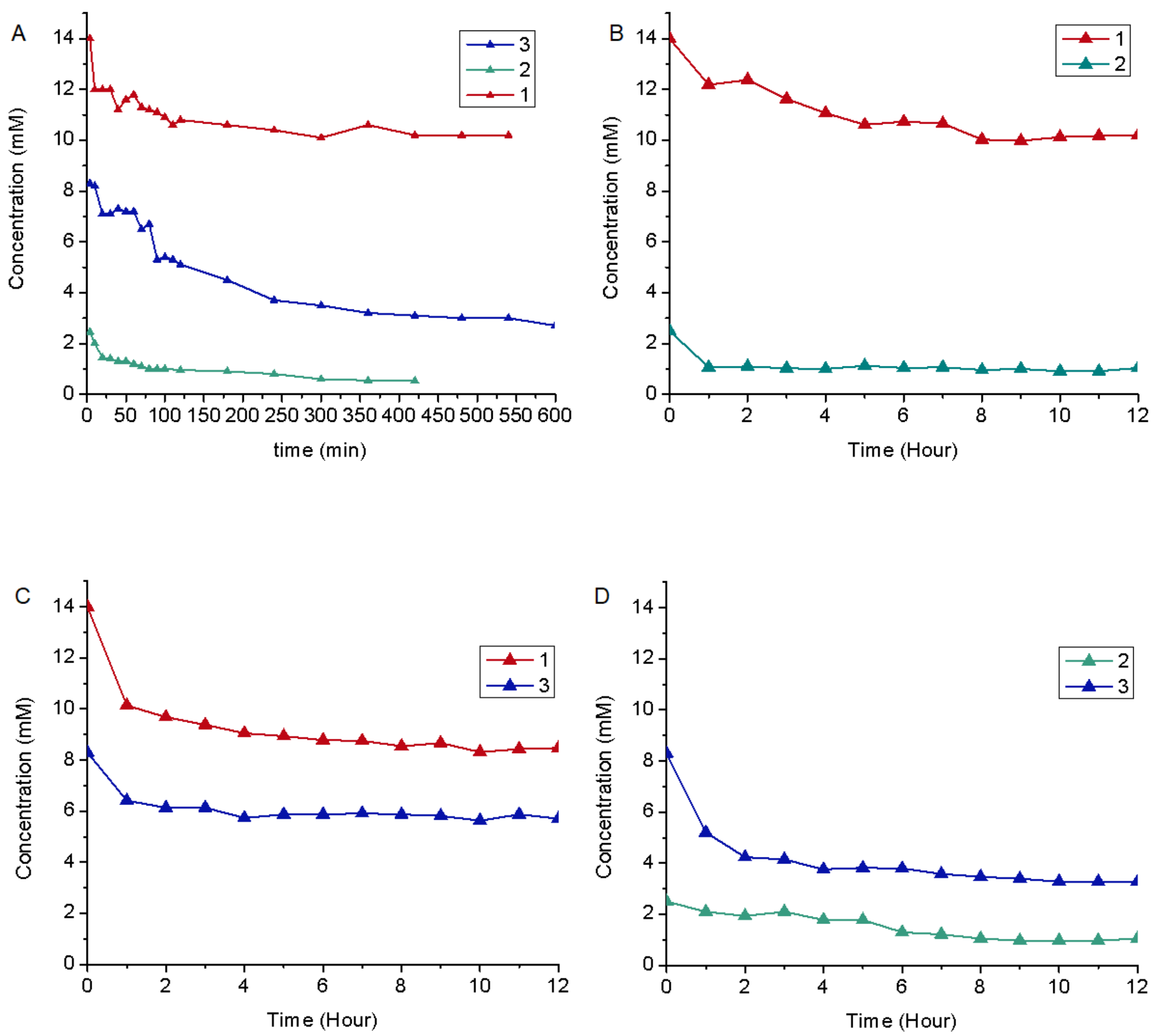

Kinetics study analysed using ^{1}H NMR, 400MHz at the minimum gelation concentration of each gelator alone (A) and in dual network (B-D) showing ageing of gel over time. A) For Gelator **1**, hydrogel was stabilized after ~150 mins, For gelator **2**, Hydrogel was stabilised after ~100 mins, and for Gelator **3**, hydrogel was stabilised after ~350mins. B) In *mix* **1+2**, 1 showed ageing over time until 8 hours whereas 2 was stable from the very start. C) In *mix* **2+3,** both of them were stable after 4 hours. D) Both gelators didn't show ageing after 6 hours. In order to avoid ageing, experiments were done after 16-20 hours, and only rheology experiments were done after 1 hour of gel formation.

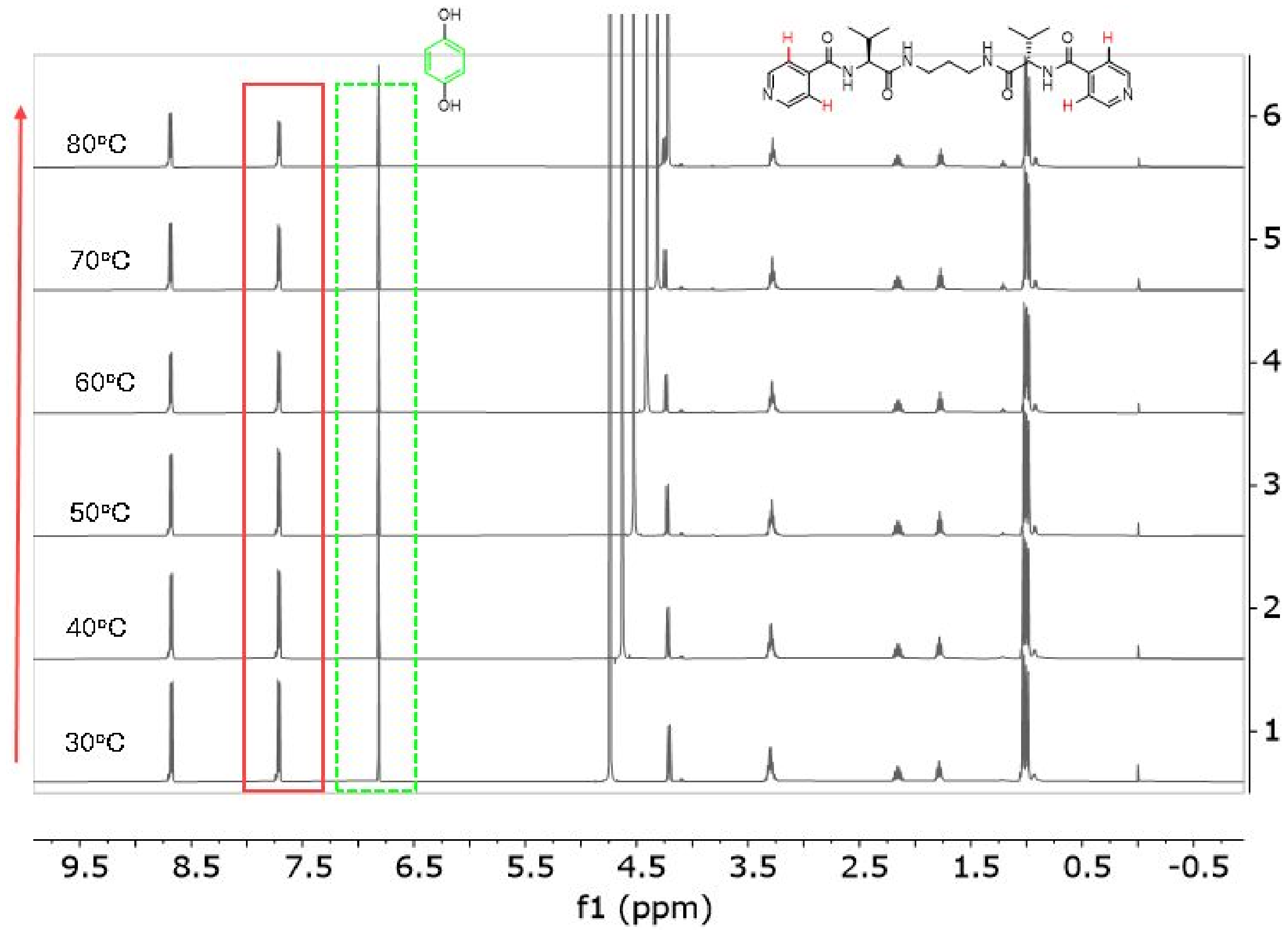

^{1}H NMR, 400MHz, D_2O spectra of Gelator **1** acquired at varying temperatures and concentration (14mM) to construct the solubility profile. Chemical shifts and peak broadening reflect temperature dependent changes, referencing is done using Hydroquinone.

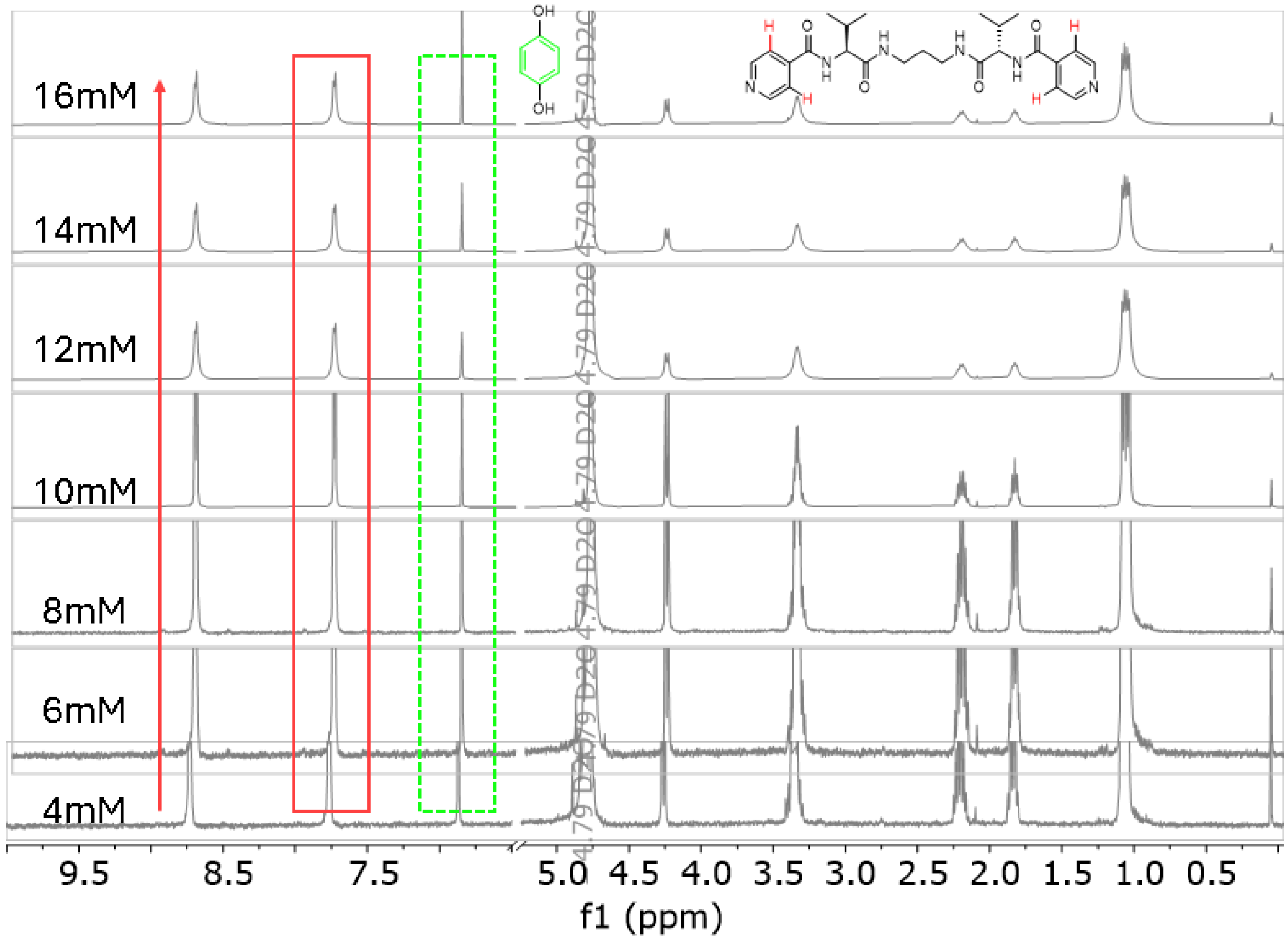

^{1}H NMR, 400MHz, D_2O spectra of Gelator **1** were acquired at varying concentrations and at a temperature of 30 °C to construct the solubility profile. Chemical shifts and peak broadening reflect concentration-dependent changes, referencing is done using Hydroquinone

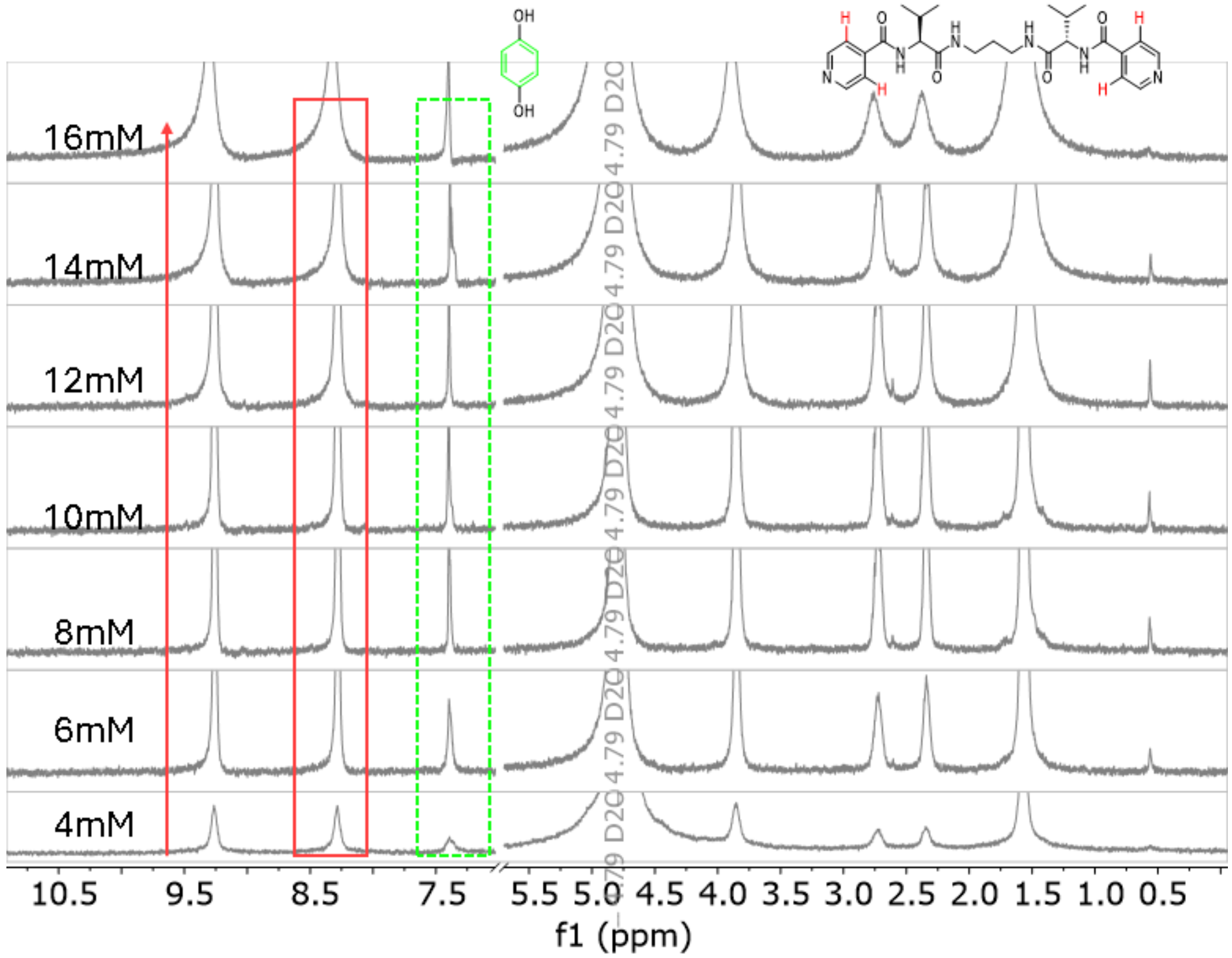

^{1}H NMR, 400MHz, D_2O spectra of Gelator **1** were acquired at varying concentrations and at a temperature of 80 °C to construct the solubility profile. Chemical shifts and peak broadening reflect concentration-dependent changes, referencing is done using Hydroquinone.

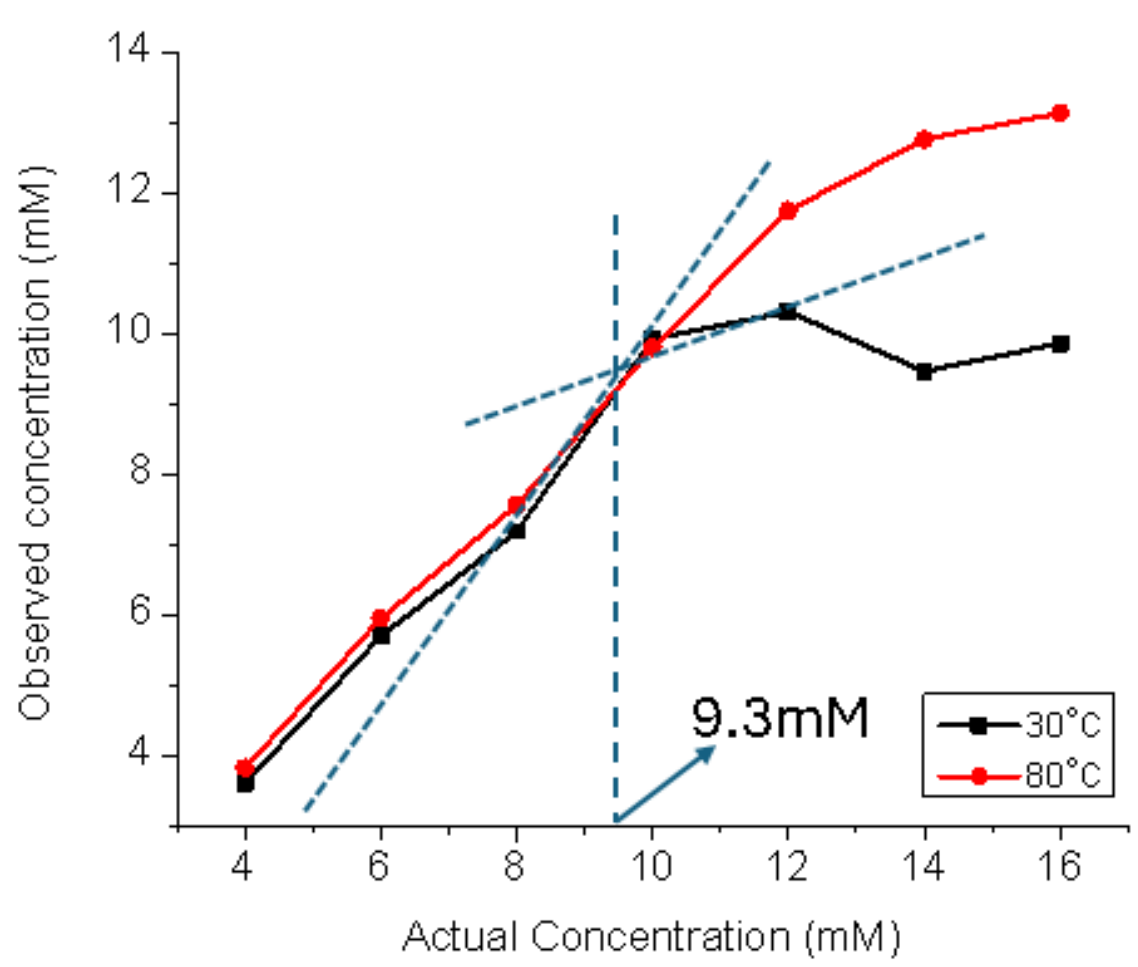

Solubility profile of Gelator **1** obtained at 30ºC and 80ºC at different concentrations obtained from integral peak at highlighted boxes in Figure S15 and Figure S16. The plateau between 9 to 13mM concentration indicative of metastability trapped assembly, extrapolating the slop gives solubility of 9.3mM at 30ºC.

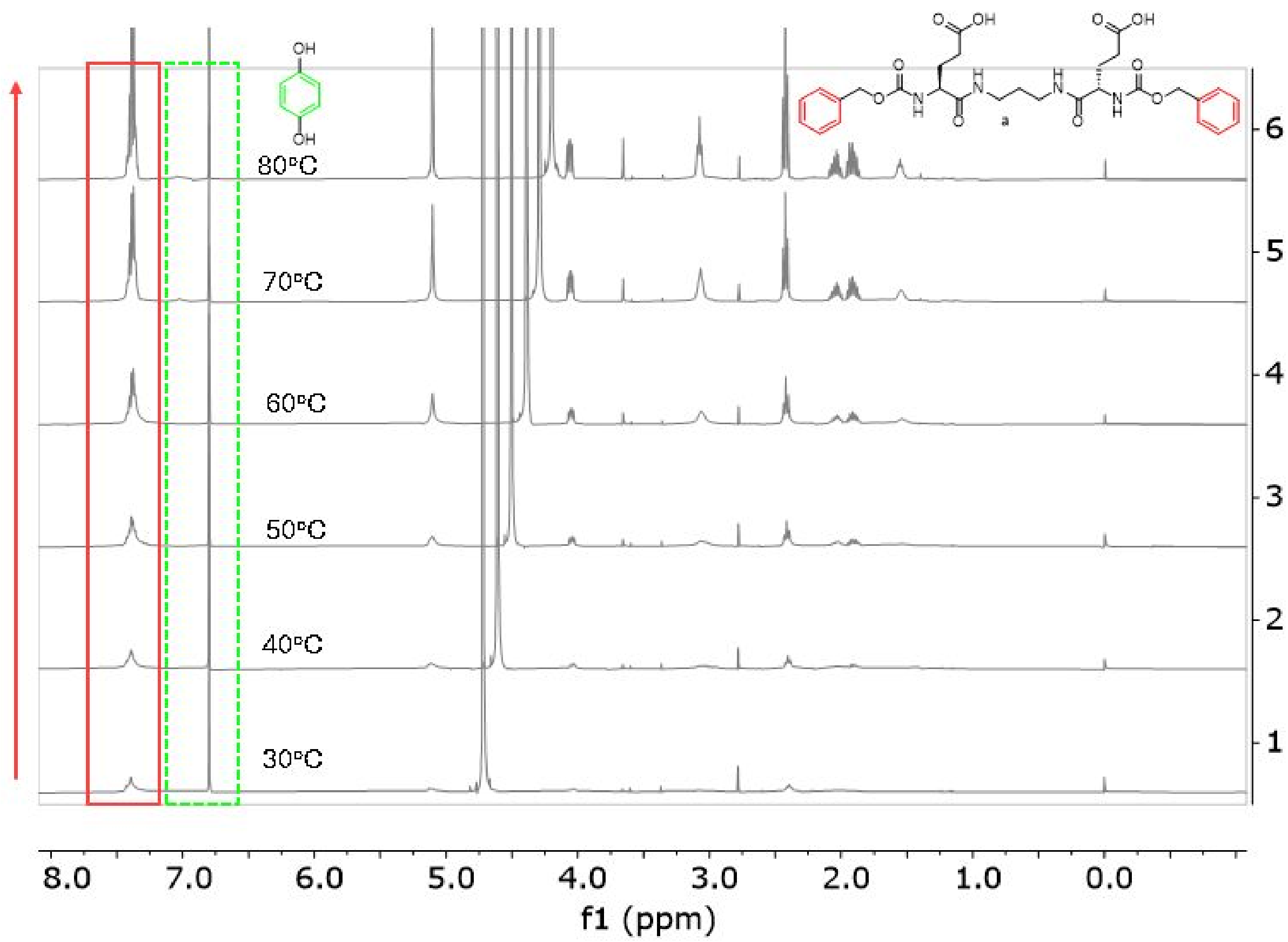

^{1}H NMR, 400MHz, D_2O spectra of Gelator **3** acquired at varying temperatures and concentration (8.3mM) to construct the solubility profile. Chemical shifts and peak broadening reflect temperature dependent changes; referencing is done using Hydroquinone.

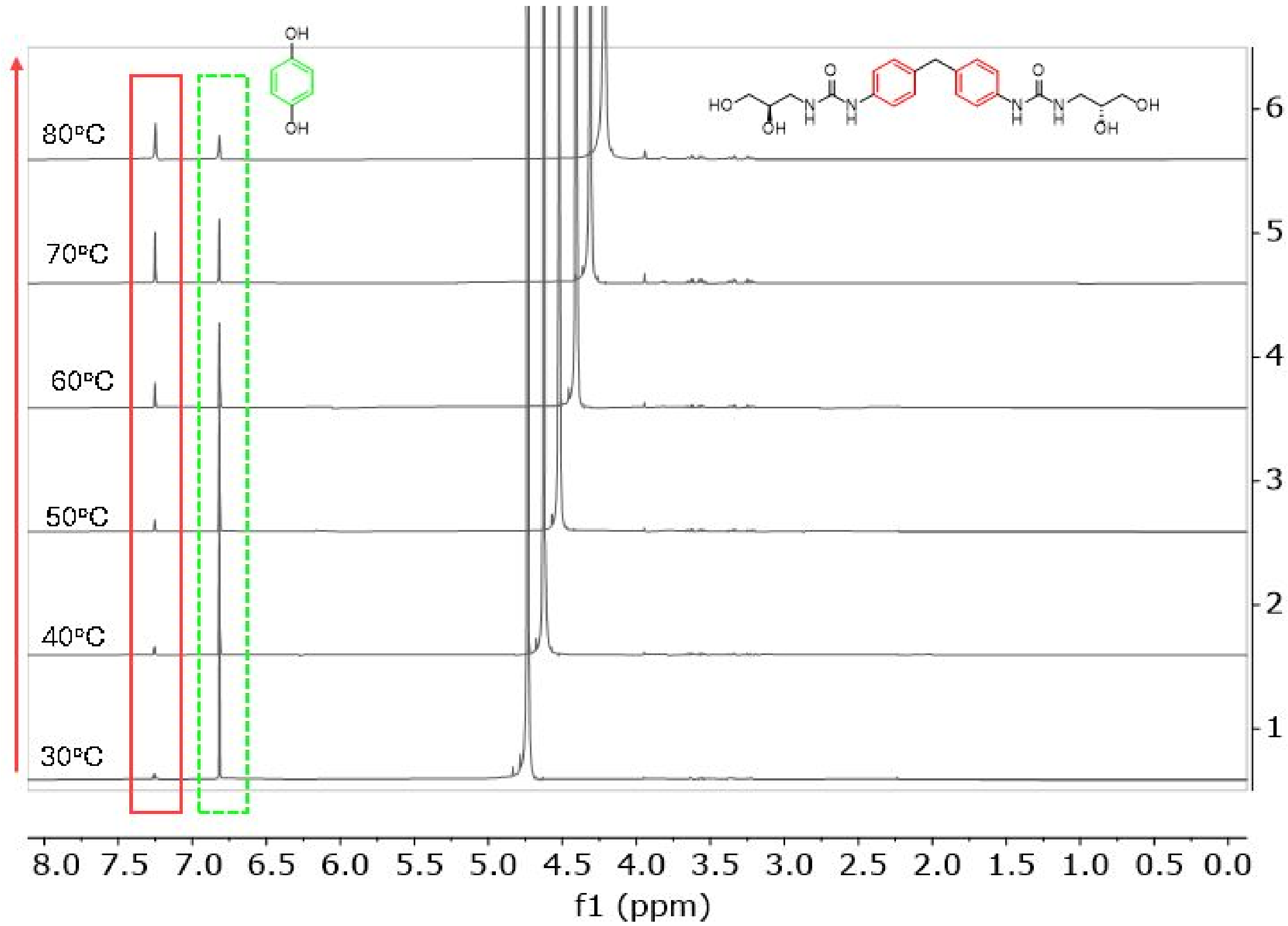

^{1}H NMR, 400MHz, D_2O spectra of Gelator **2** acquired at varying temperatures and concentration (2.5mM) to construct the solubility profile. Chemical shifts and peak broadening reflect temperature- dependent changes, referencing is done using Hydroquinone.

Vant Hoff equation: The reported thermodynamic data were obtained considering the following equations (K_s = solubility of the gel network.

G = Gibbs free energy; R = gas constant; T = temperature; H = enthalpy and S = entropy.

$$\Delta G = \Delta H - T\Delta S$$

$$Ks = e^{-\Delta G/RT}$$

$$\ln(Ks) = \left(-\frac{\Delta H}{RT}\right)\frac{1}{T} + \frac{\Delta S}{R}$$

$$\frac{\partial \ln(Ks)}{\partial T} = \left(\frac{\Delta H}{R}\right)\frac{1}{T^2} \qquad \text{(Equation S1)}$$

So by plotting logarithm of solubility against 1/T, we can get enthalpy and entropy factor.

6) Dual component Hydrogel formation

We tried dual component gelation at different concentrations and after several trials came to the conclusion that for *mix* **1+2** and *mix* **2+3** we need both the gelators at their minimum gelation concentration, whereas for mix 1+3 we can get gel at 8.3mM each which is minimum gelation concentration for gelator **3.**

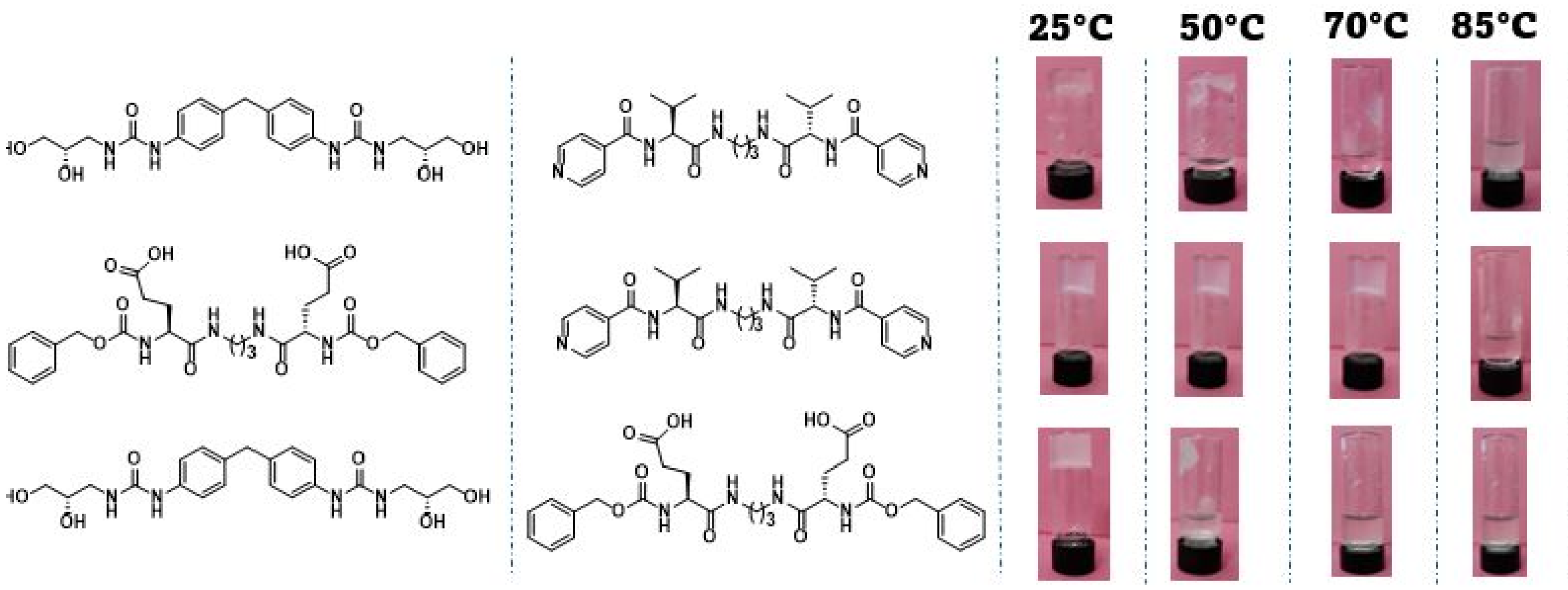

Visual assessment of the dual network's thermal response at different temperatures, conducted at each component's minimum gelation concentration (MGC) to monitor gel-sol transitions and structural changes

7) Solubility Studies for dual component

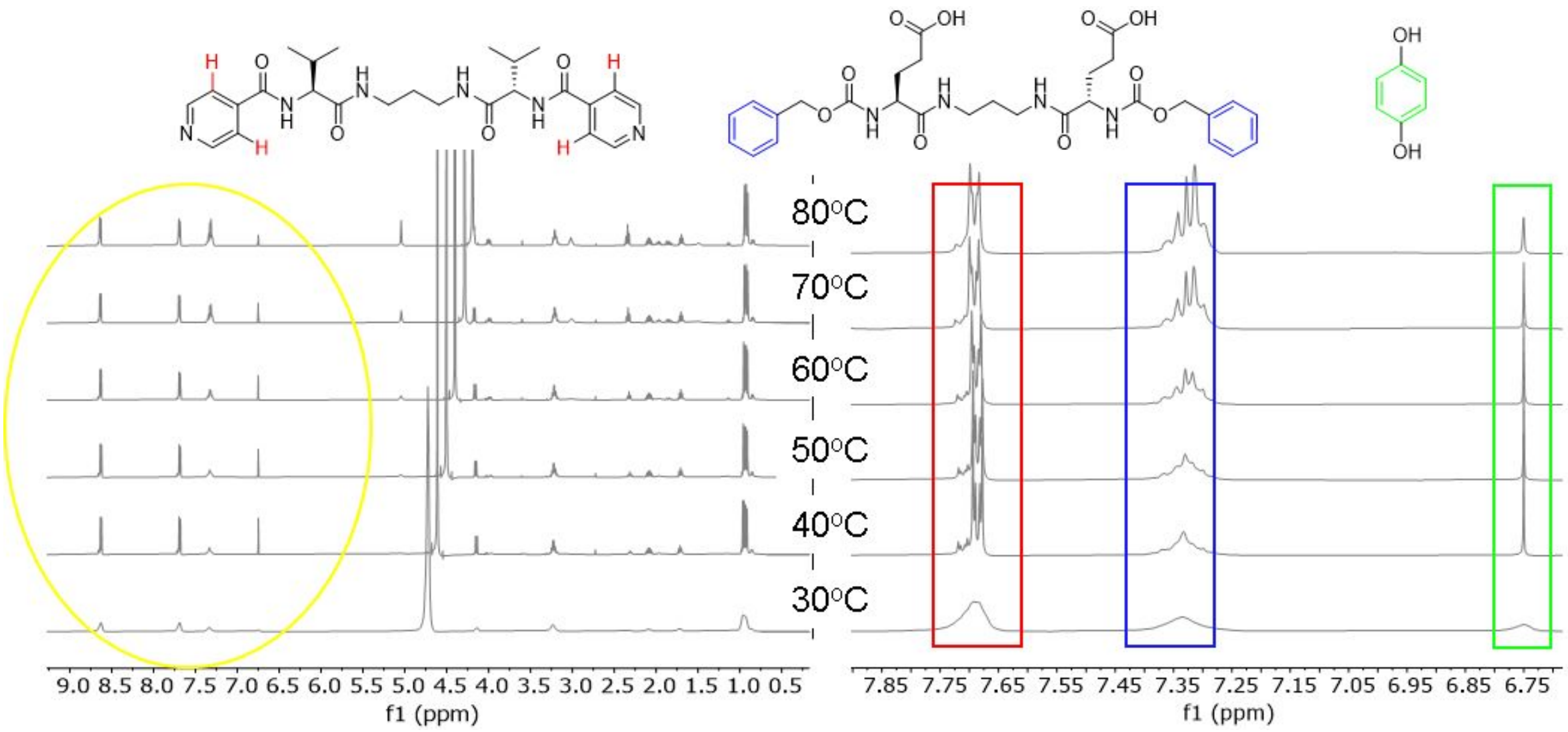

^{1}H NMR, 400MHz, D_2O spectra of the dual-component system *mix* **1 + 3** at their respective minimum gelation concentrations (MGC) for Gelator **1**- 14mM and For Gelator **3**- 8.3mM, with hydroquinone as an internal standard, acquired at variable temperatures.(Left) Full spectral range showing the temperature-dependent evolution of peaks, with the yellow circle highlighting the broad signals at low temperature attributed to the assembled state. (Right) Expanded aromatic region showing the emergence and sharpening of peaks corresponding to the free monomeric species upon heating.

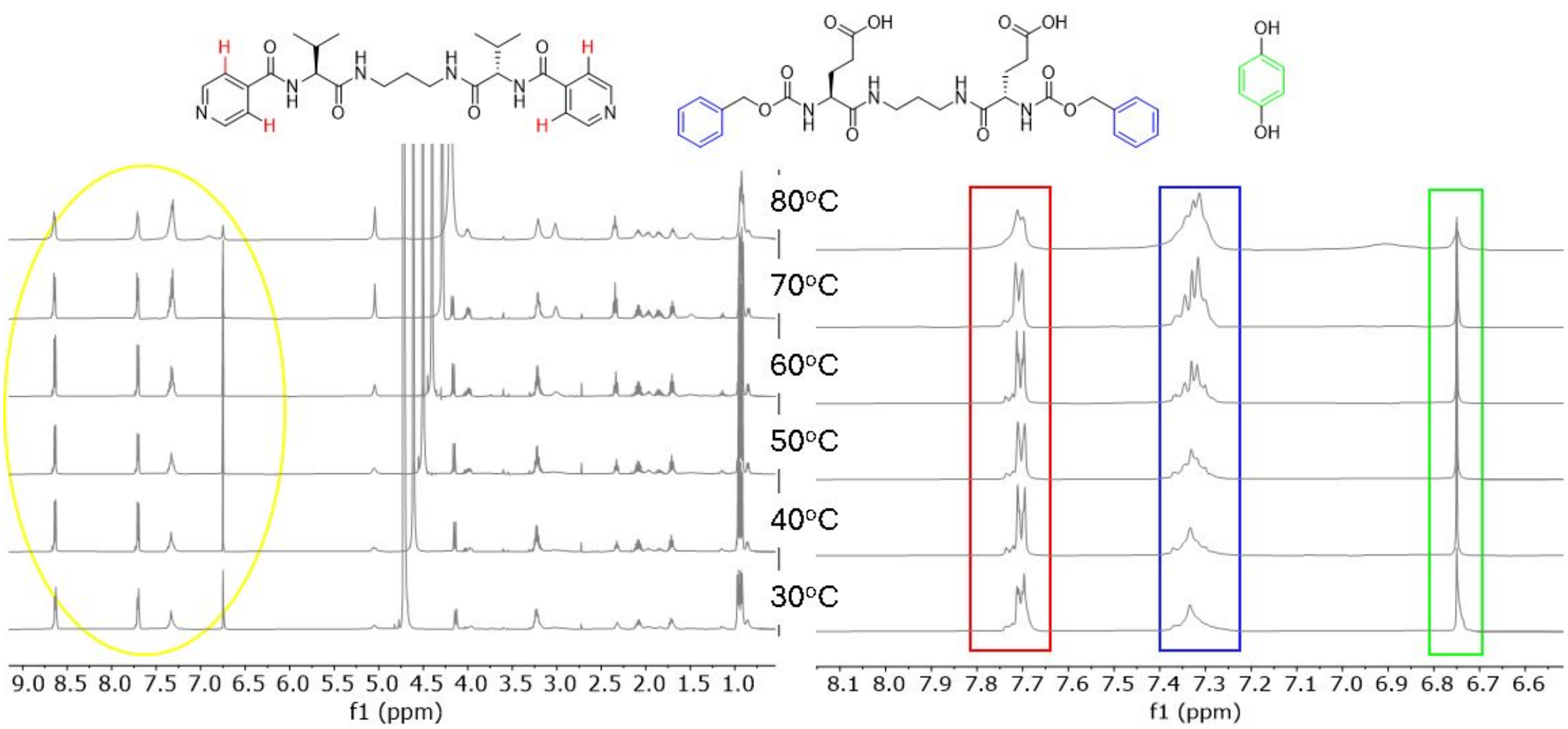

^{1}H NMR spectra of the dual-component system *mix* **1 + 3** at the minimum gelation concentrations (MGC) of **3** (8.3mM each), with hydroquinone as an internal standard, acquired at variable temperatures.(Left) Full spectral range showing the temperature-dependent evolution of peaks, with the yellow circle highlighting the broad signals at low temperature attributed to the assembled state. (Right) Expanded aromatic region showing the emergence and sharpening of peaks corresponding to the free monomeric species upon heating.

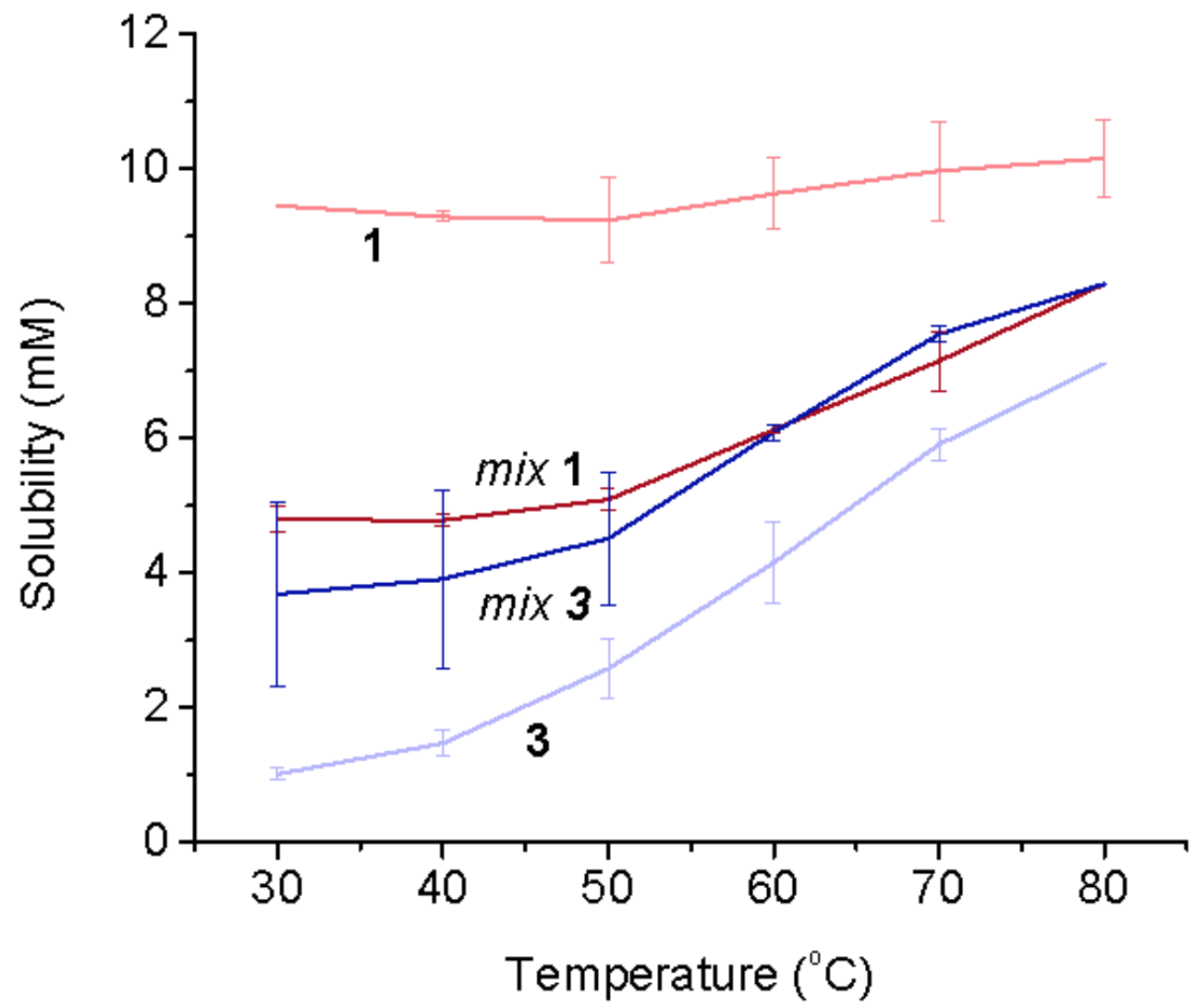

Figure S 1: Solubility profile of *mix* **1+3** at MGC of **3** (8.3mM each), compared to the solubility profiles of gelator **1** and gelator **3** individually. In the mixture, both gelators exhibit coupled solubility behavior, entering and leaving the fiber network cooperatively, indicative of co-assembly rather than orthogonal self-assembly.

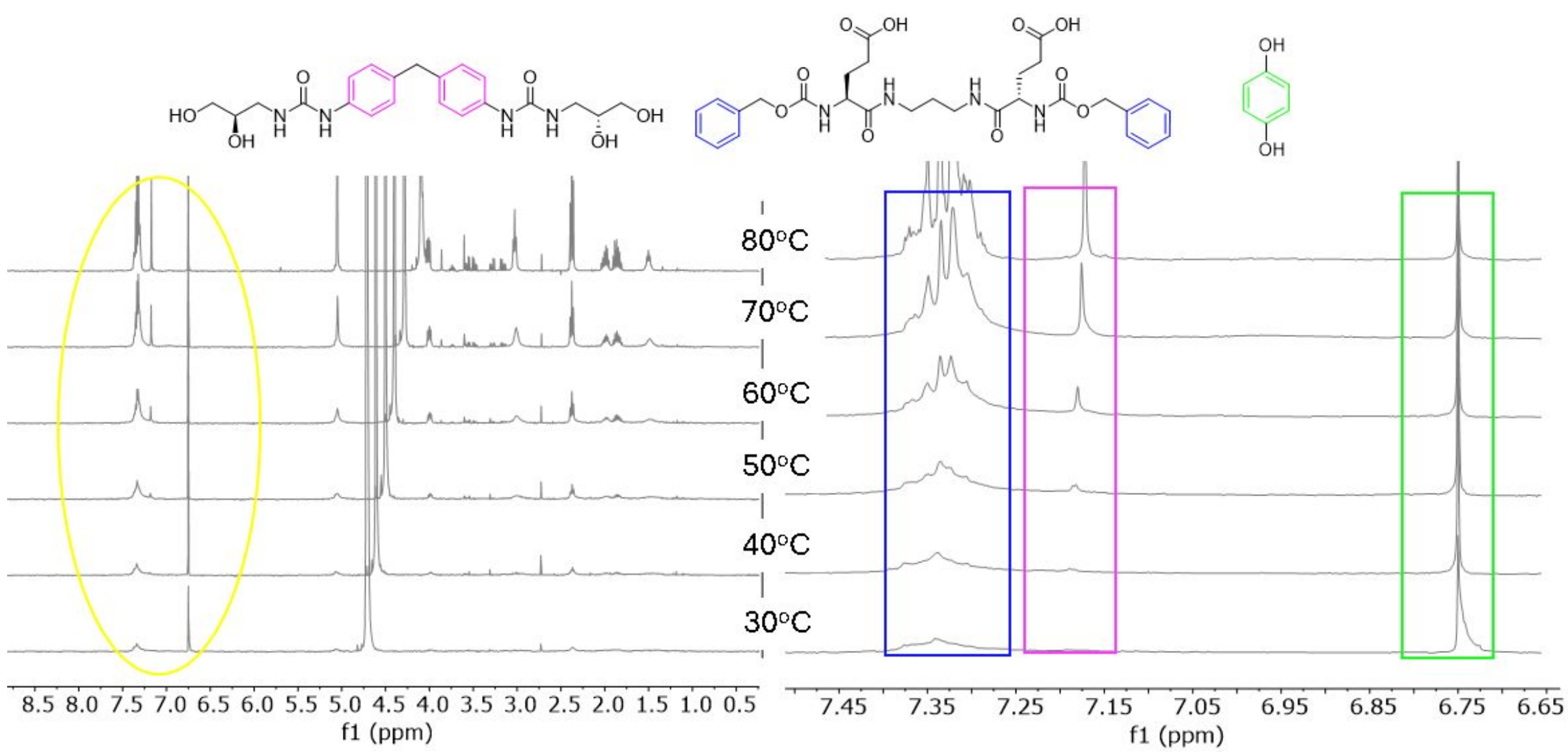

^{1}H NMR spectra of the dual-component system *mix* **2 + 3** at their respective minimum gelation concentrations (MGC) with hydroquinone as an internal standard, acquired at variable temperatures.

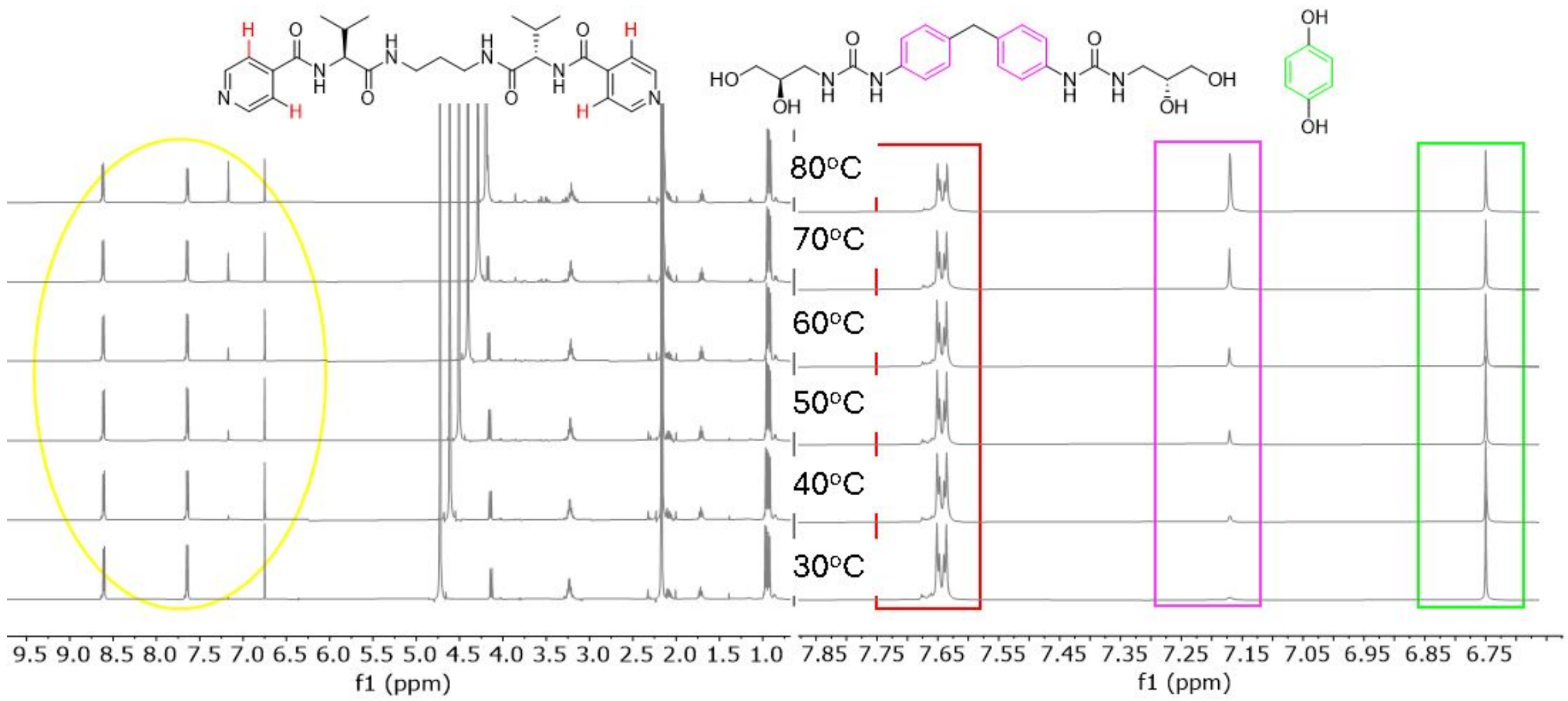

^{1}H NMR spectra of the dual-component system *mix* **1 + 2** at their respective minimum gelation concentrations (MGC), with hydroquinone as an internal standard, acquired at variable temperatures.

8) NMR Titration of Gelator 1 and 3

To gain deeper insight into the co-assembly behavior of gelators **1** and **3**, NMR titration experiments were performed. The observed downfield shift of the CH_2 protons adjacent to the carboxylate group in gelator **3**, along with the corresponding shift in the pyridine protons of gelator **1**, clearly indicates the formation of a salt bridge between the carboxylate moiety of gelator **3** and the pyridine unit of gelator **1**. Furthermore, a Job's plot constructed from the NMR data revealed a 1:1 binding stoichiometry between the two gelators.

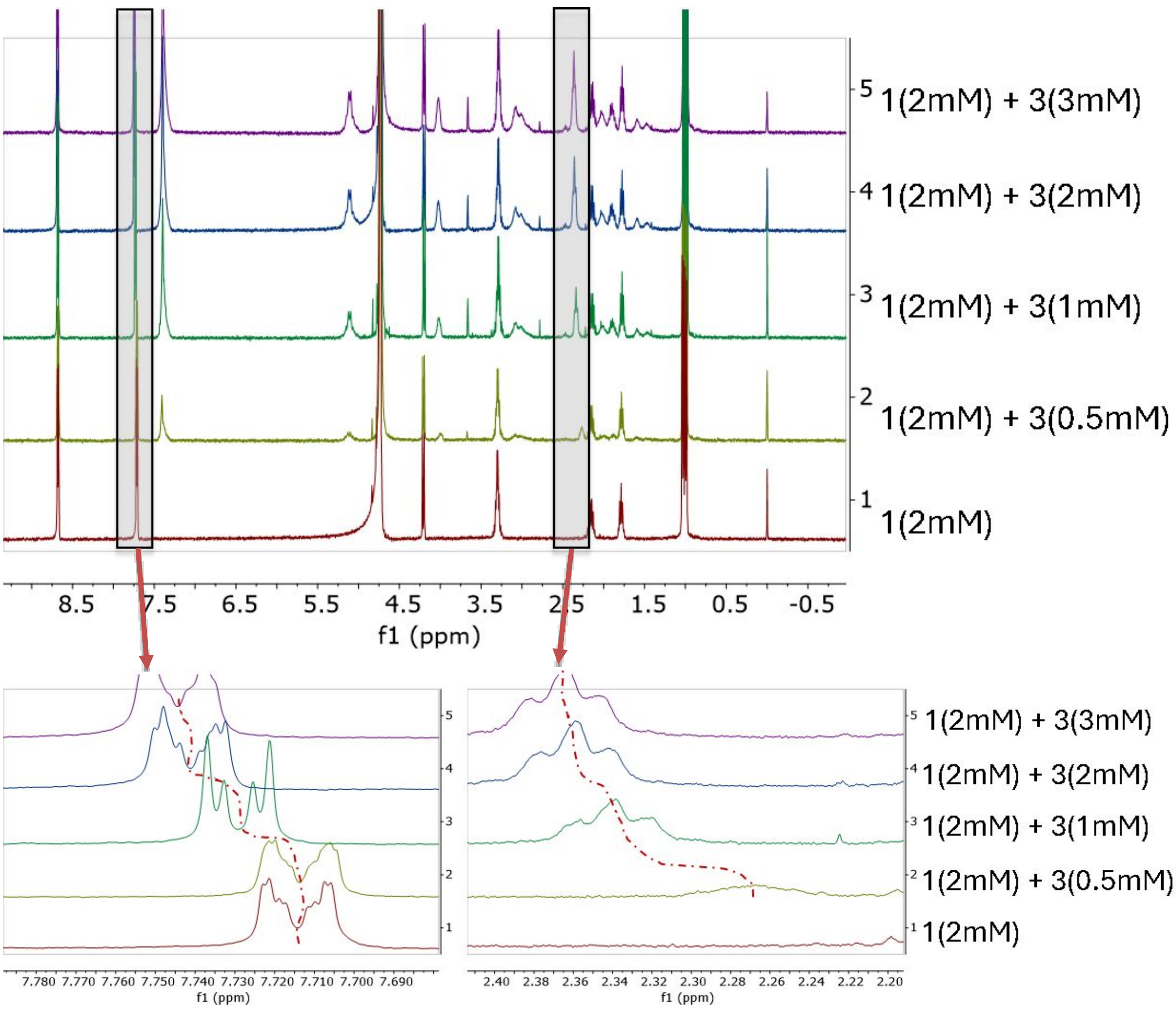

^{1}H NMR titration performed between two gelators **1** and **3**, starting from 2mM of gelator **1** to increasing concentration of gelator **3** on top and on bottom, is shown the enhanced region of signals for the pyridine group from gelator **1** at 7.7ppm and the alkyl group next to the carboxylate group in gelator **1** at 2.36ppm.

Jobs Plot for co-assembly

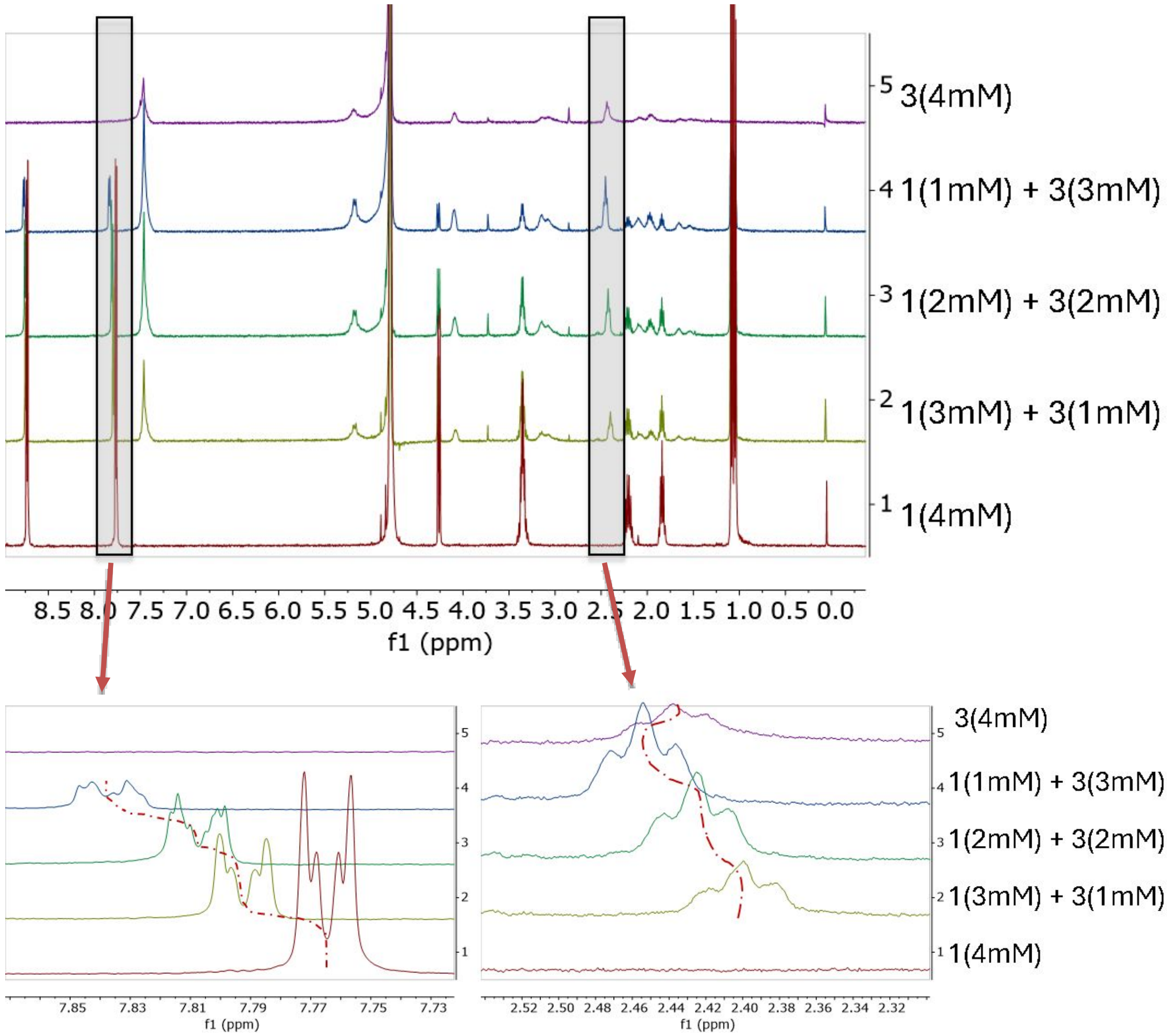

^{1}H NMR titration performed between two gelators **1** and **3**, starting from 4mM of gelator **1** to increasing concentration of gelator **3** on top maintain the total concentration to be 4mM and on bottom is shown the enhanced region of signals for pyridine group from gelator **1** at 7.7ppm and alkyl group next to carboxylate group in gelator **1** at 2.4ppm.

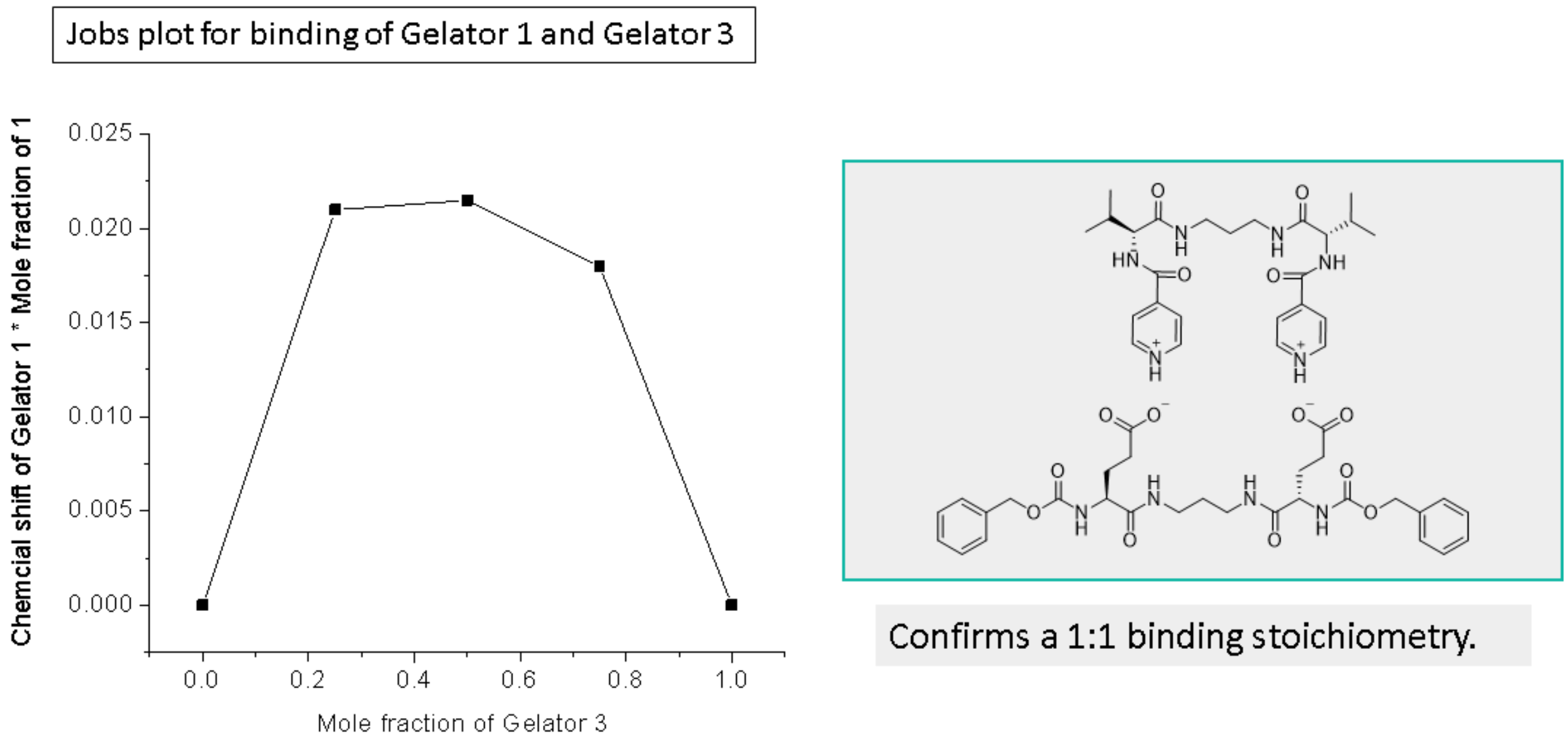

Figure S 2: Jobs plot analysed from ^{1}HNMR Titration of **1** and **3** maintaining 4mM concentration as total at all points, x-axis shows mole fraction of gelator **3** and y-axis shows change in chemical shift of gelator **1** with change in mole fraction of gelator **1**. The graph clearly demonstrate the binding between two gelators to be 1:1.

9) DOSY Spectra

DOSY NMR experiments were conducted as a function of a range of gelator concentrations that span the sol–gel transition in order to probe the molecular-level organization within the multicomponent system. The diffusion coefficient, D, is a function of the hydrodynamic size of a species and is inversely proportional to both the effective radius of that species and the viscosity of the medium, as described by the Stokes–Einstein relationship.

By analyzing the concentration dependence of D for each gelator, we assessed whether the two components diffuse as one supramolecular entity-evidencing co-assembly-or whether they maintain distinct diffusion behaviors, consistent with self-sorting and independent incorporation into separate fibrillar domains. Matching or convergent D values strongly support co-assembled structures with shared dynamic exchange, whereas divergent D values imply that the components participate in separate networks or remain partially monomeric. This approach therefore provides direct insight into whether the gelators enter and exit the fibers cooperatively or exchange with the solution phase as independent monomeric species.

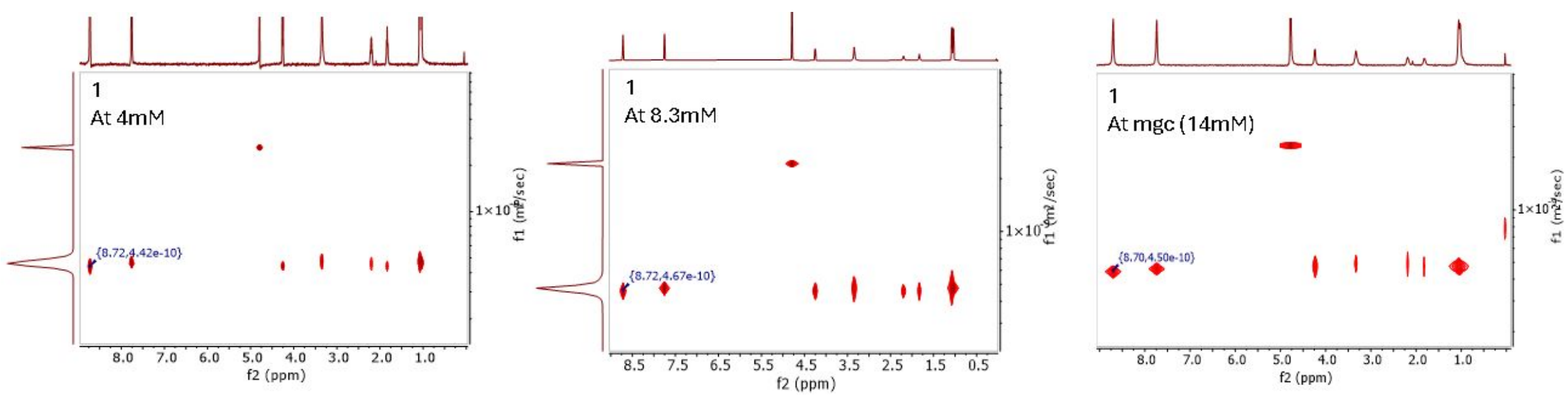

Figure S 3: DOSY spectra of compound **1** at different concentrations, D value at 4mM is 4.42*10^{-10} m^2/sec, at 8.3Mm is 4.67 *10^{-10} m^2/sec and at 14mM is 4.5 *10^{-10} m^2/sec.

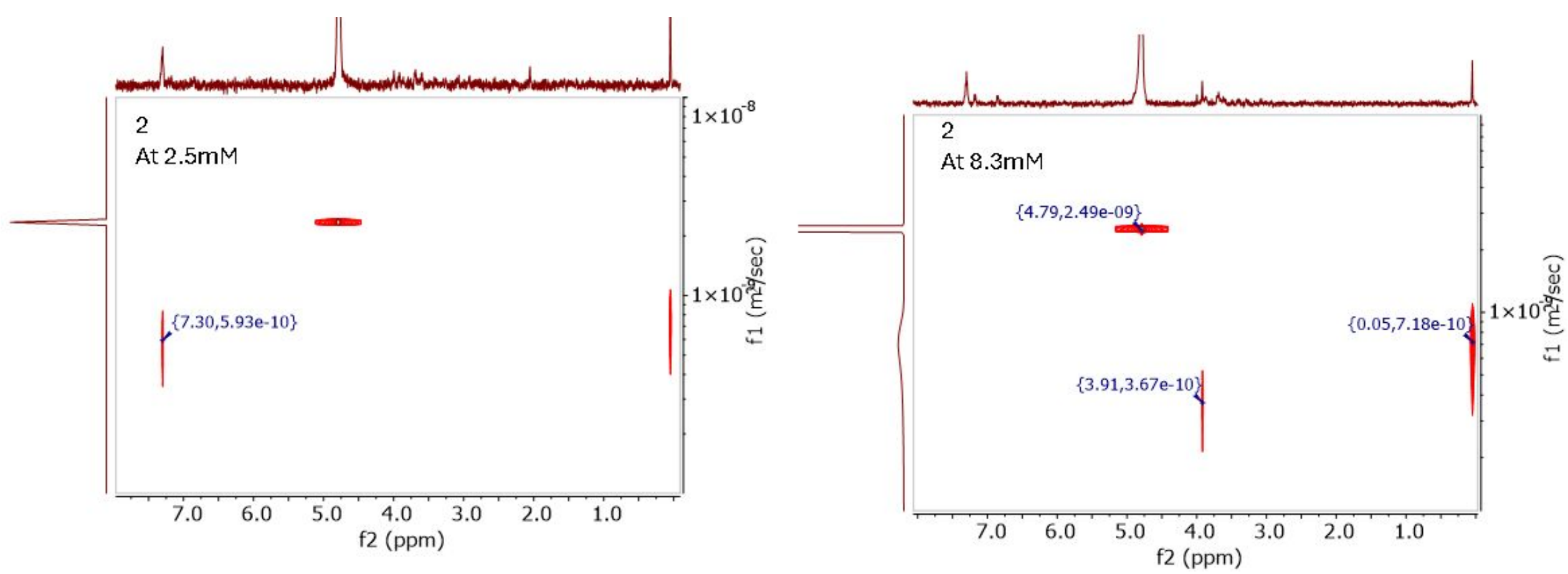

Figure S 4: DOSY spectra of compound **2** at different concentrations, D value at 2.5mM is 5.93**10^{-10} m^2/sec., at 8.3Mm is 3.67 **10^{-10} m^2/sec.and at 14mM is 4.5 **10^{-10} m^2/sec..

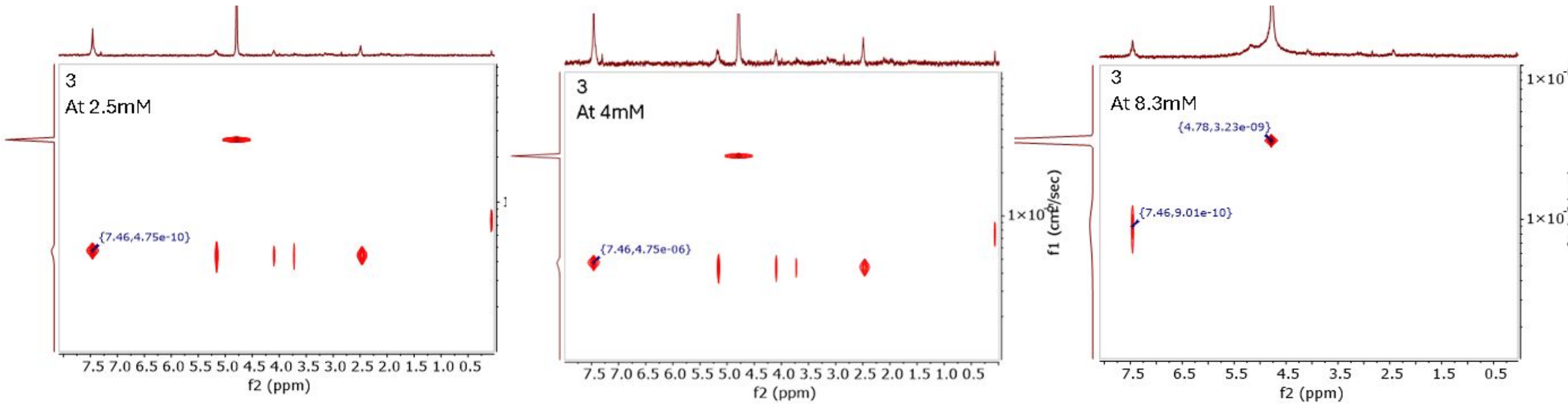

Figure S 5: DOSY spectra of compound **3** at different concentrations showing D value at 2.5mM is 4.75*10^{-10} m^2/sec., at 4mM is 4.75 *10^{-10} m^2/sec. and at 8.3mM is 9.01 *10^{-10} m^2/sec.

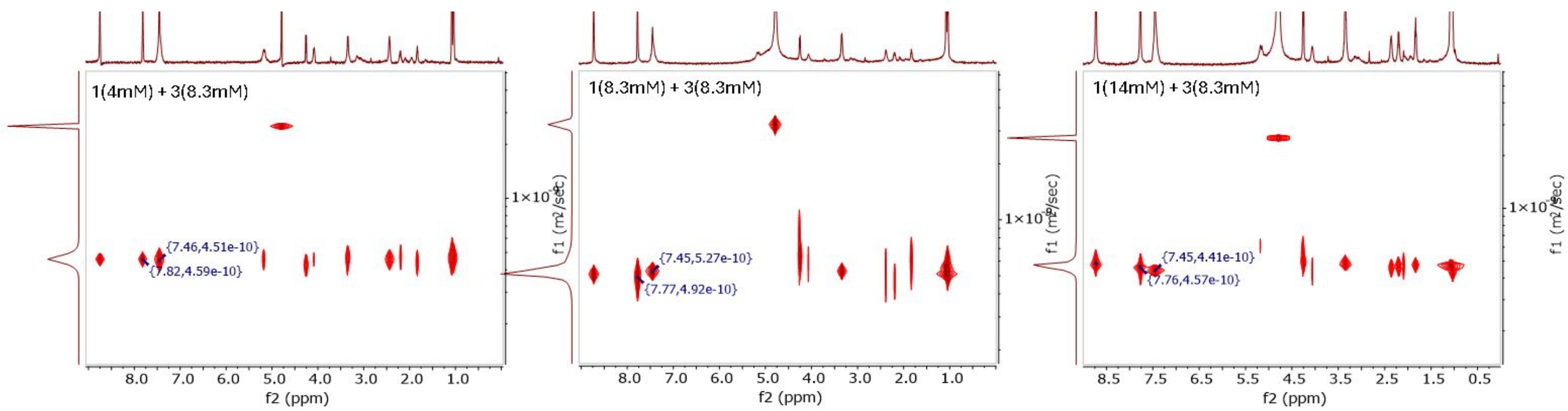

Figure S 6: DOSY spectra of *mix* **1+3** at different concentrations showing D value at 1(4mM) + 3(8.3mM) is 4.59*10^{-10} m^2/sec for compound **1** and 4.51*10^{-10} m^2/sec for compound **3**; 1(8.3mM) + 3(8.3mM) is 4.92*10^{-10} m^2/sec for compound **1** and 5.2*10^{-10} m^2/sec for compound **3**; **1**(14mM) + **3**(8.3mM) is 4.57*10^{-10} m^2/sec for compound **1** and 4.41*10^{-10} m^2/sec for compound **3**. In all the cases they share similar D value suggesting a co-assembly.

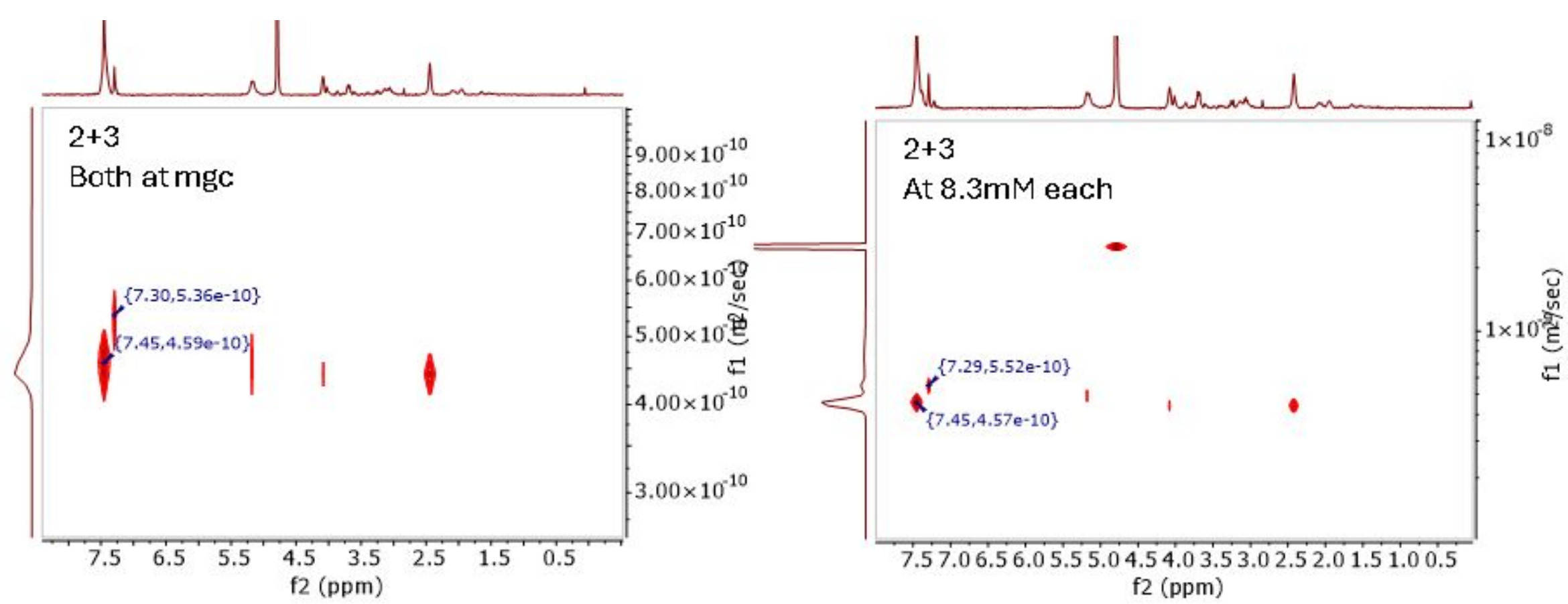

Figure S 7: DOSY spectra of *mix* **2+3** at different concentrations showing D value at **2**(2.5mM) + **3**(2.5mM) is 4.59*10^{-10} m^2/sec for compound **2** and 5.36*10^{-10} m^2/sec for compound **3**; **2**(8.3mM) + **3**(8.3mM) is 4.57*10^{-10} m^2/sec for compound **2** and 5.52*10^{-10} m^2/sec for compound **3**. Different D values suggesting self-sorted system.

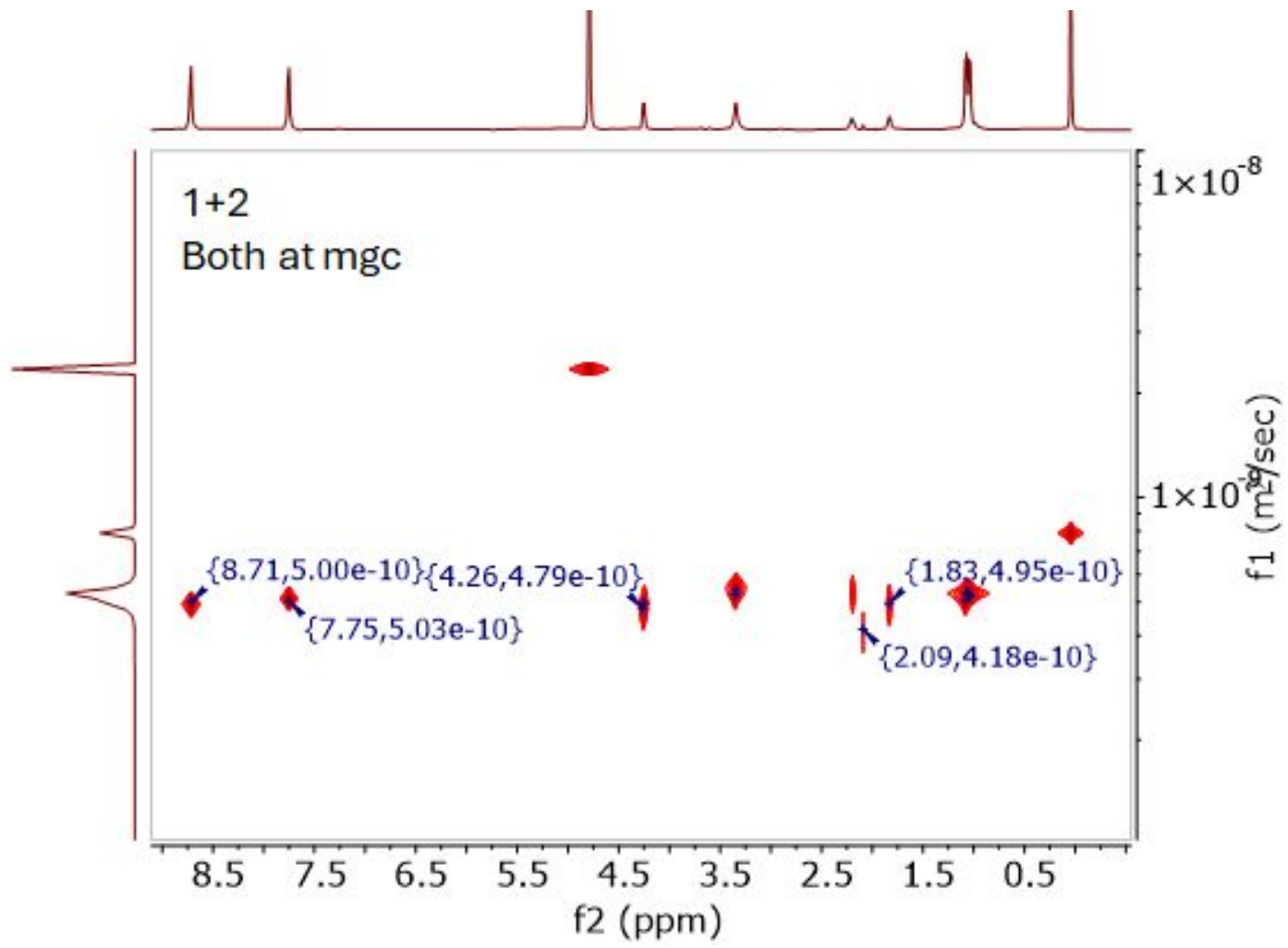

Figure S 8: DOSY spectra of *mix* **1+2** at different concentrations showing D value at **1**(14mM) + **2**(2.5mM) is $5*10^{-10}$ m^2/sec for compound **1** and $4.18*10^{-10}$ m^2/sec for compound **2.** Different D values suggesting self-sorted system.

10) Microscopy Analysis

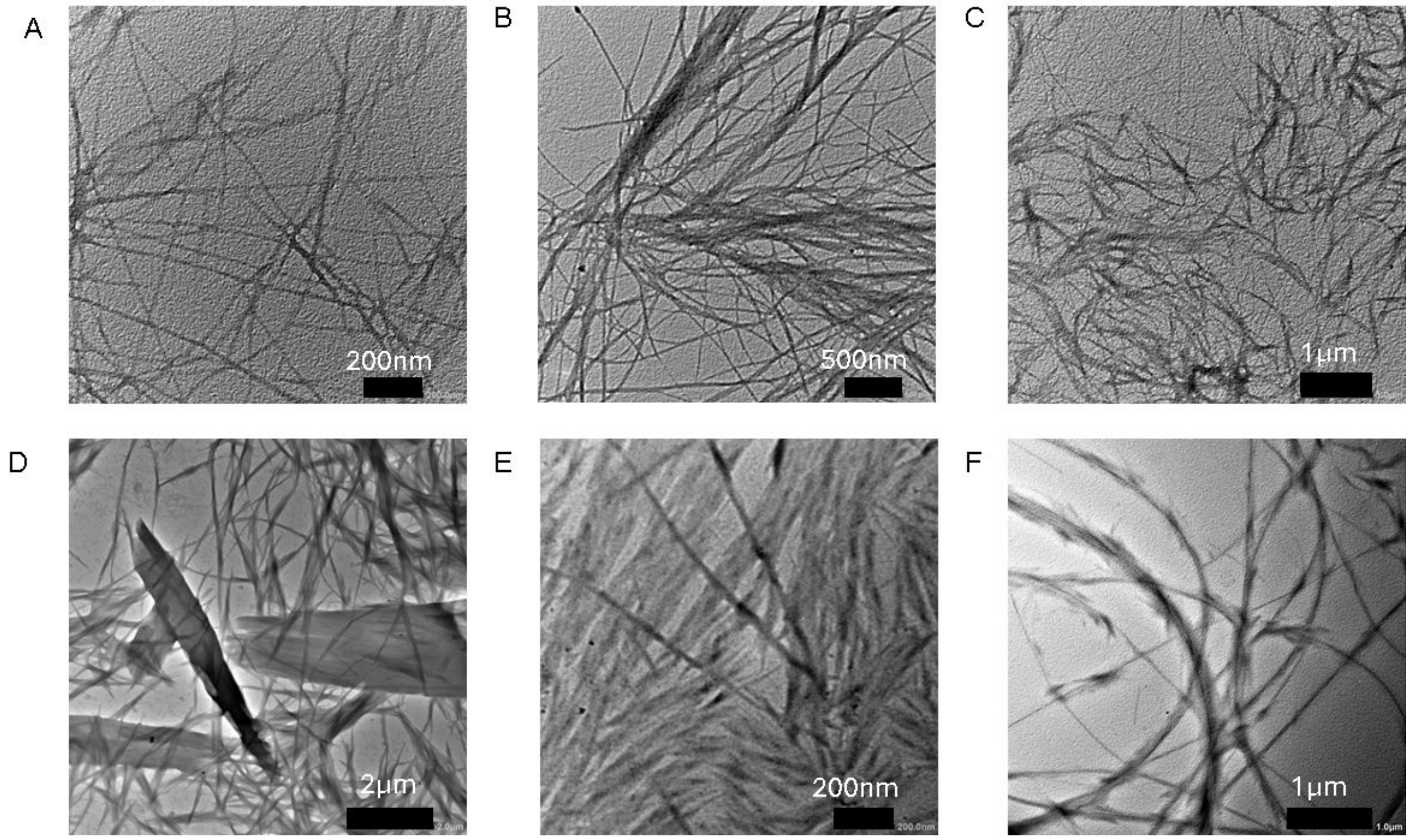

TEM image of air dried xerogel, A) Gelator **1**. B) Gelator **2**. C) Gelator **3**. D) *mix* **1+2**. E) *mix* **1+3** F) *mix* **2+3** at their respective minimum gelation concentration alone or when in combination.

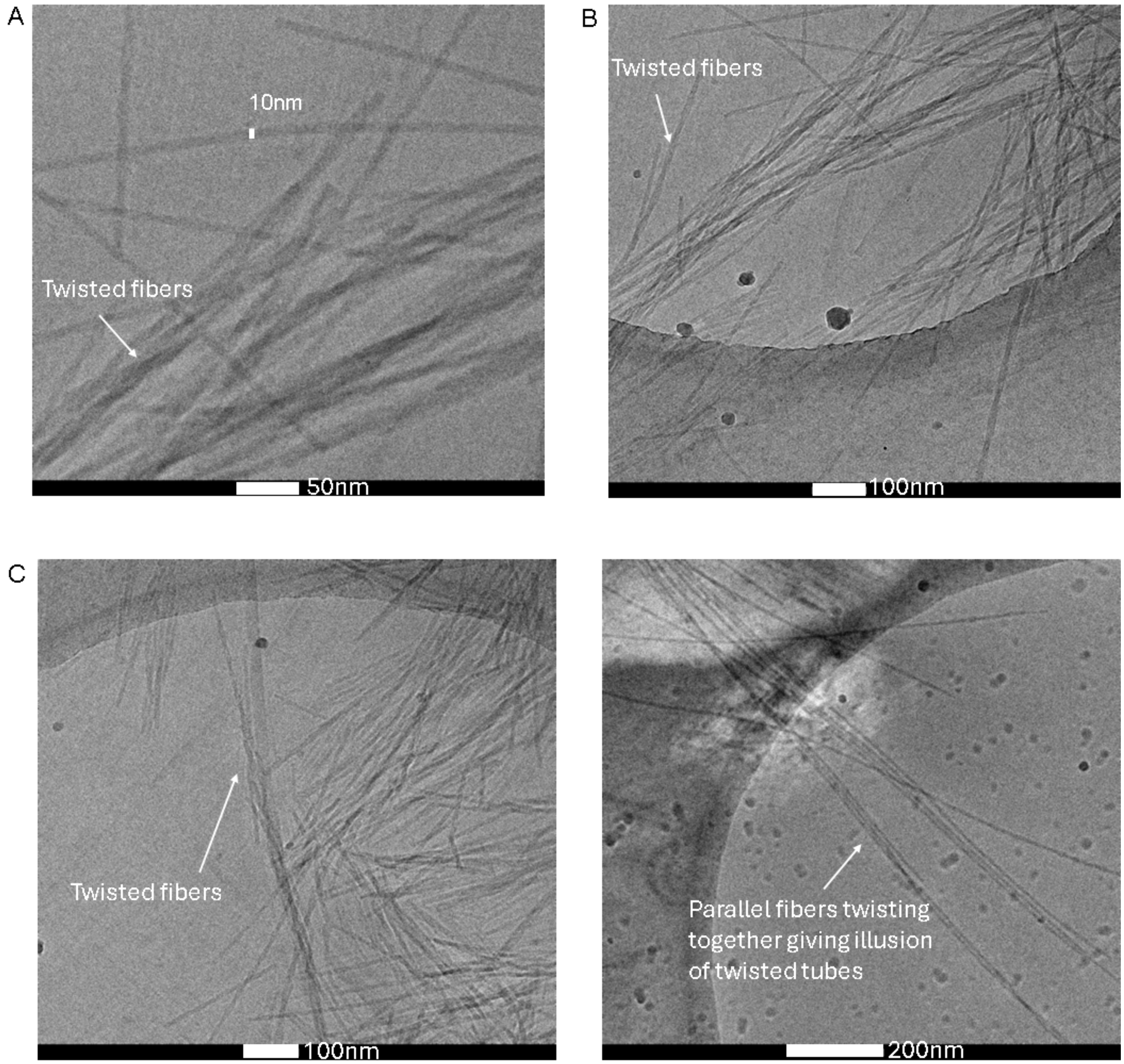

Cryo-TEM of Gelator **1** at minimum gelation concnetration (14mM) showing very long cylindrical fibers with some twists .

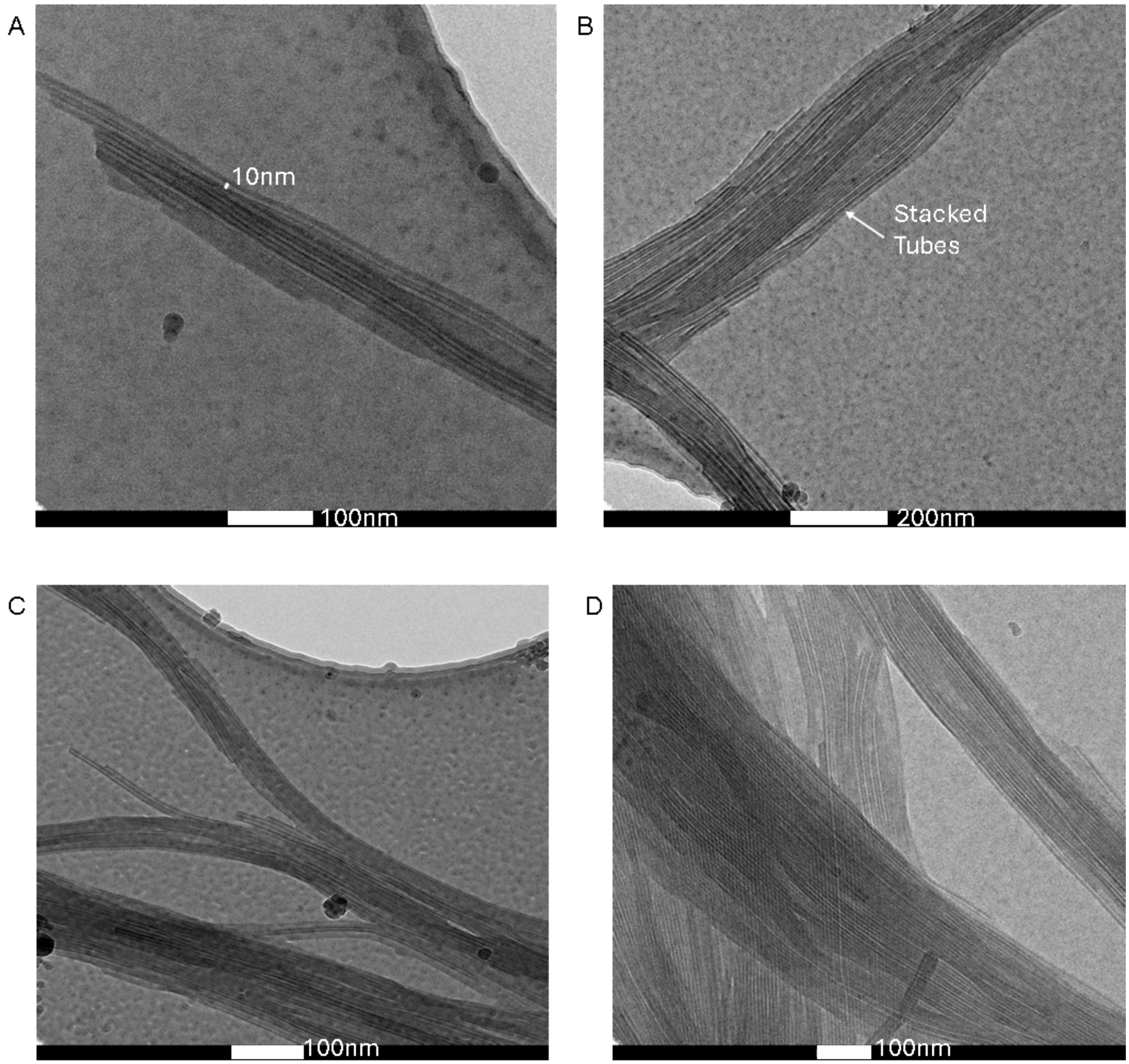

Cryo-TEM of Gelator **2** at minimum gelation concentration (2.5mM) showing hollow tubes stacking together.

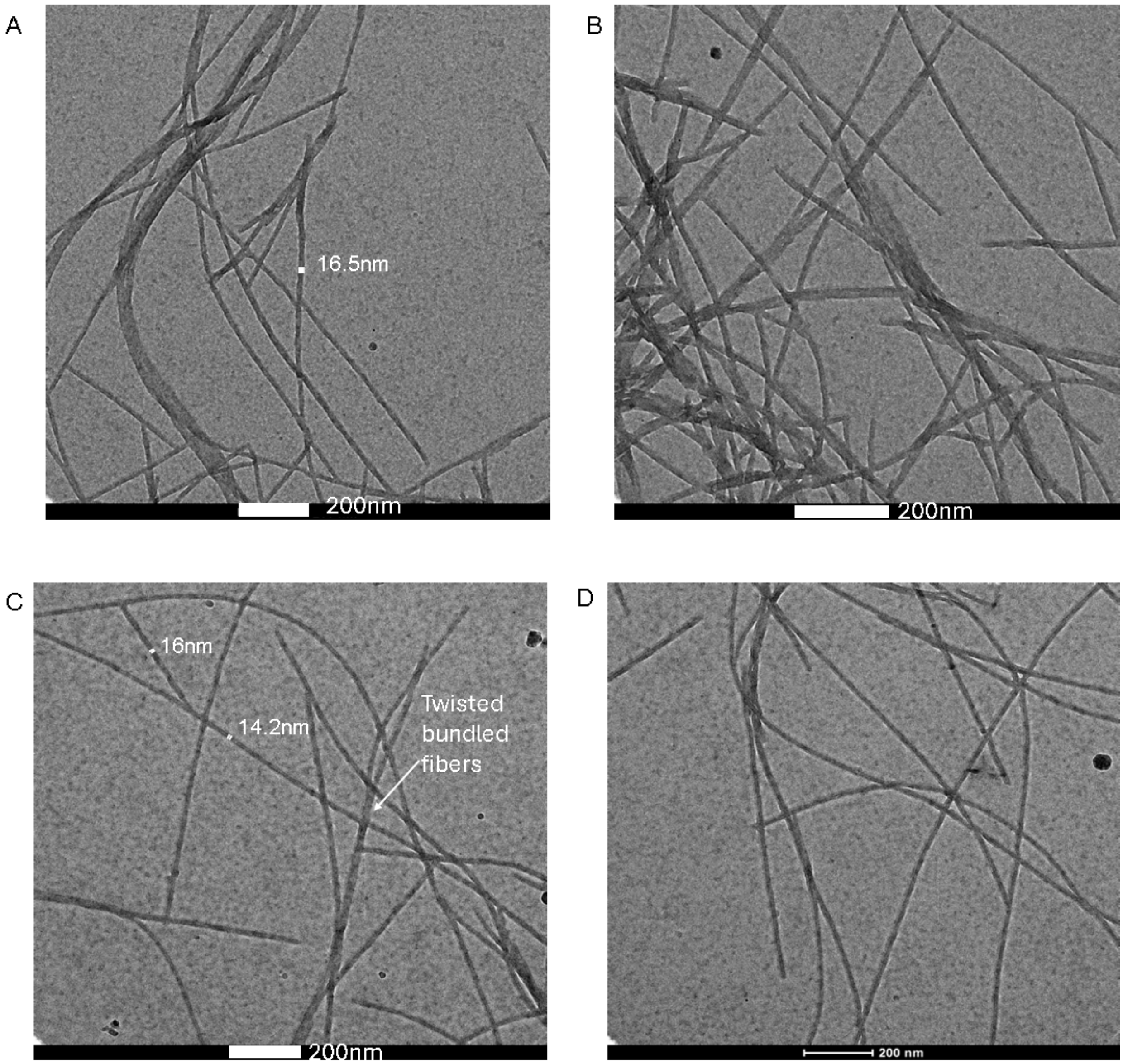

Cryo-TEM of Gelator **3** at minimum gelation concentration showing very flexible cylindrical fibers

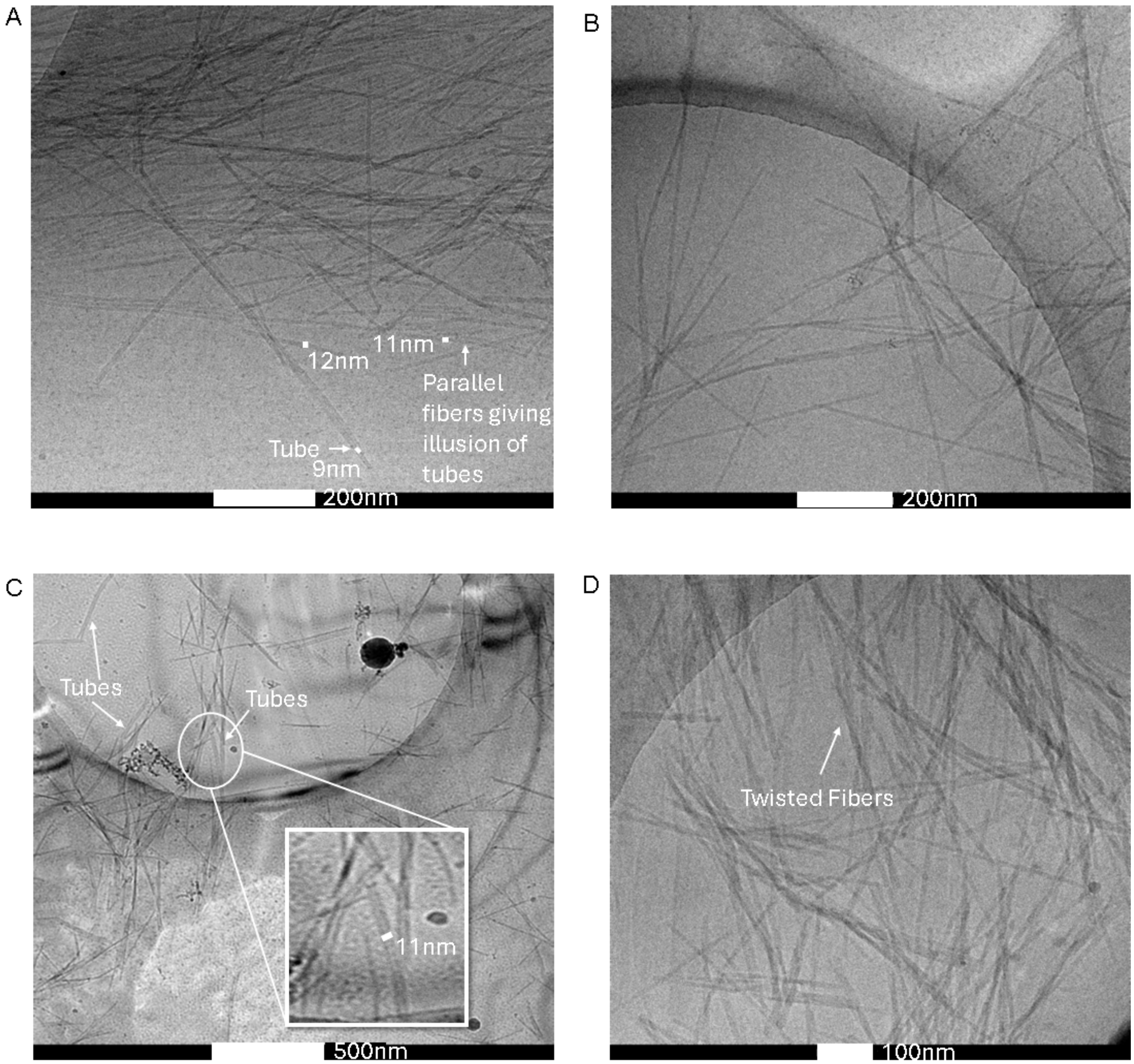

Cryo-TEM of *mix* **1+2** at minimum gelator concentration of each (14mM +2.5mM) showing presence of more cylindrical fibers compared to possibly because of concentration difference among them.

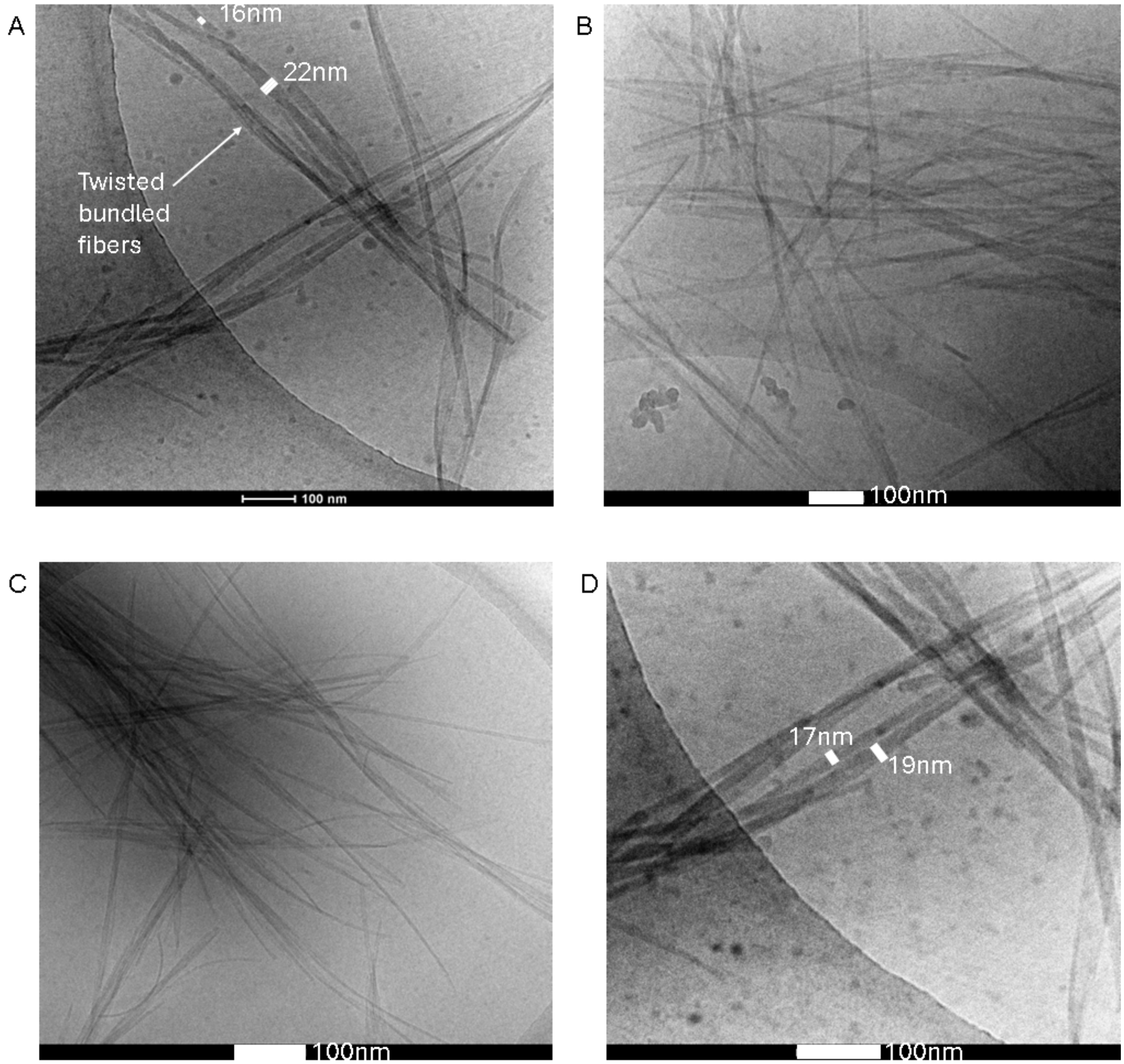

Cryo-TEM images of *mix* **1+3** at minimum gelation concentration of **3** (8.3mM each) showing co-assembled very flexible twisted fibers, thicker than individual gelator.

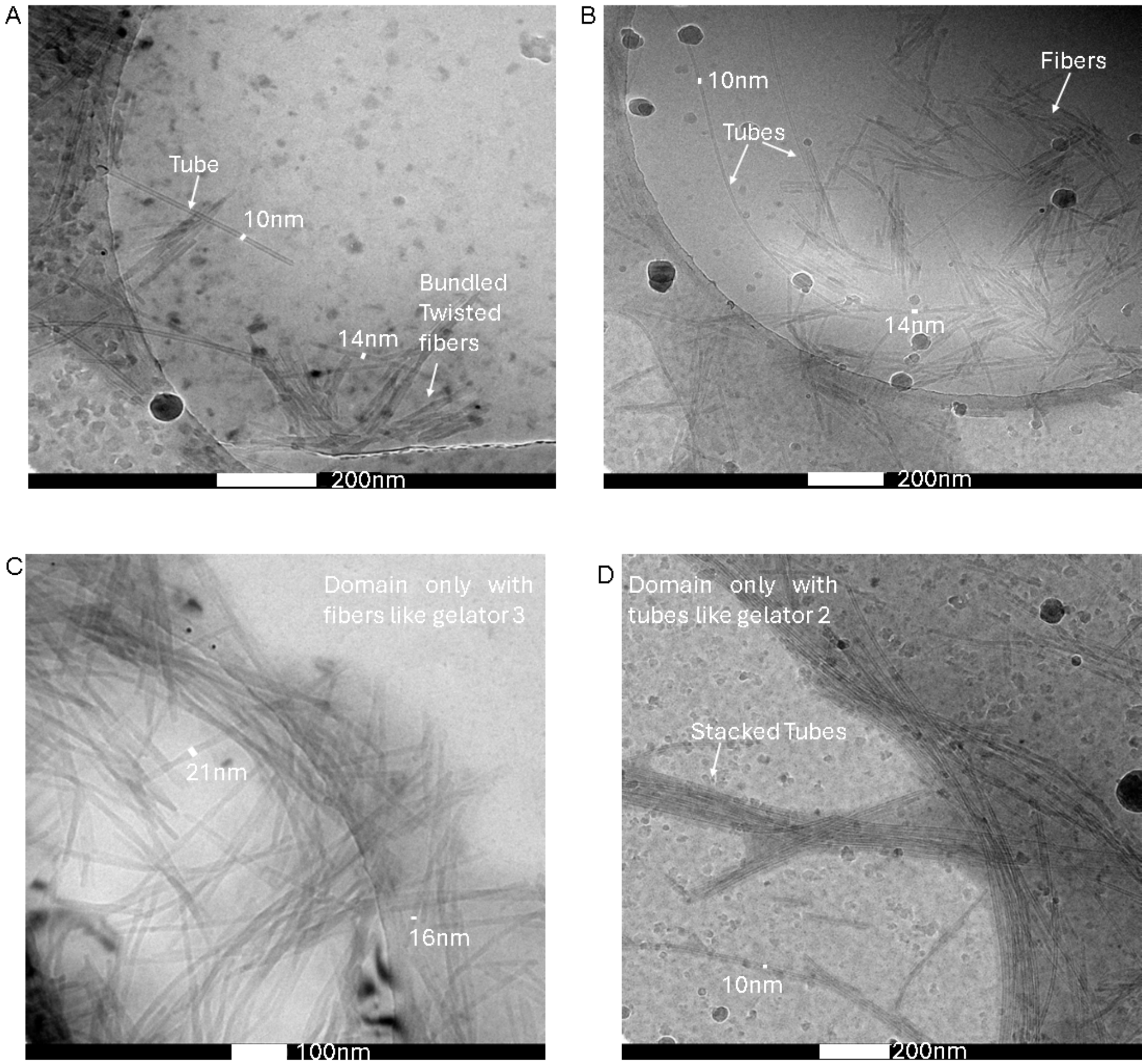

Figure S 9: Cryo-TEM image of *mix* **2+3** at minimum gelation concentration of individual gelator (2.5mM + 8.3mM) showing A-B) Presence of both tube and fibers C) only presence of cylindrical fibers from gelator **3** D) Presence of only tubes from gelator **2**.

11) Rheology Data Analysis

Rheological measurements were conducted on a Discovery HR-30 rheometer from TA instruments using a steel parallel plate-to-plate geometry (40 mm diameter). The gap distance was fixed at 500 µm. The hydrogels were prepared under the desired conditions and the hot solution of samples transferred to the rheometer plate, well-covered with the instrumental metal cover to avoid evaporation and stabilized for 5 minutes before analysis. Viscoelastic characteristics were studied under oscillatory experiments[1]. Frequency and amplitude sweeps were examined at 25 °C. The experimental conditions were arranged by running an amplitude sweep (0.001-100% strain at 1 Hz) and a frequency sweep step (0.001–100 Hz).

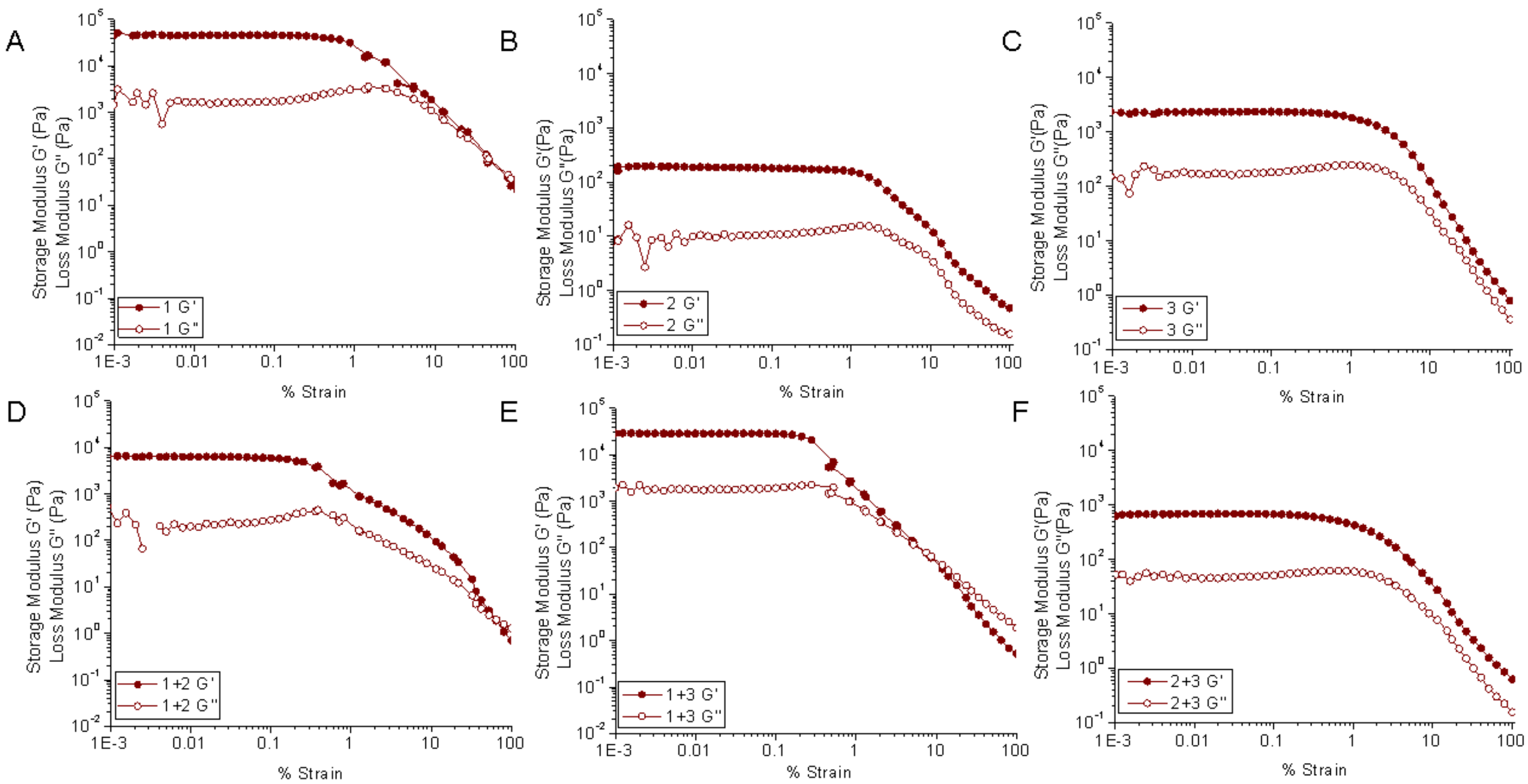

Figure S 10: Amplitude sweeps for one-component and two-component hydrogels at their respective minimum gel concentration (25 °C, 1 Hz). A) Hydrogel **1**, B) Hydrogel **2**, C) Hydrogel **3**, D) Hydrogel *mix* **1+2**, E) Hydrogel *mix* **1 + 3** F) Hydrogel *mix* **2+3**.

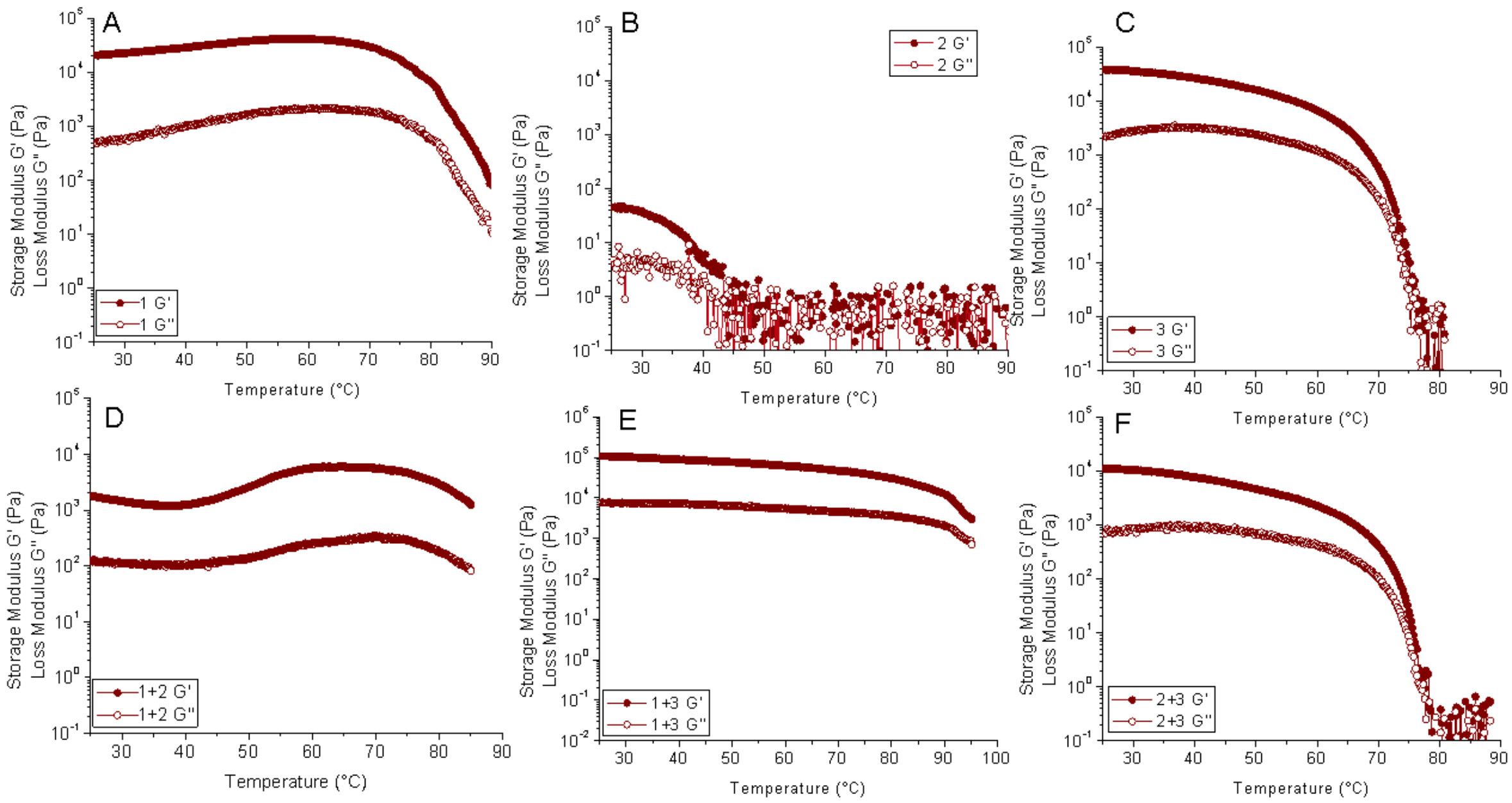

Figure S 11: Temperature ramp for single component hydrogel and dual mixture component hydrogel at 1Hz frequency and diffrent %strain in LVR range. %strain used for gelator **1** -0.025%; gelator **2**- 0.003%; gelator **3**- 0.016%; gelator *mix* **1+2**- 0.04%; gelator *mix* **1+3**- 0.045%; gelator *mix* **2+3**- 0.016% analyzed after 1 hour of gel formation. Increase in G' for component **1** with increase in temperature points towards the entropic charcterstic of the gelator[2].

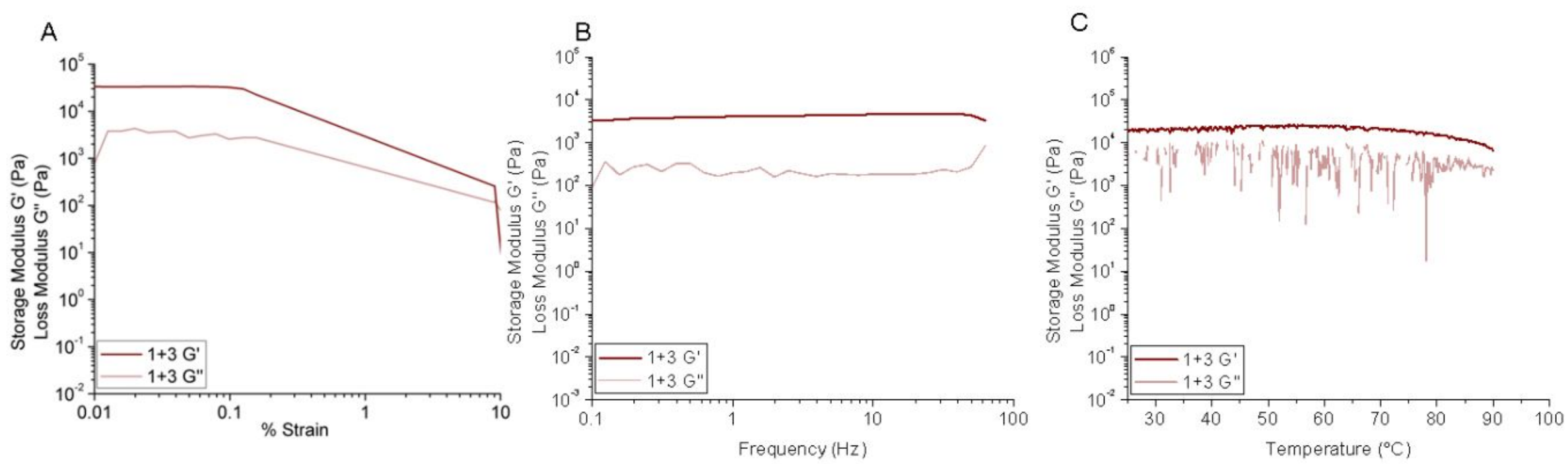

Figure S 12: A) Amplitude Sweep at 25°C, 1Hz frequency. B) Frequency sweep 25°C, 0.012% strain. C) Temperautre Ramp at 1Hz frequency, 0.012% strain of *mix* **1 + 3** at minimum gelation concentration of Gelator-**3** (8.3mM each)

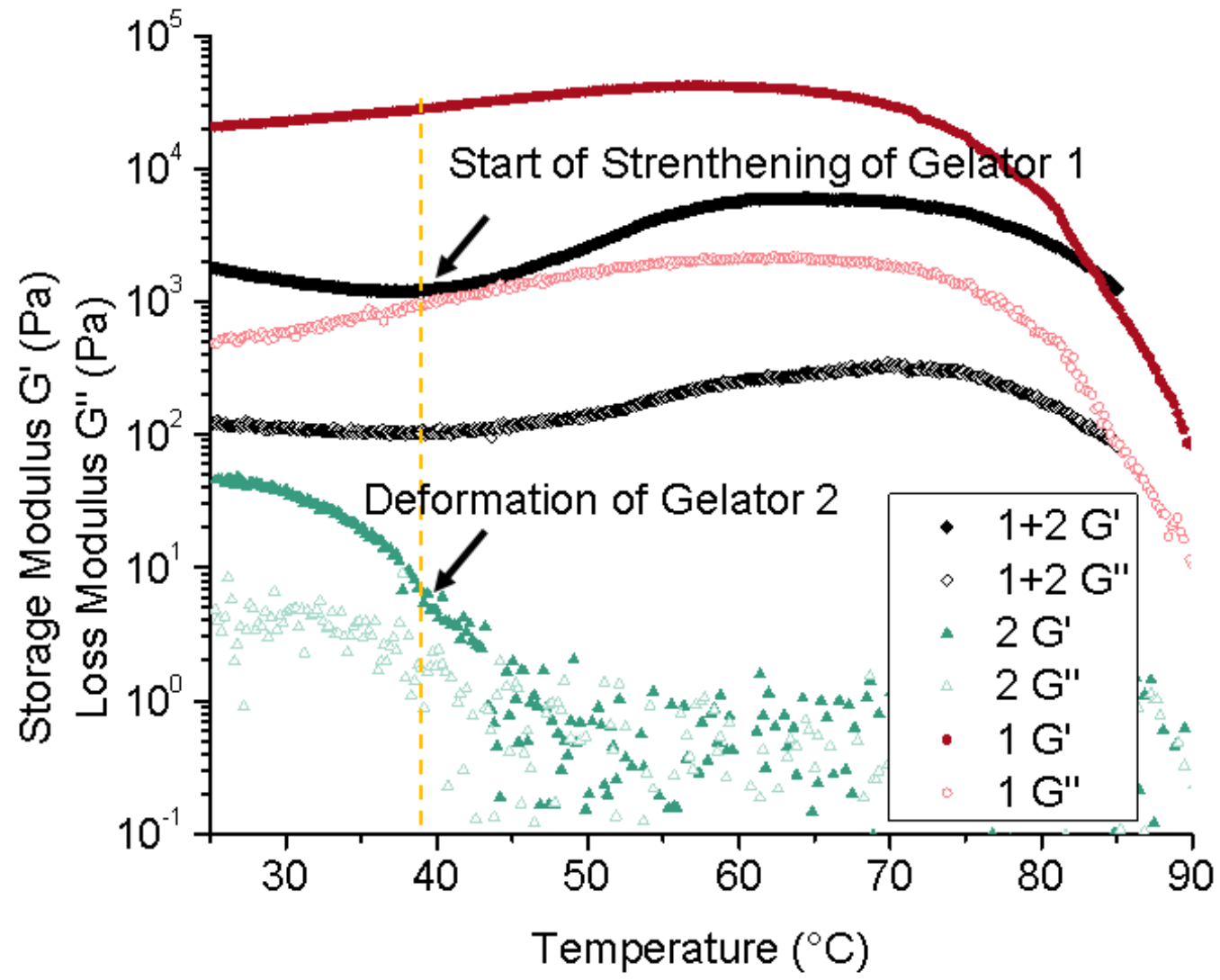

Figure S 13: The comparative temperature ramp of gelator **1**, **2** and *mix* **1+2**. Increase in G' for gelator 1 with increasing temperature is usually defined as stiffening of hydrogel as an entropic behaviour.

Gelators	G' (Pa)	G'' (Pa)	%yield	Tan(δ)	T_{gel} (ºC)
1	5896.5	271.8	0.5	0.046	69.65
2	180.7	10.9	0.8	0.06	36.4
3	5837.7	568.8	0.53	0.1	60.5
Mix 1+2	571.1	25.6	0.25	0.045	69.07
Mix 2+3	676.8	50.6	0.51	0.075	50.65
Mix 1+3	28609.2	1952.9	0.13	0.07	73
Mix 1+3 (1:1)	11206.1	894.62	0.15	0.08	75

Table representing the rheology parameters of different gelators and their combination, G', G'' and % yield has been acquired by amplitude sweep at 1Hz frequency, 25ºC after 1 hour and temperature ramp.from 25 ºC to 85 ºC for respective %strain and 1Hz frequency has been used to get Tgel.at minimum gelation concentration of each gelator alone and when in mixture.

12) DSC data analysis[3]

DSC represents a sensitive thermodynamic technique for the characterization of the energetic landscape of supramolecular assembly in multicomponent hydrogels. Each gelator typically exhibits a characteristic set of transitions, including fiber melting, supramolecular dissociation, glass-transition–like events, and, at higher temperatures, decomposition, that together constitute a thermal “fingerprint” of its assembly behavior. In self-sorted systems, these signatures are largely preserved; multiple, discrete endothermic events associated with the independent disruption of each gelator’s network are evident in the DSC trace, and any glass transition (Tg)–like features remain component-specific, reflecting distinct amorphous or kinetically trapped domains within each independent fibrillar arrangement.

Co-assembled systems, on the other hand, often involve significant reorganization of thermal profiles. Such co-assembly can give rise to broadened or merged endothermic transitions, shifts in melting or dissociation temperatures, and/or the emergence of new transitions not observed in the single-component systems. Importantly, glass-transition–type events may also shift or converge, suggesting altered molecular mobility in a unified supramolecular phase. Such changes result due to the impact of co-assembly on intermolecular packing, local viscosity, and density of states, as well as the generation of mixed amorphous regions with altered relaxation dynamics from either component alone.

Thus, a methodical comparison of the melting transitions, enthalpic events, and glass-transition signatures in DSC thermograms of single-component versus mixed gels directly provides the thermodynamic evidence for self-sorting or co-assembly. Preservation of distinct Tm and Tg values supports self-sorted, compositionally isolated networks, while the appearance of new or shifted transitions reflects cooperative integration of components into one single restructured supramolecular network.

Gelator **1** shows an exothermic effect at around 62 °C right after an endothermic effect at around 50 °C. This effect cannot be seen in the second heating run. Parts of the sample start to decompose at around 200 °C (Figure S 14C). Gelator **2** shows an endothermic effect at 115 °C in the first heating run (Figure S 14A). Gelator **3** shows an endothermic effect in the first heating run at around 97 °C. Also, a glass transition temperature can be seen in the second heating run at around 28 °C. This can mean that the sample solidifies amorphic (Figure S 14B). *Mix* **1+2** shows various endothermic and exothermic effects (Figure S 14F). The first heating run starts with an endothermic effect at about 48 °C and an exothermic effect at 63 °C like Gelator **1**, but the effect has a lower enthalpy. It also shows an endothermic effect at 116 °C which is like Gelator **2**. Further into the measurement at 150 °C there is a small endothermic effect, and another endothermic effect starts at around 180 °C but is not fully shown in the measurement. This effect could be the melting of parts of the sample. In the first and second cooling run an exothermic effect can be seen at 164-184 °C. This could be the crystallization of the melted parts from the unfinished endothermic effects of the heating runs. Also, at the end of the first heating run some parts of the sample could start to decompose which causes the effects to not show off in the next heating run. *Mix* **2+3** shows an endothermic effect at about 90 °C in the first heating run. In the second heating run there is a glass transition temperature with a midpoint at about 28 °C. These effects are like Gelator **3** (Figure S 14E). An endothermic effect starts at about 140 °C and is not fully measured. *Mix* **1+3** shows an endothermic effect at 34 °C which could be the vaporizing of parts of the samples (Figure S 14D). Also, it shows an endothermic effect at around 99 °C in the first heating run which is presumably the melting of the sample. At 140 °C the sample shows an exothermic effect in the first cooling run, which is

presumably the crystallization of the melted parts of the first heating run. In the second heating run an endothermic effect appears at around 133 °C. At the end of the heating runs at around 190 °C the sample starts to decompose. These effects are different to their individual behavior.

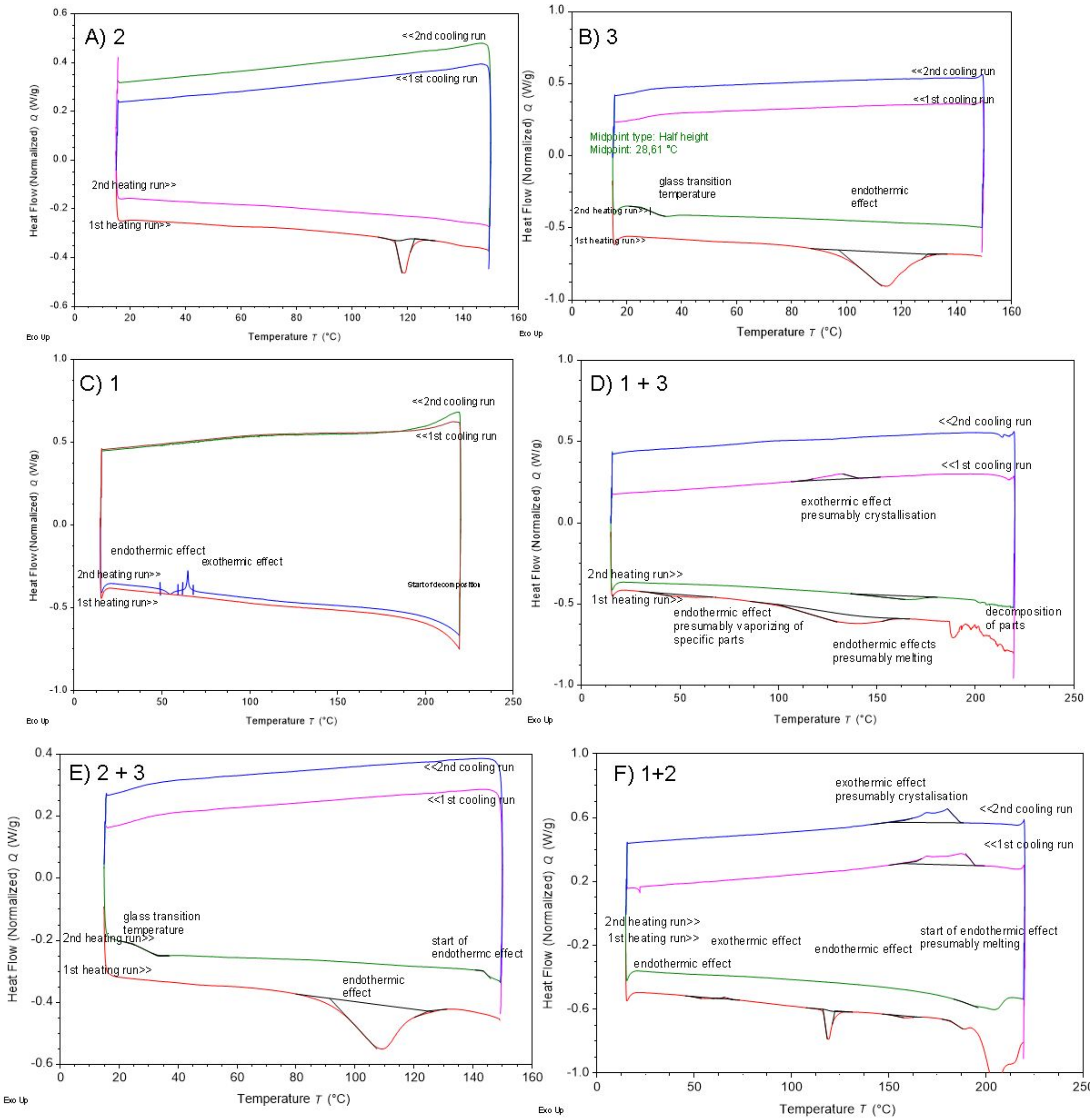

Figure S 14: DSC thermograms (10 °C min^{-1}) of A) **2**, B) **3**, C) **1**, D) *mix* **1+3**, E) *mix* **2+3** and F) *mix* **1+2** during successive heating-cooling cycles, highlighting thermal reversibility and transition enthalpies. The curves visually show endothermic peak for **2** at 119°C and for **3** at 114 °C. and a glass transition for **3** at 28 °C, endothermic peak and exothermic peak for **1** at 54°C, 64 °C respectively.

Gelator	1st Endothermal effect							
	1st heating run				2nd heating run			
	Tonset	Tmax	Toffset	ΔH	Tonset	Tmax	Toffset	ΔH

	[°C]	[°C]	[°C]	[J/g]	[°C]	[°C]	[°C]	[J/g]
**2**	115±1	119±1	122±1	8±2	-	-	-	-
**3**	96±2	114±1	130±1	48±3	-	-	-	-
**1**	50±1	54±1	59±3	2	-	-	-	-
**1 + 2**	48±1	53±1	59±2	1±1	-	-	-	-
**2+3**	91±2	109±1	125±4	30±2	-	-	-	-
**1+3**	28±6	48±5	66±3	3±1	133±6	161±6	174±5	5±1

Table: Results of the DSC measurements 1st endothermal effects

Gelator	1st Exothermal effect							
	1st cooling run				2nd cooling run			
	Tonset	Tmax	Toffset	ΔH	Tonset	Tmax	Toffset	ΔH
	[°C]	[°C]	[°C]	[J/g]	[°C]	[°C]	[°C]	[J/g]
**1**	63±1	64±1	67±4	4±2	-	-	-	-
**1 + 2**	195±3	184±4	162±3	22±4	187±3	178±3	158±4	17±1
**1+3**	138±4	125±10	111±6	5±1	-	-	-	-

Table: Results of the DSC measurements exothermal effects in cooling runs.

Gelator	Glass transition temperatures					
	1st heating run			2nd heating run		
	Tonset	Tmax	Toffset	Tonset	Tmax	Toffset
	[°C]	[°C]	[°C]	[°C]	[°C]	[°C]
**3**	-	-	-	22±3	28±2	33±1
**1+3**	-	-	-	26±1	29±2	32±2

Table: Results of the DSC Measurements glass transition temperature.

Gelator	1st Exothermal effect	
	1st cooling run	2nd cooling run
	Tonset	Tonset
	[°C]	[°C]
**1 + 2**	-	143±1
**2 + 3**	189±2	178±1

Table: Results of the DSC Measurements start of endothermic effects.

Gelator	Endothermal effect							
	1st heating run 2nd effect				2nd heating run 1st effect			
	Tonset	Tmax	Toffset	ΔH	Tonset	Tmax	Toffset	ΔH
	[°C]	[°C]	[°C]	[J/g]	[°C]	[°C]	[°C]	[J/g]
**1 + 2**	117±1	119±1	123±1	9±1	-	-	-	-
**1 + 3**	102±2	125±8	158±2	31±7	133±6	161±6	174±5	5±1

Table:Results of the DSC Measurements endothermal effects in heating runs.

13) SAXS data analysis

The analysis of the SAXS data that is shown in Figure 4 of the main manuscript is described here in-depth. For clarity's sake, we are showing the SAXS data again in Figure S 15.

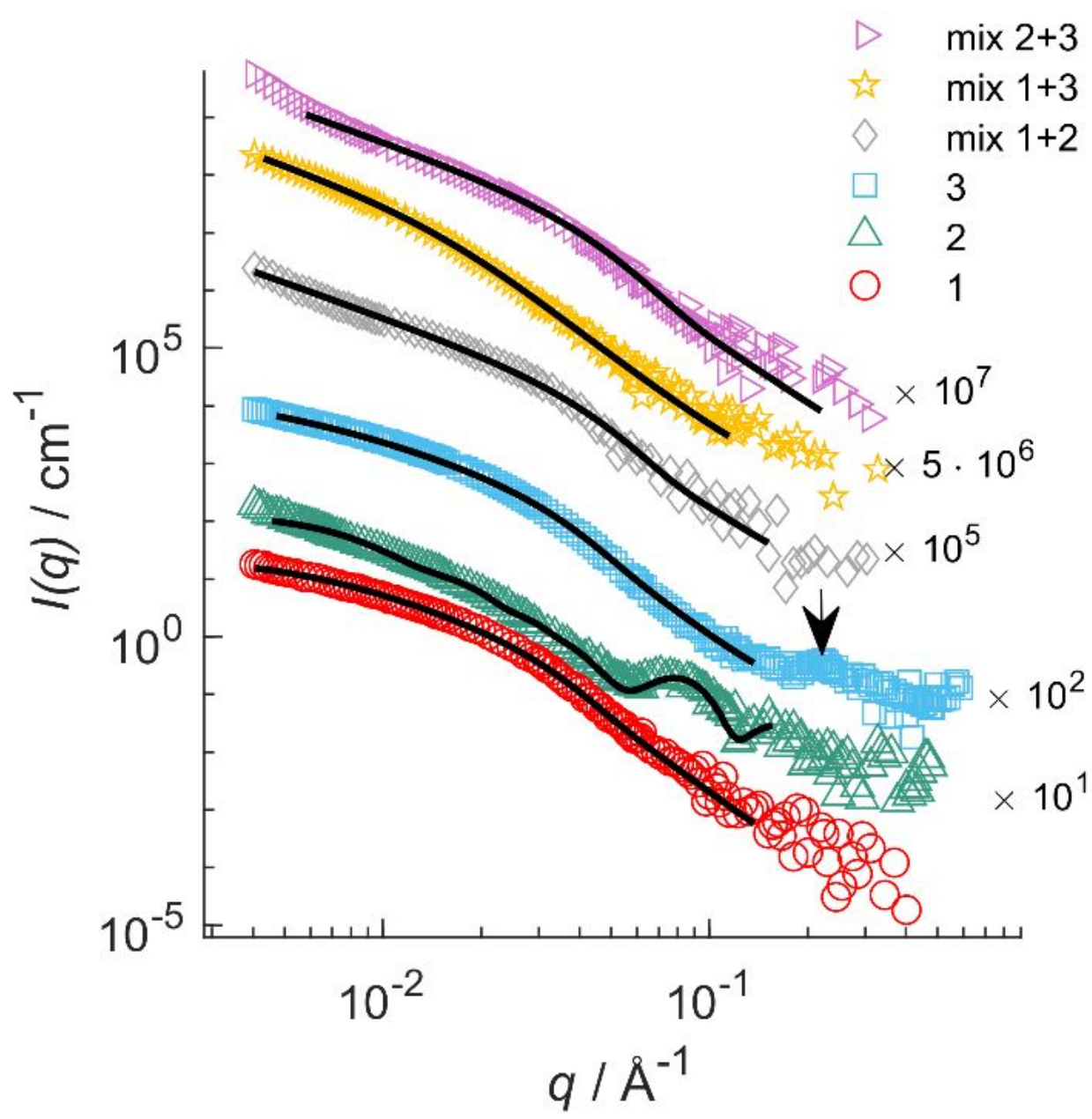

Figure S 15: SAXS data of the single-component hydrogels formed by compounds **1-3** respectively and of the dual-component gels formed by combinations, as described in the main manuscript. The data is also presented in Figure 4 of the main manuscript. Black lines indicate the model curves to each dataset and are described below. The scattered intensities have been multiplied by arbitrary values, displayed next to the curves, for better visualization. The arrow indicates the position of a Bragg peak in the SAXS curve of sample **3**.

Scattering Theory

In a SAXS experiment, the intensity $I(q)$ scattered by a particulate, isotropic dilute sample may be expressed as

$$I(q) \propto P(q) = \int_{-\infty}^{\infty} \rho(\boldsymbol{r}) \exp(i\boldsymbol{qr})\, dV \qquad \text{(EquationS2)}$$

, where the form factor $P(q)$ of the scattering object is given by the Fourier transform of the electron density distribution $\rho(\boldsymbol{r})$ at positions $\boldsymbol{r}$. In the analysis of small-angle scattering data, the goal is to obtain structural information from the observed scattered intensity, a task typically known as the inverse scattering problem. In principle, two approaches may be used to this end. On one hand, the measurement data may be described by suitable model functions that make use of *a priori* knowledge of the system at hand. If the exact nature of the investigated sample is unknown or uncertain, model-free Indirect Fourier Transformation (IFT)[4]

approaches can often be used to reveal the morphology of the sample at hand. These two approaches should be understood as complementary. An initial IFT analysis can often provide valuable insight into a sample's nature that may be used directly for structural analysis, as described below, or to choose a suitable model for a more refined analysis.

Flexible cylinder model

The scattering from flexible, self-avoiding chains has been described previously by Pedersen and Schurtenberger[5] or Chen et al[6]. The first approach, used in this work and implemented in the analysis software SASview (v6.1.0) assumes cylinders with a contour length L far greater than the cross-sectional radius R_{CS} of the cylinders, which allows the separation of the cross section scattering $S_{\mathrm{cs}}(q)$ from that of the flexible chain $S_{\mathrm{chain}}(q)$ with the contour length L and the Kuhn length b.

$$I_{\mathrm{WC}}(q,L,b,R_{\mathrm{cs}}) \propto S_{\mathrm{chain}}(q,L,b) \cdot P_{\mathrm{cs}}(q), \qquad \text{(Equation S3)}$$

where the proportionality constant is governed by the contrast and number density of scattering objects.

The cross-section form factor is given by

$$P_{\mathrm{cs}}(q) = \left[\frac{2J_1(qR_{\mathrm{cs}})}{qR_{\mathrm{cs}}}\right]^2, \qquad \text{(Equation S4)}$$

with $J_1(x)$ as the first order Bessel function.

In the modelling approach by Pedersen and Schurtenberger, they make use of the Daniels approximation [7]by Sharp and Bloomfield[8] and give the scattering from the flexible, chain with excluded volume effects as

$$S_{\mathrm{chain}}(q,L,b) = S_{\mathrm{exv}}\big(q,R_{\mathrm{g}},L,b,\nu\big) + f(q,R_{\mathrm{g}},L,b), \qquad \text{(Equation S5)}$$

, where $S_{\mathrm{exv}}\big(q,R_{\mathrm{g}},L,b,\nu\big)$ gives the structure factore of a chain with excluded volume effects, which depends on the radius of gyration R_{g} of the chain and the Flory exponent ν. The correction term $f(q,R_{\mathrm{g}},L,b)$ accounts for the difference in scattering behaviour from long and short chains, as discussed in the original publication.

Flat lamellar aggregate model

The scattering from a homogeneous, flat object of contrast $(\Delta\rho)^2$ with an in-plane extension that is much larger than its thickness δ, the scattered intensity can be written as[9]

$$I(q) \propto \frac{2\pi}{q^2\delta} \cdot P_{\mathrm{t}}(q) = \frac{2\pi}{q^2\delta} \cdot \frac{4(\Delta\rho)^2}{q^2}\sin^2\left(\frac{q\delta}{2}\right), \qquad \text{(Equation S6)}$$

where a decoupling between the scattering from the membrane with infinite extension ($\propto q^{-2}$) and the thickness form factor $P_{\mathrm{t}}(q)$ was used.

Indirect Fourier Transformation (IFT)

The solution of the inverse scattering problem, i.e. the retrieval of the real-space structure from the scattering curve in reciprocal space may also be performed by Fourier transforming the measured data. However, due to the limited q-range that is experimentally accessible, the integral in Equation S2 cannot be evaluated over the whole range ($0 - \infty$), which leads to termination effects that make a direct transformation impossible. Glatter[4] proposed the concept of indirect Fourier transformation, which reconstructs the model-free pair distance distribution function $p(r)$ from the measured data, taking experimental smearing effects into account. Equation S2 can be rewritten as:

$$I(q) = 4\pi \int_0^\infty p(r) \frac{\sin(qr)}{qr} dr, \qquad \text{(Equation S7)}$$

with the pair distance distribution function $p(r)$ resulting from the spatial autocorrelation function $\gamma(r)$ of the scattering length density distribution $\Delta\rho(r)$ inside the particle.

$$p(r) = r^2\gamma(r) = r^2\Delta\tilde{\rho}^2(r) = r^2 \left\langle \int_V \Delta\rho(\boldsymbol{r_1})\Delta\rho(\boldsymbol{r_1} - \boldsymbol{r})\, d\boldsymbol{r_1} \right\rangle \qquad \text{(Equation S8)}$$

This concept can be further extended to particles with 1D and 2D symmetry. For the case of flat particles, the scattering can be separated into a term $\propto q^{-2}$ and a cross-sectional scattering contribution $I_{\mathrm{t}}(q)$, as shown in Equation S6 and related to the thickness pair distance distribution function $p_{\mathrm{t}}(r)$ by

$$I_{\mathrm{t}}(q) = 2\int_0^\infty p_{\mathrm{t}}(r)\cos(qr)\, dr, \qquad \text{(Equation S9)}$$

which can be further linked to the cross-section scattering length density distribution $\Delta\rho_{\mathrm{t}}(r)$:

$$p_{\mathrm{t}}(r) = 2\int_0^\infty \Delta\rho_{\mathrm{t}}(r')\, \Delta\rho_{\mathrm{t}}(r' - r)\, dr'. \qquad \text{(Equation S10)}$$

Therefore, $\Delta\rho_{\mathrm{t}}(r)$ can be obtained by deconvoluting $p_{\mathrm{t}}(r)$, as suggested by Glatter and Hainisch [10]

Analysis results from model fitting

All SAXS curves, except the one obtained for hydrogel **2**, where modelled with the expressions discussed above. Single-component gels could be successfully fitted to a flexible cylinder model (Equation S3), while a model for flat aggregated was used in the case of the dual-component gels, to get an estimate of typical length scales that govern the high q region (Equation S6). The fitting results are presented in Table below.

	1	3	*mix* 1+2	*mix* 1+3	*mix* 2+3
Model	flexcyl	flexcyl	lam	lam	lam
Scaling	0.0072	0.03	0.0079	0.0092	0.0085
$SLD_{solvent}$ / 10^{-6} Å^2	9.5	9.5	9.5	9.5	9.5
SLD_{fibre} / 10^{-6} Å^2	10.5	10.5	10.5	10.5	10.5
L / Å (PD)	2100 $(0.2)_G$	2100 $(0.2)_G$	n.a.	n.a.	n.a.
b / Å (PD)	92.5 $(0.2)_G$	40 $(0.3)_G$	n.a.	n.a.	n.a.
R_{cs} / Å (PD)	55.2 $(0.4)_G$	51 $(0.4)_G$	n.a.	n.a.	n.a.
δ / Å (PD)	n.a.	n.a.	70 $(0.35)_G$	160 $(0.5)_S$	73 $(0.35)_S$

Table: Model parameters for the single- and multi-component gels that were modelled with a flexible cylinder (flexcyl) or lamellar (lam) model. Displayed are the fitting parameters along with the respective polydispersity that is either described with a Gaussian (G) or Schulz (S) distribution. The variables are described in the sections above.

Indirect Fourier Transformation (IFT analysis) of hydrogel 2

As discussed in the main text, the SAXS curve of hydrogel **2** shows two pronounced form factor oscillations that point towards the existence of a core-shell structure and a low q behaviour that indicates the existence of flat ribbons. Therefore, an IFT analysis was conducted to obtain the pair distance distribution function $p(r)$, shown in Figure S16.

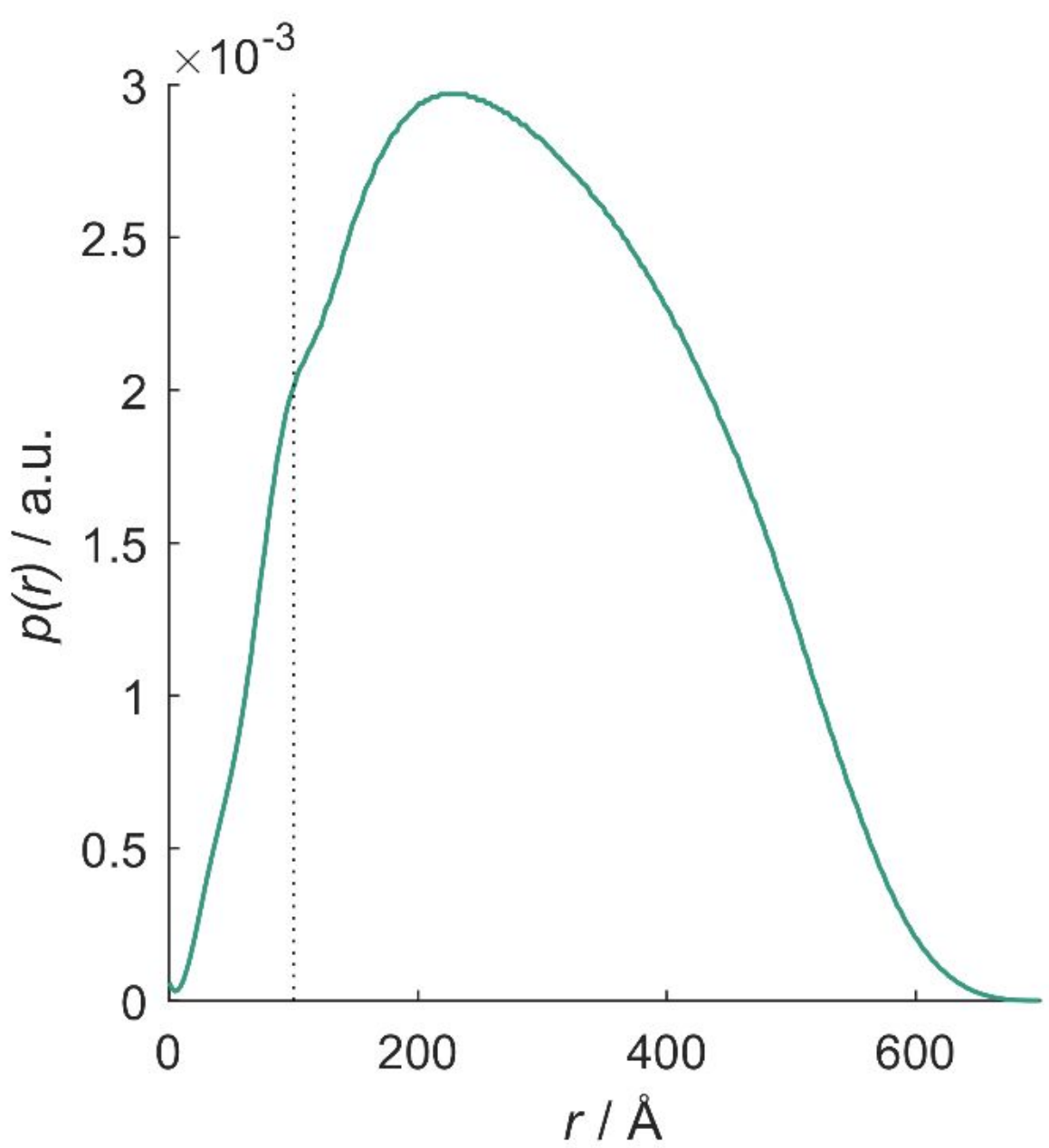

Figure S 16: Pair distance distribution function obtained from the IFT analysis of the SAXS data of the hydrogel formed by compound **2**. The vertical dotted line at $r = 100$ Å indicates a change in curvature of the $p(r)$ function which is an indicator for the ribbon thickness.

The $p(r)$ function rises with r^2 at low r and shows an abrupt change in curvature at $r \approx 100$ Å, which is a sign for a significant structural change at this length scale. It further decays to zero at $r = 680$ Å, where the resolution limit of the experiment is reached. Based on the inspection of the scattering data and the $p(r)$ function, the thickness pair distance distribution function $p_t(r)$ was calculated.

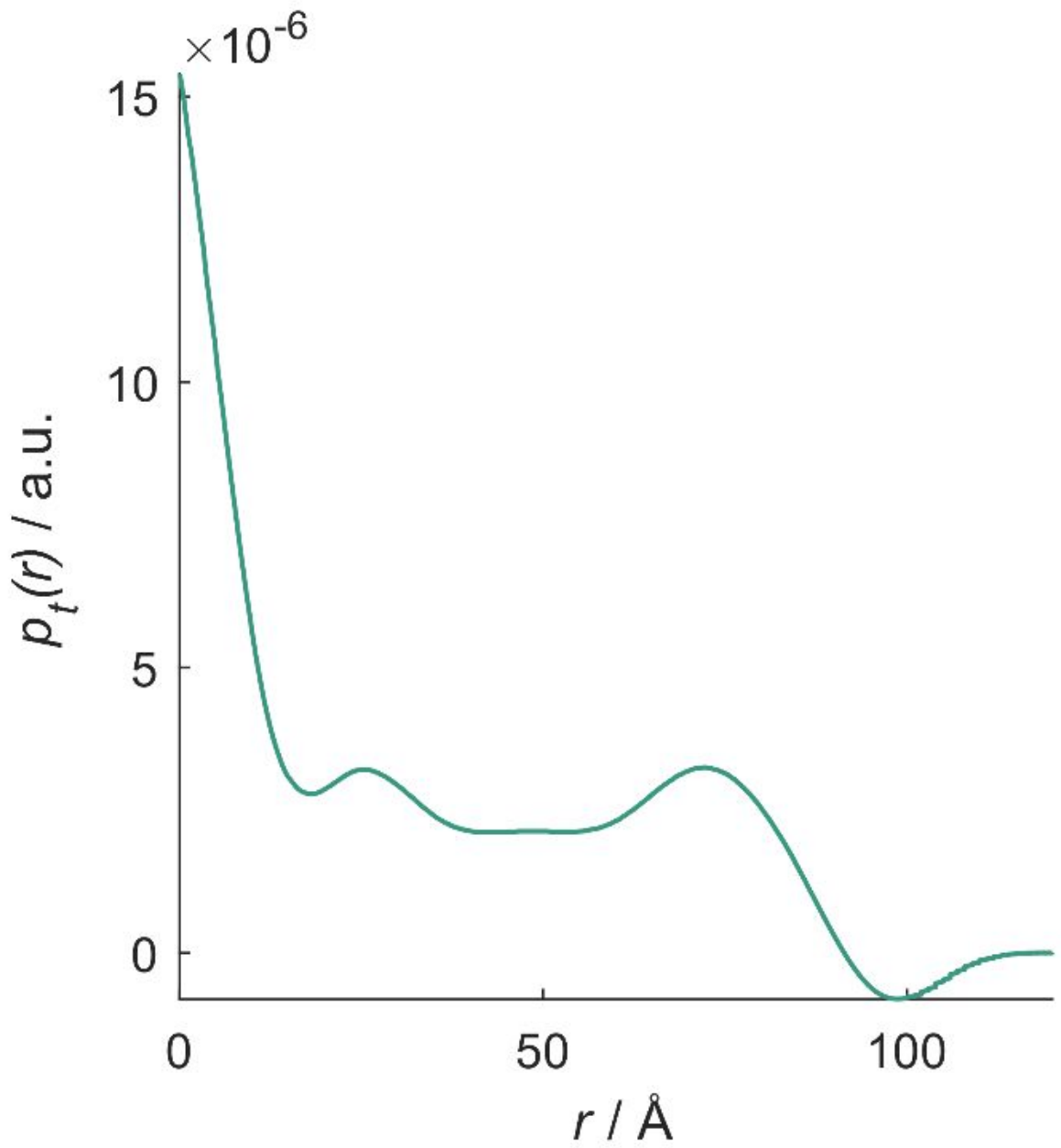

Thickness pair distance distribution function $p_t(r)$, resulting from the IFT analysis of the SAXS data of the hydrogel formed by compound **2** shown in Figure. S 46 under the assumption of a flat lamella.

The function shows the typical signature of an inhomogeneous lamella and decays to zero at a maximum dimension of around 120 Å, giving the thickness δ of the flat ribbon. As a final step, the $p_t(r)$ function was convoluted to give the cross-section electron density distribution $\Delta\rho_t(x)$.

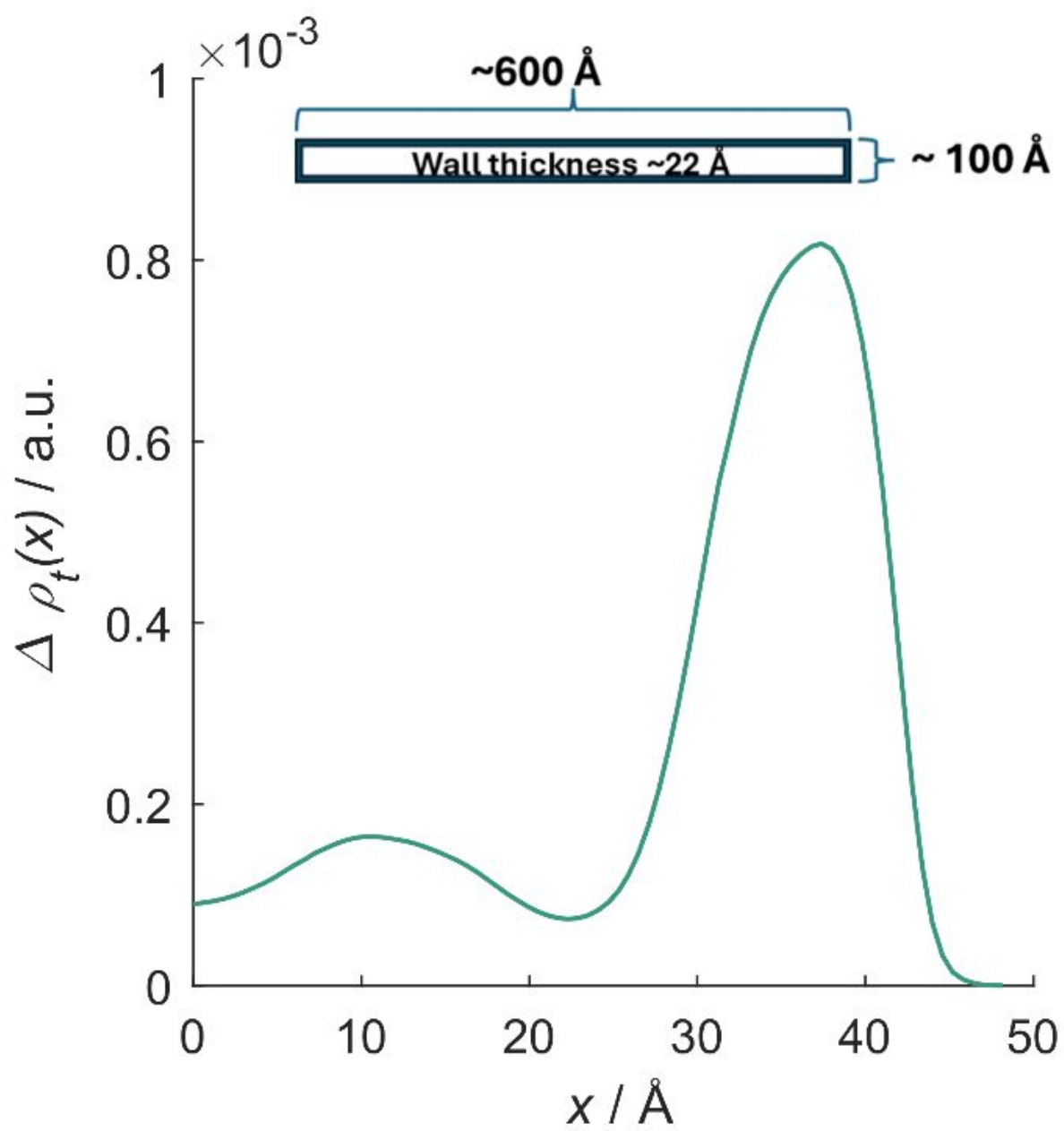

Cross-section electron density contrast $\Delta\rho_t(x)$ at a distance x from the ribbon centre obtained from the deconvolution of the p_t (r) shown in Figure S 48. The inset shows a sketch of the ribbon morphology resulting from the analysis.

As can be seen, the centrosymmetric cross-sectional electron density distribution shows low contrast in the core of the ribbons ($x = 0$ Å). As the edge of the cross-section is approached, the electron density contrast sharply increases and subsequently decays to the solvent level around $x = 50$ Å. Therefore, we conclude that the ribbons possess a hollow core of ca. 60 Å and a wall thickness of ca. 22 Å, resulting in a total thickness of ca. 100 Å. The maximum width of the ribbons can be estimated from the $p(r)$ function to be at least 680 Å, while larger length scales cannot be probed in our SAXS setup due to the low q limit.

Comparison of the Bragg peak of hydrogel 3 with the WAXS measurement of the powder substance

Displayed below is the high-q portion of the SAXS curve of hydrogel **3** in direct comparison with a Wide-angle X-ray scattering (WAXS) measurement of the gelator in its powder, solid form. While the hydrogel showed a Bragg peak at $q = 0.22\ \text{Å}^{-1}$(corresponding repeat distance: 28.6 Å), the first crystalline peak of the powder can be found at $q = 0.266\ \text{Å}^{-1}$ (corresponding repeat distance: 23.6 Å), indicating a slight swelling of the underlying structure, as the fibres are formed in water. The WAXS pattern of the crystalline substance is complicated, but in essence, three sets of equidistant peaks can be observed at $q = \{0.266\ \text{Å}^{-1}, 0.532\ \text{Å}^{-.1}, 0.792\text{Å}^{-1}\}$, $q = \{0.446\ \text{Å}^{-1}, 892\ \text{Å}^{-1}\}$ and finally $q = \{0.587\ \text{Å}^{-1}, 1.163\ \text{Å}^{-1}\}$. The corresponding real-space distances are 23.6 Å , 14.1 Å and 10.7 Å respectively. As mentioned in the main manuscript, the largest distance is close to the maximum molecular length calculated for a closely related class of gelators, while the other distances could correspond to a lamellar backbone-to-backbone organization of gelator molecules due to π-π-stacking of the phenyl side groups, with water bound to the carboxylic acid group.

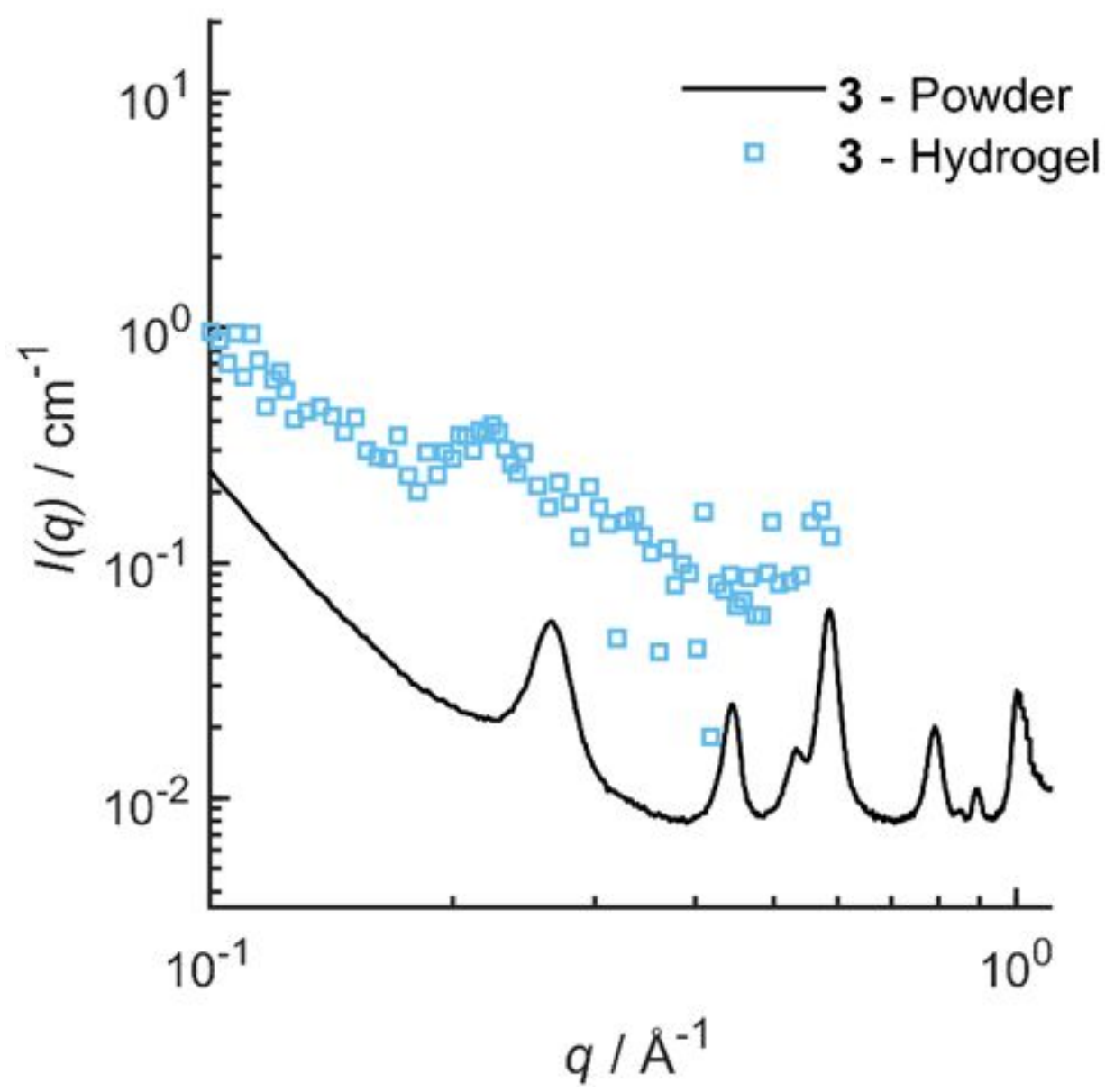

Comparison of the high-q SAXS measurement of the hydrogel sample 3 and the WAXS measurement of the powder substance.

(1) Chen, L.; Morris, K.; Laybourn, A.; Elias, D.; Hicks, M. R.; Rodger, A.; Serpell, L.; Adams, D. J. Self-Assembly Mechanism for a Naphthalene−Dipeptide Leading to Hydrogelation. *Langmuir* **2010**, *26* (7), 5232–5242. https://doi.org/10.1021/la903694a.

(2) Wu, J.; Wu, B.; Xiong, J.; Sun, S.; Wu, P. Entropy-Mediated Polymer–Cluster Interactions Enable Dramatic Thermal Stiffening Hydrogels for Mechanoadaptive Smart Fabrics. *Angew. Chem. Int. Ed.* **2022**, *61* (34). https://doi.org/10.1002/anie.202204960.

(3) Jan H. van Esch; Elmira Ghanbari; Stephen J. Picken. Analysis of Diferential Scanning Calorimetry (DSC): Determining the Transition Temperatures, and Enthalpy and Heat Capacity Changes in Multicomponent Systems by Analytical Model Ftting. *J. Therm. Anal. Calorim.* **2023**, 12393–12409.

(4) Glatter, O. A New Method for the Evaluation of Small-Angle Scattering Data. *J. Appl. Crystallogr.* **1977**, *10* (5), 415–421. https://doi.org/10.1107/S0021889877013879.

(5) Pedersen, J. S.; Schurtenberger, P. Scattering Functions of Semiflexible Polymers with and without Excluded Volume Effects. *Macromolecules* **1996**, *29* (23), 7602–7612. https://doi.org/10.1021/ma9607630.

(6) Chen, W.-R.; Butler, P. D.; Magid, L. J. Incorporating Intermicellar Interactions in the Fitting of SANS Data from Cationic Wormlike Micelles. *Langmuir* **2006**, *22* (15), 6539–6548. https://doi.org/10.1021/la0530440.

(7) Box, G. E. P. Some Theorems on Quadratic Forms Applied in the Study of Analysis of Variance Problems, II. Effects of Inequality of Variance and of Correlation Between Errors in the Two-Way Classification. *The Annals of Mathematical Statistics* **1954**, *25* (3), 484–498. https://doi.org/10.1214/aoms/1177728717.

(8) Sharp, P.; Bloomfield, V. A. Light Scattering from Wormlike Chains with Excluded Volume Effects. *Biopolymers* **1968**, *6* (8), 1201–1211. https://doi.org/10.1002/bip.1968.360060814.

(9) Nallet, F.; Laversanne, R.; Roux, D. Modelling X-Ray or Neutron Scattering Spectra of Lyotropic Lamellar Phases : Interplay between Form and Structure Factors. *Journal de Physique II* **1993**, *3* (4), 487–502. https://doi.org/10.1051/jp2:1993146.

(10) Glatter, O.; Hainisch, B. Improvements in Real-Space Deconvolution of Small-Angle Scattering Data. *J. Appl. Crystallogr.* **1984**, *17* (6), 435–441. https://doi.org/10.1107/S0021889884011894.
